# Rab21 regulates caveolin‐1‐mediated endocytic trafficking to promote immature neurite pruning

**DOI:** 10.15252/embr.202254701

**Published:** 2023-01-23

**Authors:** Mima Shikanai, Shiho Ito, Yoshiaki V Nishimura, Remi Akagawa, Mitsunori Fukuda, Michisuke Yuzaki, Yo‐ichi Nabeshima, Takeshi Kawauchi

**Affiliations:** ^1^ Department of Physiology Keio University School of Medicine Tokyo Japan; ^2^ Department of Aging Science and Medicine, Graduate School of Medicine Kyoto University Kyoto Japan; ^3^ Laboratory of Molecular Life Science Institute of Biomedical Research and Innovation, FBRI, CLIK‐5F Kobe Japan; ^4^ Division of Neuroscience, Faculty of Medicine Tohoku Medical and Pharmaceutical University Sendai Japan; ^5^ Laboratory of Membrane Trafficking Mechanisms, Department of Integrative Life Sciences, Graduate School of Life Sciences Tohoku University Sendai Japan

**Keywords:** caveolin‐mediated endocytosis, early endosomes, neuronal maturation, neuronal migration, Rab21, Membranes & Trafficking, Neuroscience

## Abstract

Transmembrane proteins are internalized by clathrin‐ and caveolin‐dependent endocytosis. Both pathways converge on early endosomes and are thought to share the small GTPase Rab5 as common regulator. In contrast to this notion, we show here that the clathrin‐ and caveolin‐mediated endocytic pathways are differentially regulated. Rab5 and Rab21 localize to distinct populations of early endosomes in cortical neurons and preferentially regulate clathrin‐ and caveolin‐mediated pathways, respectively, suggesting heterogeneity in the early endosomes, rather than a converging point. Suppression of Rab21, but not Rab5, results in decreased plasma membrane localization and total protein levels of caveolin‐1, which perturbs immature neurite pruning of cortical neurons, an *in vivo*‐specific step of neuronal maturation. Taken together, our data indicate that clathrin‐ and caveolin‐mediated endocytic pathways run in parallel in early endosomes, which show different molecular regulation and physiological function.

## Graphical abstract

Rab21 and Rab5 localize to distinct populations of early endosomes and play different roles in endocytic trafficking and *in vivo* neuronal maturation, suggesting heterogeneity in the early endosomal system.

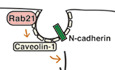

## Introduction

Transmembrane proteins, such as cell adhesion molecules and receptors for secreted molecules, play central roles in intercellular communications in the development and function of multicellular organisms. Proper regulation of their local concentrations on the plasma membrane largely relies on membrane dynamics, such as endocytosis, exocytosis, and intracellular trafficking (Yap *et al*, 2007; Winckler & Mellman, 2010). While clathrin‐mediated endocytosis is reported to cover most of the endocytosis flux in cultured cells (Bitsikas *et al*, 2014), clathrin‐independent endocytic pathways, including caveolin‐mediated, are also important for various cellular events (Howes *et al*, 2010; Parton, 2018; Tobys *et al*, 2021). We have previously reported that caveolin‐1‐mediated endocytosis is required for neuronal maturation during cerebral cortical development, indicating that clathrin‐independent endocytosis has a physiological function *in vivo* (Shikanai *et al*, 2018). However, the regulatory systems of caveolin‐mediated endocytosis are largely unknown.

Internalized transmembrane proteins are transported to the early endosomes. Early endosomes are generally thought as a converging point of various types of endocytic pathways, including clathrin‐mediated and caveolin‐mediated (Singh *et al*, 2003). A small GTPase, Rab5, is known to regulate both clathrin‐ and caveolin‐mediated endocytic pathways. Rab5 promotes endosomal fusion to integrate many endocytic vesicles to the early endosomes. By contrast, several lines of evidence indicate the heterogeneity of the early endosomes *in vitro* (Rink *et al*, 2005; Franke *et al*, 2019). EEA1 and APPL1, Rab5 effector proteins that are markers for the early endosomes (Mu *et al*, 1995; Miaczynska *et al*, 2004), are localized in distinct compartments of the early endosomes in cultured cells (Kalaidzidis *et al*, 2015). However, how this regulates the distinct population of the early endosomes and whether the heterogeneity of the early endosomes leads to different physiological functions are unclear.

During development of the cerebral cortex, postmitotic neurons exhibit multistep modes of migration and maturation (Kawauchi & Hoshino, 2008; Marin *et al*, 2010). Neurons, generated near the ventricle, extend many immature neurites at the intermediate zone of the developing cerebral cortex. Although immature neurites turn into dendrites *in vitro* (Dotti *et al*, 1988), *in vivo* cortical neurons undergo immature neurite pruning, an acute retraction process of immature neurites that is mediated by caveolin‐1 (Shikanai *et al*, 2018), and form a thick leading process, followed by long‐distance neuronal migration along the radial fibers (Ehlers & Polleux, 2010; Kawauchi, 2015). Following the final phase of neuronal migration, the leading processes become branched and mature into dendrites. Interestingly, it is known that each step of neuronal migration and maturation requires different endocytic trafficking pathways (Kawauchi *et al*, 2010; Shieh *et al*, 2011; Nishimura *et al*, 2014; Shikanai *et al*, 2018), and therefore the neuronal migration and maturation in the developing cerebral cortex represents a useful *in vivo* model for analyzing the physiological roles of endocytic pathways.

In this study, we revealed that Rab21, a Rab5 subfamily small GTPase, is localized in the Rab5‐negative early endosomes in cortical neurons and have different physiological functions from Rab5. While Rab5 is known to regulate caveolin‐mediated as well as clathrin‐mediated endocytic pathways *in vitro* (Pelkmans *et al*, 2004; Hagiwara *et al*, 2009; Hayer *et al*, 2010; Ariotti & Parton, 2013), our results indicate that Rab21, but not Rab5, controls caveolin‐mediated endocytosis through maintaining the plasma membrane localization and protein levels of caveolin‐1. Importantly, *in vivo* knockdown of Rab21 in cortical neurons leads to defects in caveolin‐1‐mediated immature neurite pruning, while Rab5 suppression disturbs the long‐distance neuronal migration but does not affect the immature neurite pruning. These data indicate that Rab21 and Rab5 regulate distinct populations of early endosomes, which are required for caveolin‐mediated immature neurite pruning and clathrin‐mediated long‐distance neuronal migration, respectively.

## Results

### Rab21 and Rab5 are localized in the distinct populations of the early endosomes

**Figure 1:**
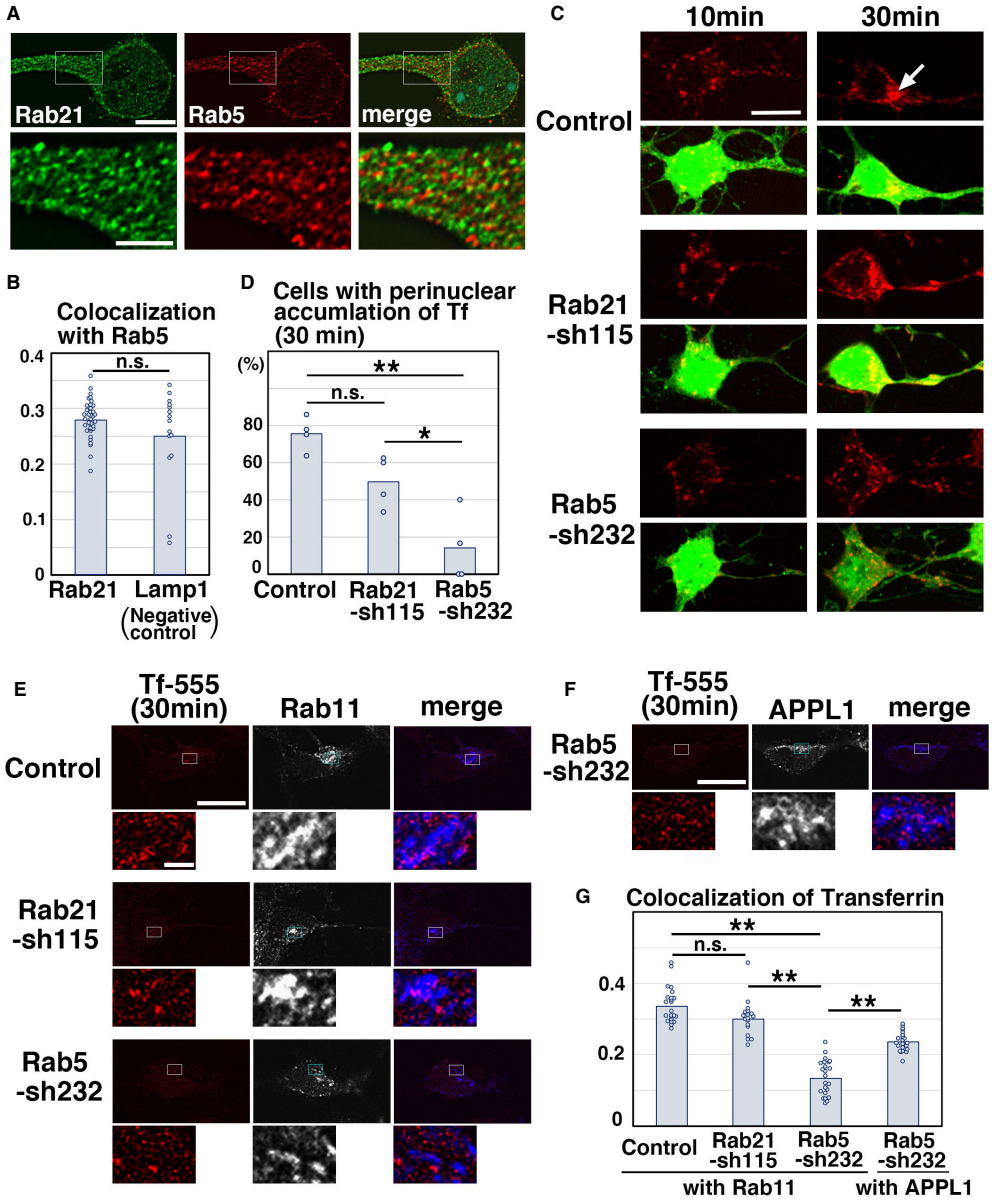
Rab5 and Rab21 have distinct functions *in vitro* A, BPrimary cortical neurons from E15 cerebral cortices incubated for 2 days *in vitro* and stained with the indicated antibodies. Lower panels in (A) are high magnification images indicated by white rectangles in upper panels. The images are obtained with high‐resolution microscopy (Nikon). The graph in (B) shows the Pearson's correlation coefficient of Rab5 and Rab21 or Lamp1 (Lamp1 is a negative control). Each score represents the mean of the individual points. Rab5—Rab21: *n* = 15 cells, Rab5—Lamp1: *n* = 37 cells.C, DPrimary cortical neurons from E15 cerebral cortices were transfected with the indicated plasmids plus pCAG‐EGFP, incubated for 2 days *in vitro* and treated with Tf‐594 for 10 or 30 min before fixation (Green: EGFP, Red: Tf‐594). White arrow indicates the perinuclear accumulation of Tf‐594. The graph in (D) shows the ratio of cells with perinuclear accumulation of Tf‐594 at 30 min after the treatment, which was quantified in a blinded counting. Each score represents the mean of ratios with the individual points. Control: *n* = 4 biological replicates, Rab21‐sh115: *n* = 4 biological replicates, Rab5‐sh232: *n* = 4 biological replicates.E–GPrimary cortical neurons from E15 cerebral cortices were transfected with the indicated plasmids, incubated for 2 days *in vitro* and treated with Tf‐555 for 30 min before fixation. Cells were immunostained with the indicated antibodies. The images are obtained with high‐resolution microscopy (Nikon). Blue alone channels are shown in black and white images. Lower panels in (E) and (F) are high magnification images indicated by white or blue rectangles in upper panels. The graph in (G) shows the Pearson's correlation coefficient of Tf‐555 and Rab11 or APPL1. Each score represents the mean with the individual points. Control: *n* = 23 cells, Rab21‐sh115: *n* = 18 cells, Rab5‐sh232 (Tf—Rab11): *n* = 23 cells, Rab5‐sh232 (Tf—APPL1): *n* = 22 cells. Data information: (B) Significance was determined by Mann–Whitney's *U* test, and no significant difference was observed between control and Rab21‐sh115 (*P* = 0.4986). n.s.: no significant differences. (D) Significance was determined by one‐way ANOVA with *post hoc* Tukey–Kramer. **Less than the critical value at 1%, *less than the critical value at 5%, n.s.: no significant differences. Using the multiple comparison, no significant difference was observed between control and Rab21‐sh115, but when Mann–Whitney's *U* test was applied, we found a significant difference between control and Rab21‐sh115 (*P* = 0.02092). (G) Significance of the Pearson's correlation coefficient between Tf and Rab11 was determined by one‐way ANOVA with *post hoc* Tukey–Kramer. **Less than the critical value at 1%, *less than the critical value at 5%, n.s.: no significant differences. Significance of the Pearson's correlation coefficient in Rab5‐sh232‐transfected cells was determined by Mann–Whitney's *U* test (*P* = 0.00000007396). *P < 0.01. Scale bars: 5 μm in (upper panels in A), 2 μm in (lower panels in A), 10 μm in (C), 10 μm in (upper panels in E, F), 1 μm in (lower panels in E, F).

**Figure EV1:**
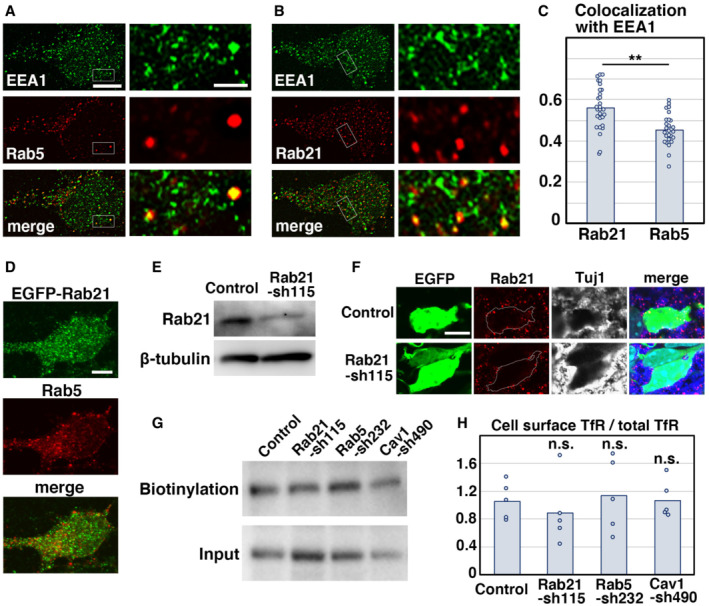
Rab5 and Rab21 localize in distinct vesicular compartments A–CPrimary cortical neurons from E15 cerebral cortices incubated for 2 days *in vitro* and stained with the indicated antibodies. Right panels in (A) and (B) are high magnification images indicated by white rectangles. The images are obtained with high‐resolution microscopy (Nikon). The graph in (C) shows the Pearson's correlation coefficient between EEA1 and Rab21 or Rab5. Each score represents the mean of ratios with the individual points. Rab21: *n* = 29 cells, Rab5: *n* = 28 cells.DPrimary cortical neurons from E15 cerebral cortices were transfected with EGFP‐Rab21 and incubated for 2 days *in vitro*. To maintain moderate expression levels, a CMV promoter was used to express EGFP‐Rab21. Cells were immunostained with anti‐EGFP (green) and anti‐Rab5 antibodies.EPrimary cortical neurons from E15 cerebral cortices were transfected with the indicated plasmids, incubated for 2 days *in vitro* and subjected to immunoblot analyses of cell lysates with the indicated antibodies.FImmature neurons in the IZ of the cerebral cortices at E17, electroporated with the indicated plasmids plus pCAG‐EGFP at E14. Frozen sections were immunostained with the indicated antibodies. The images are obtained with A1R with a high sensitivity GaAsP detector (Nikon). Blue alone channels are shown in black and white images.G, HPrimary cortical neurons from E15 cerebral cortices were transfected with the indicated plasmids, incubated for 2 days *in vitro* and subjected to cell surface biotinylation assay, followed by immunoblot analyses of cell lysates with anti‐TfR antibody. The upper and lower panels indicate the precipitates with streptavidin‐sepharose beads and lysates before pull down (input), respectively. The graph in (H) shows the ratio of cell surface biotinylated transferrin receptor (TfR) to total TfR. Each score represents the mean with the individual points. Control: *n* = 5 biological replicates, Rab21‐sh115: *n* = 5 biological replicates, Rab5‐sh232: *n* = 5 biological replicates, Cav1‐sh490: *n* = 5 biological replicates. Significance was determined by one‐way ANOVA with *post hoc* Dunnett. No significant difference was observed between control and Rab21‐sh115 or Rab5‐sh232 or Cav1‐sh490. n.s., no significant differences. Data information: (C) Significance was determined by Student's *t*‐test (*P* = 0.00007779). **P < 0.01. (H) Significance was determined by one‐way ANOVA with *post hoc* Dunnett. No significant difference was observed between control and Rab21‐sh115 or Rab5‐sh232 or Cav1‐sh490. n.s.: no significant differences. Scale bars: 5 μm in (left panels in A, B), 1 μm in (right panels in A, B), 3 μm in (D), 4 μm in (F).

Rab5 is a central regulator for the trafficking from the plasma membrane to the early endosomes and early endosomal fusion (Zerial & McBride, 2001; Stenmark, 2009). In primary cultured neurons at 2 days‐in‐vitro (DIV) that were derived from mouse cerebral cortex at embryonic day 15 (E15), Rab5 co‐localized well with its effector protein, EEA1, as reported in nonneuronal cells. However, we also found many Rab5‐negative and EEA1‐positive vesicular compartments (Fig EV1A), implying that other regulator(s) may recruit EEA1 on the early endosomes. Among various Rab family small GTPases, Rab21 was found to co‐localize with EEA1, to a similar extent as that of Rab5 and EEA1 (Fig EV1B and C). Interestingly, although Rab5 and Rab21 are reported to co‐localize in nonneuronal cells (Simpson *et al*, 2004; Egami & Araki, 2009), Rab21 was rarely observed in the Rab5‐positive compartments in neurons (Figs 1A and EV1D), with a Pearson's correlation coefficient of < 0.3, similar to the colocalization between Rab5 and a lysosomal marker, Lamp1, a negative control (Fig 1B).

We next analyzed whether Rab21 and Rab5 control the same or different trafficking pathways using a fluorescence dye‐conjugated transferrin (Tf) uptake assay (Trischler *et al*, 1999; Nishimura *et al*, 2014). Transferrin is known to be internalized via clathrin‐mediated endocytosis. We constructed a short hairpin RNA (shRNA) expression vector targeting the coding sequence of mouse Rab21 (Rab21‐sh115), which was confirmed to reduce the expression of endogenous Rab21 efficiently (Fig EV1E and F). For Rab5 knockdown, we utilized the previously reported shRNA for Rab5 (Rab5‐sh232) (Kawauchi *et al*, 2010).

Primary cortical neurons were treated with Alexa594‐conjugated Tf (Tf‐594) and fixed at 10 and 30 min after the treatment. In the control neurons, Tf‐594 was internalized 10 min after the Tf‐594 treatment and transported to the perinuclear recycling endosomes by 30 min (Fig 1C and D). Knockdown of Rab5 resulted in a strong reduction in the ratio of cells with perinuclear accumulation of Tf‐594 (Fig 1C and D). By contrast, Rab21 knockdown had just a partial effect on the endocytic trafficking of Tf‐594 at 30 min after the treatment (Fig 1C and D).

Colocalization of Tf and Rab11, a marker for the perinuclear recycling endosomes, was decreased in the Rab5‐knockdown neurons, whereas knockdown of Rab21 had little effect on the colocalization of Tf and Rab11, compared with the control (Fig 1E and G). In the Rab5‐knockdown neurons, a portion of the internalized Tf accumulated in the APPL1‐positive early endosomes (Fig 1F and G). This suggests that Rab5, rather than Rab21, is involved in the endocytic trafficking of Tf. Consistently, biotinylation of cell surface proteins showed that cell surface levels of Tf receptor (TfR) were not increased in the Rab21‐knockdown neurons (Fig EV1G and 1H). Knockdown of Rab5 only slightly increased the cell surface levels of TfR (Fig EV1G and H), although Rab5 controls the internalization of other transmembrane proteins, such as N‐cadherin, in cortical neurons (Kawauchi *et al*, 2010). These data suggest that Rab21 has a limited role in clathrin‐mediated endocytic pathways, while Rab5 is essential for clathrin‐mediated endocytic pathways.

### Rab21, but not Rab5, is required for immature neurite pruning of cortical neurons

**Figure EV2:**
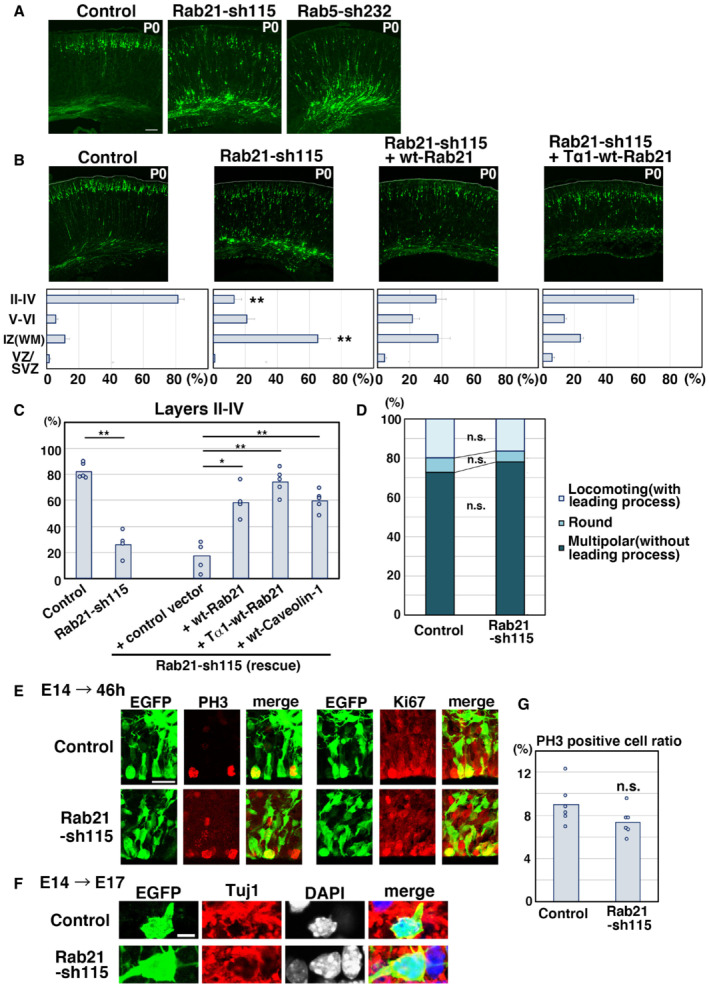
Knockdown of Rab21, as well as Rab5, suppresses cortical neuronal migration, but not the proliferation and differentiation of neural progenitors A, BCerebral cortices at P0, electroporated with the indicated plasmids plus pCAG‐EGFP at E14. The lower graphs in (B) show the estimation of cell migration, which was carried out by recording fluorescence intensities of EGFP in distinct regions of the cerebral cortices using Leica SP5 software. Each bar represents the mean percentage of relative intensities ± SEM. Control: *n* = 6 brains, Rab21‐sh115: *n* = 5 brains, Rab21‐sh115 + pCAG‐wt‐Rab21: *n* = 5 brains, Rab21‐sh115 + pTα1‐wt‐Rab21: *n* = 6 brains. II‐IV, layers II‐IV of the cortical plate; IZ, intermediate zone; SVZ/VZ, subventricular zone/ventricular zone; V‐VI, layers V‐VI of the cortical plate; WM, white matter.CThe ratio of the number of the electroporated cells in the upper layers (layers II–IV). Each score represents the mean of ratios with the individual points. Control: *n* = 5 brains, Rab21‐sh115: *n* = 4 brains, Rab21‐sh115 + control vector: *n* = 5 brains, Rab21‐sh115 + CAG‐wt‐Rab21: *n* = 4 brains, Rab21‐sh115 + Tα1‐wt‐Rab21: *n* = 5 brains, Rab21‐sh115 + wt‐Caveolin‐1: *n* = 5 brains. The sample numbers show biological replicates. In case of co‐electroporation with wt‐Rab21 driven by CAG promoter, the Rab21‐sh115‐mediated migration defect was partially rescued, but its efficiency was lower than that of Tα1‐wt‐Rab21, implicating that excess Rab21 in neural progenitors might have a negative effect on proper neuronal positioning.DCerebral cortices at E17, electroporated with the indicated plasmids plus pCAG‐EGFP at E14. The graph shows the ratio of cells with the indicated morphology in the IZ. Control and Rab21‐sh115: *n* = 3 brains.E–GCerebral cortices at E16 (46 h after electroporation) (E and G) and E17 (F), electroporated with the indicated plasmids plus pCAG‐EGFP at E14. Frozen sections were immunostained with anti‐EGFP and anti‐phospho‐Histone H3 (left panels in E) or anti‐Ki67 (right panels in E) or anti‐Tuj1 (F) antibodies. Blue alone channels are shown in black and white images. The graph in (G) shows the ratio of phospho‐Histone H3‐positive cells in the electroporated cells in the VZ. Each score represents the mean of ratios with the individual points. Control: *n* = 6 brains (762 cells), Rab21‐sh115: *n* = 6 brains (374 cells). Data information: (B) Significance compared to control was determined by Student's *t*‐test (Rab21‐sh115 [Layer II–IV]: *P* = 0.000001254, Rab21‐sh115 [IZ]: 0.00008495). **P < 0.01. (C) Significance was determined by one‐way ANOVA with *post hoc* Tukey–Kramer test. *Less than the critical value at 5% (Control vs. Rab21‐sh115 + ppCAG‐wt‐Rab21, Control vs. Rab21‐sh115 + pCAG‐wt‐Caveolin‐1), **less than the critical value at 1% (Control vs. Rab21‐sh115, Control vs. Rab21‐sh115 + control vector, Rab21‐sh115 vs. Rab21‐sh115 + pCAG‐wt‐Rab21, Rab21‐sh115 vs. Rab21‐sh115 + pTα1‐wt‐Rab21, Rab21‐sh115 vs. Rab21‐sh115 + pCAG‐wt‐Caveolin‐1, Rab21‐sh115 + control vector vs. Rab21‐sh115 + pCAG‐wt‐Rab21, Rab21‐sh115 + control vector vs. Rab21‐sh115 + pTα1‐wt‐Rab21, Rab21‐sh115 + control vector vs. Rab21‐sh115 + pCAG‐wt‐Caveolin‐1). (D) No significant differences (n.s.) between control and Rab21‐sh115‐electroporated neurons were found by Mann–Whitney's U test and Student's *t*‐test (Locomotion: *P* = 0.5127 or 0.4841, Round: *P* = 0.1266 or 0.1916, Multipolar: *P* = 0.2752 or 0.1786, respectively). (G) Significance compared to control was determined by Student's *t*‐test (*P* = 0.1149). n.s.: no significant differences. Scale bars: 100 μm in (A, B). 25 μm in (E), 5 μm in (F).

Given that Rab21 and Rab5 regulate distinct populations of the early endosomes in neurons, we next examined their *in vivo* function in the developing cerebral cortex. Our previous studies indicated that Rab5 is required for the radial fiber‐dependent long‐distance migration of cortical neurons (Kawauchi *et al*, 2010; Nishimura *et al*, 2014), but an *in vivo* role for Rab21 is not known. To test this, Rab21‐sh115 was electroporated into mouse cerebral cortices at E14. The electroporated cells were visualized with co‐transfected CAG promoter‐driven EGFP (pCAG‐EGFP). At postnatal day 0 (P0), 5 days after electroporation, Rab21‐sh115‐electroporated cells exhibited severe neuronal migration defects, similar to the Rab5 knockdown, whereas control cells were observed to migrate normally to the superficial layer of the cortical plate (Fig EV2A). The migration defects of the Rab21 knockdown cells were rescued by a ubiquitous CAG promoter‐ and a neuron‐specific Tα1 promoter‐driven wild‐type (wt)‐Rab21 (Fig EV2B and C), suggesting that expression of Rab21 in neurons is required for proper neuronal migration. Consistent with this, Rab21‐sh115 did not affect the expression of Tuj1 (a neuronal marker) in the neurons and proliferative markers, phospho‐histone H3 (PH3) and Ki67, in the neural progenitors (Fig EV2E–G).

**Figure 2:**
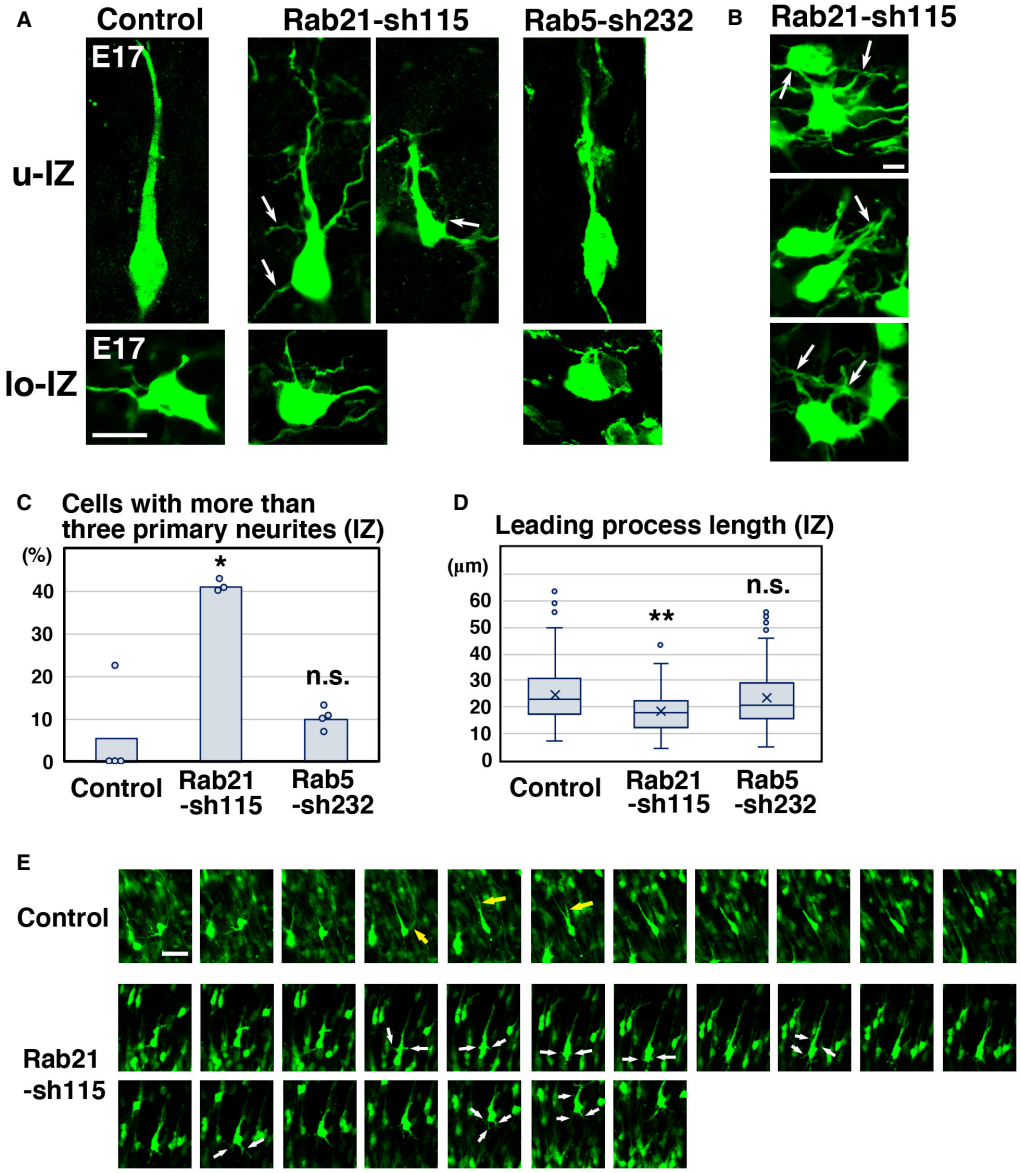
Rab5 and Rab21 have distinct functions *in vivo* A–DCerebral cortices at E17, electroporated with the indicated plasmids plus pCAG‐EGFP at E14. (A) High magnification images of the upper intermediate zone (u‐IZ) or lower intermediate zone (lo‐IZ) of the cerebral cortices. White arrows indicate abnormal primary neurites. (B) High magnification images of the lower IZ of the cerebral cortices. White arrows indicate sticky neurites. (C) The ratio of locomoting neurons with more than three primary neurites in the IZ. Each score represents the mean with the individual points. Control: *n* = 4 brains (60 cells), Rab21‐sh115: *n* = 3 brains (65 cells), Rab5‐sh232: *n* = 4 brains (111 cells). (D) The box‐and‐whisker plot shows average leading process length of the locomoting neurons in the IZ. Control: *n* = 100 cells, Rab21‐sh115: *n* = 80 cells, Rab5‐sh232: *n* = 111 cells.ETime‐lapse imaging of control and Rab21‐sh115‐electroporated cells in cortical slices from E16 cerebral cortices, electroporated with the indicated plasmids at E14. After formation of the leading process, control neurons rapidly eliminated their immature neurites (yellow arrows), whereas the Rab21‐knockdown neurons retained the immature neurites (white arrows) for long periods. Time interval of each frame is 30 min. Data information: (C) Significance compared to control was determined by Mann–Whitney's *U* test (Control vs. Rab21‐sh232: *P* = 0.02771, Control vs. Rab5‐sh232: *P* = 0.2367) and Welch's *t*‐test (Control vs. Rab21‐sh232: *P* = 0.007987, Control vs. Rab5‐sh232: *P* = 0.4819). *P < 0.05, n.s.: no significant differences. (D) In the box‐and‐whisker plots, the central band and the upper and lower sides of the boxes indicate the median and the upper and lower quartiles. The whiskers of the depicted boxplots go from the minimum to the lower quartile and from the upper quartile to the maximum. “x” indicates the average value. Significance compared to control was determined by Mann–Whitney's *U* test (Control vs. Rab21‐sh232: *P* = 0.00006332, Control vs. Rab5‐sh232: *P* = 0.5213) and Welch's *t*‐test (Control vs. Rab21‐sh232: *P* = 0.00001667, Control vs. Rab5‐sh232: *P* = 0.4581). **P < 0.01, n.s.: no significant differences. Scale bars: 10 μm in (A), 2 μm in (B), 20 μm in (E).

To more precisely elucidate the phenotypes of Rab21 or Rab5 knockdown neurons, we performed morphological analyses on the knockdown neurons in the cerebral cortices at E17, 3 days after electroporation. At this stage, control neurons exhibited multipolar or round or spindle (locomoting) morphology. The ratio of these cell types was not significantly changed between control and Rab21‐knockdown neurons (Fig EV2D), whereas Rab5 knockdown is reported to increase the ratio of cells with round morphology (Kawauchi *et al*, 2010). However, the immature neurites of Rab21 knockdown multipolar‐shaped neurons were sometimes observed to be attached to one another (Fig 2B), in contrast to Rab5 knockdown.

While control spindle‐shaped neurons with a leading process (locomoting neurons) showed bipolar or unipolar morphology, the Rab21 knockdown neurons extended not only a leading process and an axon but also many additional neurites (Fig 2A and C). Our time‐lapse imaging analyses showed that these abnormal neurites frequently extended and retracted, which is a major characteristic of the immature neurites of the multipolar‐shaped neurons (Tabata & Nakajima, 2003; Noctor *et al*, 2004; Kawauchi *et al*, 2006; Nishimura *et al*, 2010; Fig 2E). This suggests that these additional neurites of Rab21‐knockdown neurons are derived from the immature neurites. We did not observe an acute retraction of the immature neurites in Rab21‐knockdown neurons, which exhibit the phenotype seen in caveolin‐1‐knockdown neurons (Shikanai *et al*, 2018), and therefore we referred to this phenotype as an immature neurite pruning defect. By contrast, Rab5‐sh232‐electroporated neurons exhibited a normal bipolar or unipolar morphology, similar to control (Fig 2A). These results indicate that knockdown of Rab21, but not Rab5, causes defects in the immature neurite pruning during the multipolar‐to‐bipolar transition of immature cortical neurons.

In addition, electroporation of Rab21‐sh115, but not Rab5‐sh232, into neurons resulted in formation of a significantly shorter leading process, compared with control (Fig 2D). Because these phenotypes of Rab21 knockdown neurons resembled that of caveolin‐1 knockdown (Shikanai *et al*, 2018), we next analyzed the relationship between Rab21 and caveolin‐1.

### Rab21 preferentially co‐localizes with caveolin‐1

**Figure 3:**
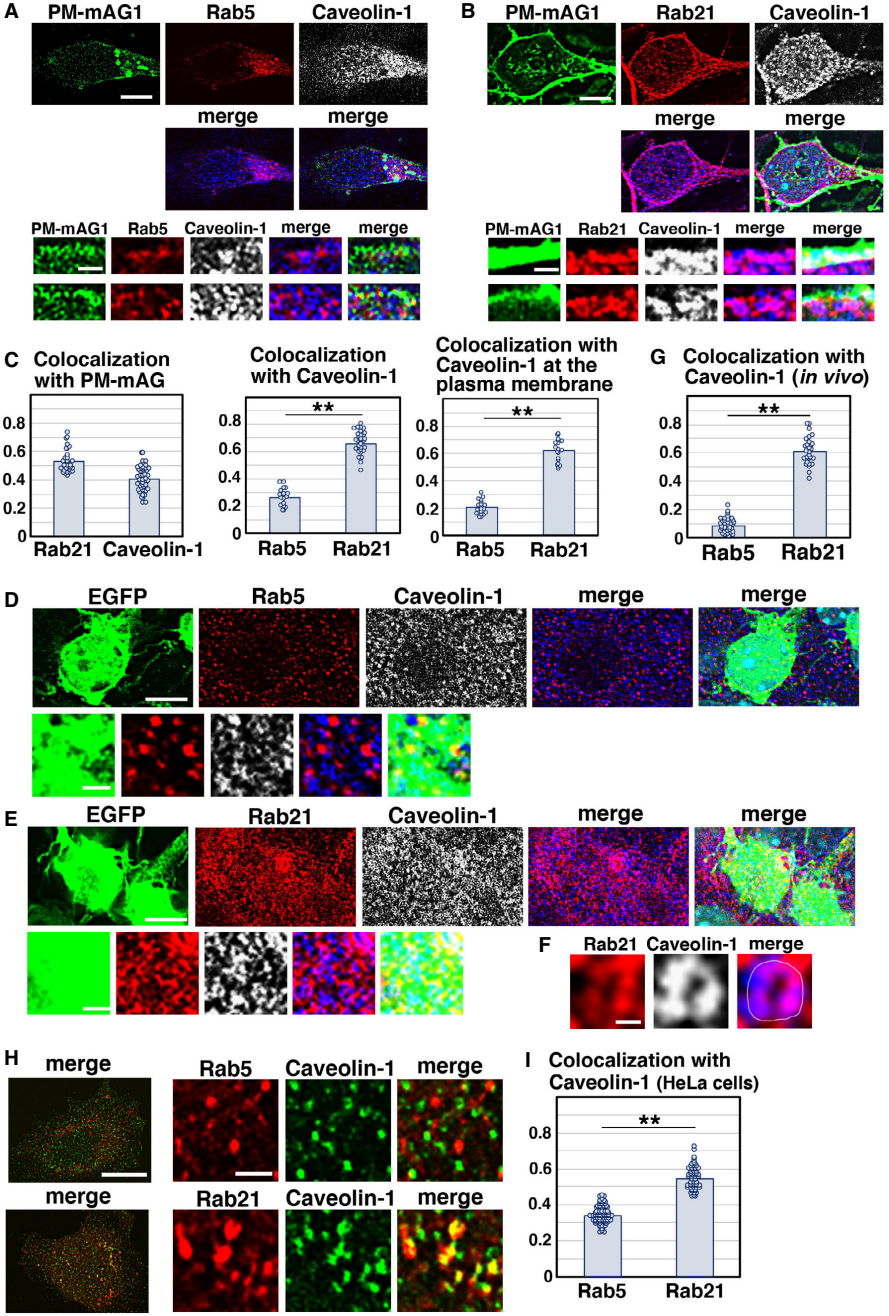
Rab21 prefers to co‐localize with caveolin‐1 in cortical neurons A–CPrimary cortical neurons from E15 cerebral cortices were transfected with pCAG‐PM‐mAG1 (green) and incubated for 2 days *in vitro*. Cells were immunostained with the indicated antibodies (red and blue/white). The transfected PM‐mAG1 is a marker for the plasma membrane. The images are obtained with high‐resolution microscopy (Nikon) and the lower panels are high magnification images near the plasma membrane. Blue alone channels are shown in black and white images. The graphs in (C) show the Pearson's correlation coefficient between PM‐mAG1 and Rab21 or Caveolin‐1 (left) and between caveolin‐1 and Rab5 or Rab21 in whole cells (middle) or at the plasma membrane (right). Each score represents the mean of ratios with the individual points. Rab5: *n* = 18 cells (middle and right), Rab21: *n* = 31 cells (left and middle) or 20 cells (right), Caveolin‐1: *n* = 43 cells (left).D–GImmature neurons in the IZ of the cerebral cortices at E17, electroporated with pCAG‐EGFP at E14. Frozen sections were immunostained with the indicated antibodies. The images are obtained with high‐resolution microscopy (Nikon). Blue alone channels are shown in black and white images. The images in (F) are high magnification images of (E). The graph in (G) shows the Pearson's correlation coefficient between caveolin‐1 and Rab5 or Rab21. Each score represents the mean of ratios with the individual points. Rab5: *n* = 38 cells, Rab21: *n* = 28 cells.H, IHeLa cells were immunostained with the indicated antibodies. The images are obtained with high‐resolution microscopy (Nikon). The graph in (I) shows the Pearson's correlation coefficient between caveolin‐1 and Rab5 or Rab21. Each score represents the mean of ratios with the individual points. Rab5: *n* = 80 cells, Rab21: *n* = 62 cells. Data information: (C) Significance was determined by Student's *t*‐test (Middle: *P* = 9.144E‐22, Right: *P* = 2.309E‐19). **P < 0.01. (G) Significance was determined by Welch's *t*‐test (*P* = 1.225E‐25). **P < 0.01. (I) Significance was determined by Welch's *t*‐test (*P* = 3.209E‐40). **P < 0.01. Scale bars: 3 μm in (upper panels in A, B, D, E), 0.5 μm in (lower panels in A, B, D, E), 0.1 μm in (F), 10 μm in (left panels in H), 1 μm in (right panels in H).

**Figure EV3:**
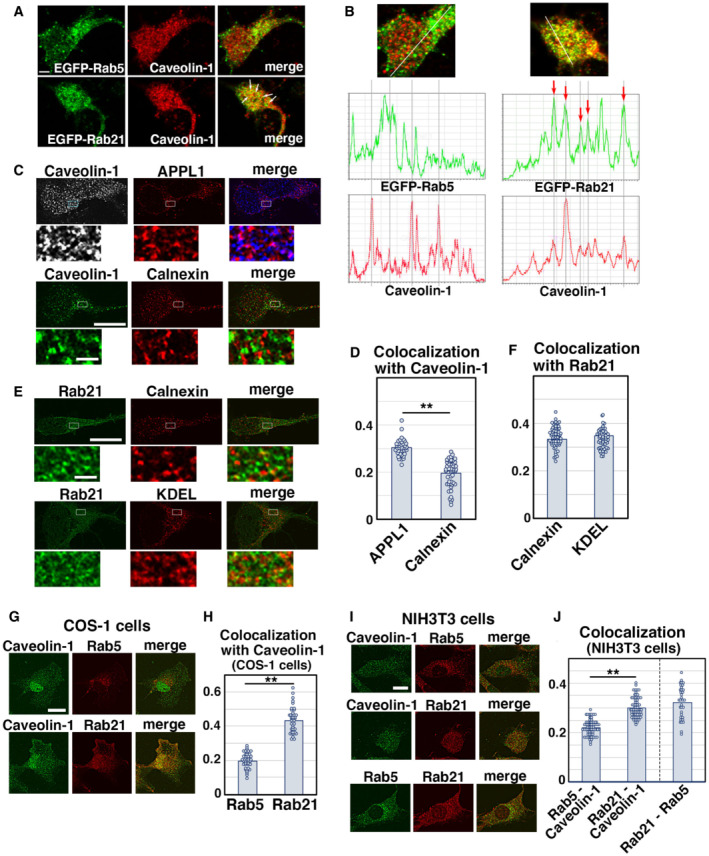
Subcellular localization of Rab21 and caveolin‐1 in primary cortical neurons and nonneuronal cultured cells A, BPrimary cortical neurons from E15 cerebral cortices were transfected with EGFP‐Rab5 (upper panels in A) or EGFP‐Rab21 (lower panels in A) and incubated for 2 days *in vitro*. To maintain moderate expression levels, CMV promoter was used to express EGFP‐Rab5 and EGFP‐Rab21. Cells were immunostained with anti‐EGFP (green) and anti‐caveolin‐1 (red) antibodies. White arrows in (A) indicate colocalization of EGFP‐Rab21 and caveolin‐1. The images are obtained with TCS‐SP5 (Leica). The graphs in (B) show the estimation of colocalization, which was carried out by recording fluorescence intensities of EGFP‐Rab5 or EGFP‐Rab21 and caveolin‐1 staining signals along the white line in the upper panels using Leica SP5 software. Red arrows indicate the colocalization of these proteins on the same peaks.C–FPrimary cortical neurons from E15 cerebral cortices were transfected with the indicated plasmids, incubated for 2 days *in vitro* and immunostained with the indicated antibodies. Lower panels in (C) and (E) are high magnification images indicated by white and blue rectangles in upper panels. Blue alone channels are shown in black and white images. The graphs in (D) and (F) show the Pearson's correlation coefficient between caveolin‐1 (D) or Rab21 (F) and organelle markers. Each score represents the mean with the individual points. Caveolin‐1—APPL1: *n* = 38 cells, Caveolin‐1—calnexin: *n* = 51 cells, Rab21—calnexin: *n* = 59 cells, Rab21—KDEL: *n* = 51 cells.G–JNIH3T3 or COS‐1 cells were immunostained with the indicated antibodies. The images are obtained with high‐resolution microscopy (Nikon). The graphs in (H) and (J) show the Pearson's correlation coefficient between caveolin‐1 and Rab5 or Rab21 or between Rab21 and Rab5. Each score represents the mean with the individual points. Caveolin‐1—Rab21: *n* = 41 cells (H) or 63 cells (J), Caveolin‐1—Rab5: *n* = 63 cells (H) or 69 cells (J), Rab21—Rab5: 34 cells. Data information: (D) Significance was determined by Mann–Whitney's *U* test (*P* = 1.310E‐14, **P < 0.01). (F) Significance was determined by one‐way ANOVA with *post hoc* Tukey–Kramer. No significant difference was observed between Rab21—Calnexin and Rab21—KDEL, but compared to a negative control (Rab5—Lamp1 in Fig 1B), a significant difference was observed (less than the critical value at 1%: Rab21—calnexin, Rab21—KDEL [compared to a negative control]). (H, J) Significance between Caveolin‐1—Rab21 and Caveolin‐1—Rab5 was determined by Welch's t‐test (H: *P* = 5.795E‐27, J: *P* = 7.367E‐24). **P < 0.01. Scale bar: 3 μm in (A), 10 μm in (upper panels in C, E), 1 μm in (lower panels in C, E), 5 μm in (G, I).

As previous reports suggested that Rab5 interacts with caveolin‐1 and regulates caveolin‐mediated endocytic pathways (Pelkmans *et al*, 2004; Hagiwara *et al*, 2009; Hayer *et al*, 2010; Ariotti & Parton, 2013), we examined the colocalization between Rab5 and caveolin‐1 in cortical neurons. In contrast to previous findings, however, we found that EGFP‐fused Rab5 driven by a CMV promoter (EGFP‐Rab5), as well as endogenous Rab5, showed little overlap with caveolin‐1 in primary cortical neurons (Figs 3A and C and EV3A and B). However, we observed that CMV promoter‐driven EGFP‐fused Rab21 (EGFP‐Rab21) co‐localizes with caveolin‐1 in primary cortical neurons (Fig EV3A and B). Endogenous Rab21 and caveolin‐1 were found to be co‐localized near the plasma membrane (as visualized with transfected plasma membrane‐targeted monomeric Azami‐Green 1 [PM‐mAG1]; Fig 3B). The Pearson's correlation coefficient between PM‐mAG1 and Rab21 or caveolin‐1 is 0.528 and 0.405, respectively, and the Pearson's correlation coefficient between Rab21 and caveolin‐1 was higher than that of Rab5 and caveolin‐1 in whole cells (Rab21: 0.660, Rab5: 0.264) and at the plasma membrane (Rab21: 0.620, Rab5: 0.203) (Fig 3C). These data indicate that Rab21 preferentially co‐localizes with caveolin‐1 at the plasma membrane.

The colocalization of Rab21 and caveolin‐1 was also observed in the vesicular components (Fig 3B lower panels). Because both Rab21 and caveolin‐1 were localized at the early endosomes (Figs EV1B and C and EV3C and D), the vesicular components near the plasma membrane may be early endosomes. We observed some localization of Rab21 in the ER, which was visualized with anti‐calnexin or anti‐KDEL antibodies (Fig EV3E and F), as previously reported (Opdam *et al*, 2000). However, caveolin‐1 was rarely observed in the ER (Fig EV3C and D), suggesting that cooperation between Rab21 and caveolin‐1 does not occur in the ER.

We next analyzed immature neurons *in vivo* and found that Rab21 and caveolin‐1 were co‐localized in the immature neurons in the intermediate zone at E17 (Fig 3E and G). High‐resolution microscopy analyses revealed that Rab21 and caveolin‐1 were sometimes present on the same vesicular compartments in the immature neurons (Fig 3F). By contrast, Rab5 and caveolin‐1 were barely co‐localized in the immature neurons *in vivo* (Fig 3D and G).

To examine whether the colocalization of Rab21 and caveolin‐1 is a neuron‐specific phenomenon or not, we analyzed the localization of Rab21, Rab5, and caveolin‐1 in nonneuronal cells, such as HeLa, COS‐1, and NIH3T3 cells. We found that colocalization between Rab5 and caveolin‐1 was significantly lower than that of Rab21 and caveolin‐1 in HeLa cells (Fig 3H and I). The Pearson's correlation coefficient between Rab5 and Rab21 or caveolin‐1 was 0.339 and 0.546, respectively. A similar tendency was also observed in COS‐1 and NIH3T3 cells, but the colocalization of Rab21 and caveolin‐1 was lower in COS‐1 and NIH3T3 cells (Fig EV3G–J). These data suggest that Rab21 also co‐localizes with caveolin‐1 in some nonneuronal cells, as well as cortical neurons.

### Rab21 is involved in caveolin‐mediated endocytic pathways

**Figure 4:**
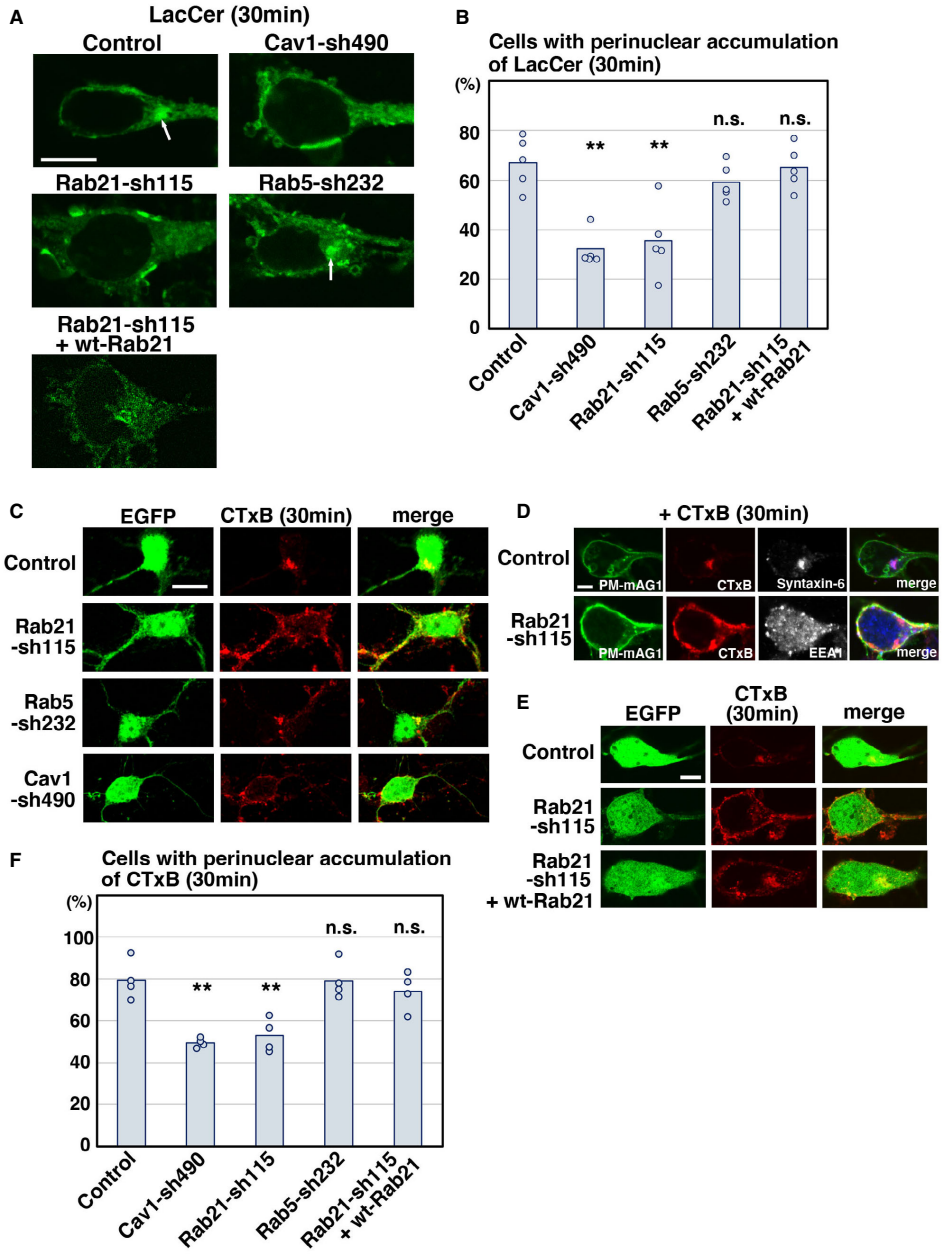
Rab21, but not Rab5, regulates caveolin‐1‐mediated trafficking pathways A–FPrimary cortical neurons from E15 cerebral cortices were transfected with the indicated plasmids plus pCAG‐EGFP (C and E) or pCAG‐PM‐mAG1 (D), incubated for 2 days *in vitro* and treated with BODIPY‐LacCer (LacCer) (green) (A, B) or Alexa555‐conjugated Cholera Toxin Subunit B (CTxB) (red) (C–F) for 30 min before fixation. White arrows in (A) indicate the perinuclear accumulation of LacCer. Blue alone channels are shown in black and white images. The graphs in (B) and (F) show the ratio of cells with perinuclear accumulation of LacCer (B) or CTxB (F), which was quantified in a blinded counting. Each score represents the mean with the individual points. (B) *n* = 5 biological replicates (Control: 115 cells, Cav1‐sh490: 127 cells, Rab21‐sh115: 136 cells, Rab5‐sh232: 216 cells, Rab21‐sh115 + wt‐Rab21: 132 cells), (F) *n* = 4 biological replicates (Control: 64 cells, Cav1‐sh490: 128 cells, Rab21‐sh115: 130 cells, Rab5‐sh232: 56 cells, Rab21‐sh115 + wt‐Rab21: 58 cells). Data information: (B and F) Significance compared to control was determined by one‐way ANOVA with *post hoc* Dunnett and Tukey–Kramer. **Less than the critical value at 1%, n.s.: no significant differences. Scale bars: 3 μm in (A), 10 μm in (C), 2 μm in (D), 5 μm in (E).

We next examined whether Rab21 and Rab5 are involved in caveolin‐1‐mediated endocytic pathways. To test this, we used BODIPY‐lactosylceramide (LacCer) and Alexa555‐Cholera Toxin Subunit B (CTxB) uptake assays, because LacCer and CTxB are internalized through the caveolin‐mediated endocytosis at least in part (Singh *et al*, 2003; Pelkmans & Zerial, 2005; Allen *et al*, 2011; Shvets *et al*, 2015). In the control neurons, LacCer and CTxB were internalized and transported to the perinuclear region within 30 min after treatment (Fig 4A–C and F). The internalized CTxB was found to be co‐localized with syntaxin‐6, a marker for *trans*‐Golgi network (Fig 4D). By contrast, LacCer or CTxB was still observed near the plasma membrane in the Rab21‐sh115‐transfected neurons 30 min after treatment (Fig 4A–D and F). These phenotypes could be rescued by the co‐expression of wt‐Rab21 (Fig 4A, B, E and F). Importantly, the ratio of cells with perinuclear accumulation of LacCer or CTxB is similar between Rab21‐ and caveolin‐1‐knockdown neurons (Fig 4B and F). These data indicate that Rab21 preferentially controls caveolin‐mediated endocytic pathways.

On the contrary, unlike the Tf uptake assay (Fig 1C and D), expression of Rab5‐sh232 did not significantly affect the internalization and intracellular trafficking of LacCer or CTxB (Fig 4A–C and F), indicating that Rab5 is required for the clathrin‐mediated, but not caveolin‐mediated, endocytic pathways. Thus, our results indicate that Rab21 and Rab5 regulate distinct endocytic pathways in neurons.

**Figure EV4:**
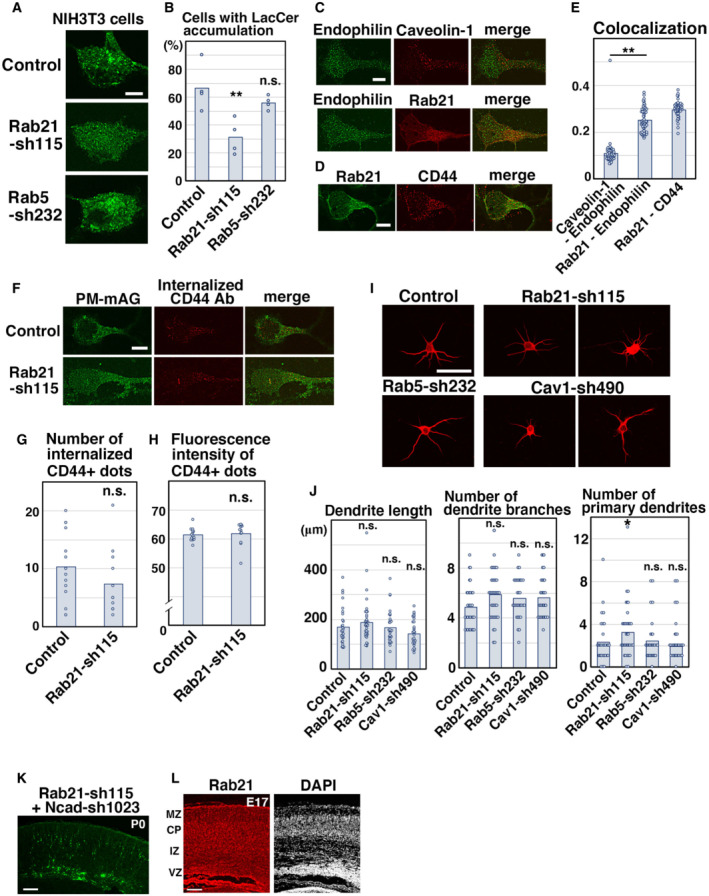
Rab21 is required for the uptake of LacCer, but not CD44 internalization A, BNIH3T3 fibroblasts were transfected with the indicated plasmids and treated with BODIPY‐LacCer (LacCer) for 30 min before fixation. The images are obtained with high‐resolution microscopy (Nikon). The graph in (B) shows the ratio of cells with perinuclear accumulation of LacCer. Each score represents the mean with the individual points. Control: *n* = 4 biological replicates, Rab21‐sh115: *n* = 4 biological replicates, Rab5‐sh232: *n* = 4 biological replicates.C–EPrimary cortical neurons from E15 cerebral cortices were incubated for 2 days *in vitro* and immunostained with the indicated antibodies. The images are obtained with high‐resolution microscopy (Nikon). The graph in (E) shows the Pearson's correlation coefficient between Endophilin and Rab21 or caveolin‐1 or between Rab21 and CD44. Each score represents the mean with the individual points. Caveolin‐1—Endophilin: *n* = 43 cells, Rab21—Endophilin: *n* = 53 cells, Rab21—CD44: *n* = 45 cells.F–HPrimary cortical neurons from E15 cerebral cortices were transfected with the indicated plasmids plus pCAG‐PM‐mAG1, incubated for 2 days *in vitro* and subjected to CD44 antibody feeding assay. The graphs in (G) and (H) show the number of the internalized CD44‐positive dots and its total fluorescence intensity per cell. Each score represents the mean with the individual points. Control: *n* = 15 cells (G and H), Rab21‐sh115: *n* = 12 cells (G and H).I, JPrimary cortical neurons from E15 cerebral cortices were transfected with the indicated plasmids plus pCAG‐EGFP, incubated for 8 days *in vitro* and stained with MAP2ab, a marker for dendrites. The graphs in (J) show the dendrite length, dendrite branch number and the number of primary dendrites. Each score represents the mean with the individual points. Control: *n* = 31 cells, Rab21‐sh115: *n* = 38 cells, Rab5‐sh232: *n* = 31 cells, Cav1‐sh490: *n* = 31 cells.KCerebral cortex at P0, electroporated with the indicated plasmids plus pCAG‐EGFP at E14.LFrozen sections of E17 cerebral cortex immunostained with anti‐Rab21 antibody and DAPI. Data information: (B) Significance was determined by one‐way ANOVA with *post hoc* Dunnett and Tukey–Kramer. **Less than the critical value at 1%, *less than the critical value at 5%, n.s.: no significant differences. (E) Significance was determined by one‐way ANOVA with *post hoc* Tukey–Kramer. **Less than the critical value at 1%. (G, H) Significance was determined by Welch's *t*‐test (G: *P* = 0.1942) or Mann–Whitney's *U* test (H: *P* = 0.2225). n.s.: no significant differences. (J) Significance compared to control was determined by one‐way ANOVA with *post hoc* Dunnett. Significant difference was observed between control and Rab21‐sh115 in the number of primary dendrites. *Less than the critical value at 5%, n.s.: no significant differences. Scale bars: 5 μm in (A, C, D, F), 10 μm in (I), 150 μm in (K), 100 μm in (L).

To examine whether the relationship between Rab21 and caveolin‐1 is applicable to nonneuronal cells, we performed the LacCer uptake assay using NIH3T3 fibroblasts. First, we analyzed the localization of Rab21 and Rab5 in NIH3T3 cells. Some overlap between Rab21 and Rab5 was observed in NIH3T3 cells, as previously reported (Egami & Araki, 2009; Simpson *et al*, 2004; Fig EV3I and J). Although the colocalization between Rab21 and caveolin‐1 is not very high in NIH3T3 cells (Fig EV3I and J), knockdown of Rab21, but not Rab5, significantly decreased the uptake of LacCer (Fig EV4A and B). This suggests that Rab21 is a main regulator of caveolin‐mediated endocytosis in NIH3T3 cells, although a minor role for Rab5 cannot be excluded.

It has been reported that a caveolin‐1‐mediated endocytic pathway is associated with clathrin‐independent carriers/GPI‐AP enriched early endosomal compartment (CLIC/GEEC) endocytic pathway in nonneuronal cells (Chaudhary *et al*, 2014). CD44 is a marker for CLIC/GEEC endocytosis and Endophilin is required for membrane scission during clathrin‐independent endocytosis (Chaudhary *et al*, 2014; Boucrot *et al*, 2015; Renard *et al*, 2015; Renard & Boucrot, 2021). However, colocalization between Endophilin and Rab21 or caveolin‐1 was not very high in primary cortical neurons (Fig EV4C and E). Low‐level colocalization between Rab21 and CD44 occurred (Fig EV4D and E), but no significant differences in the uptake of anti‐CD44 antibody between control and Rab21‐knockdown neurons can be detected (Fig EV4F–H). In addition, the tubular localization of CD44, whose formation depends on Rab21 function in nonneuronal cells (Del Olmo *et al*, 2019), was barely observed in cortical neurons (Fig EV4D). These data suggest that the role and/or molecular machinery of the CLIC/GEEC endocytosis may differ between neurons and nonneuronal cells.

### Rab21 is partly involved in dendrite maturation *in vitro*

We next analyzed the involvement of Rab21, caveolin‐1, and Rab5 during the early phase of dendrite formation in primary cortical neurons. Primary cortical neurons from E15 cerebral cortices were incubated for 8 days *in vitro* and immunostained with microtubule‐associated protein 2ab (MAP2ab), a marker for dendrites. Rab21‐knockdown slightly but significantly increased the number of primary dendrites, which may resemble the *in vivo* phenotypes of Rab21‐sh115‐electroporated neurons (Figs EV4I and 4J). By contrast, no differences were observed in the dendrite length and branch number between control and Rab21‐ or caveolin‐1‐ or Rab5‐knockdown neurons (Fig EV4I and J). Although immature neurite pruning, the early step of neuronal maturation, is only observed *in vivo*, primary cortical neurons may mimic the molecular machinery of *in vivo* neuronal maturation at least in part.

### Rab21 is required for N‐cadherin trafficking in immature neurons

**Figure 5:**
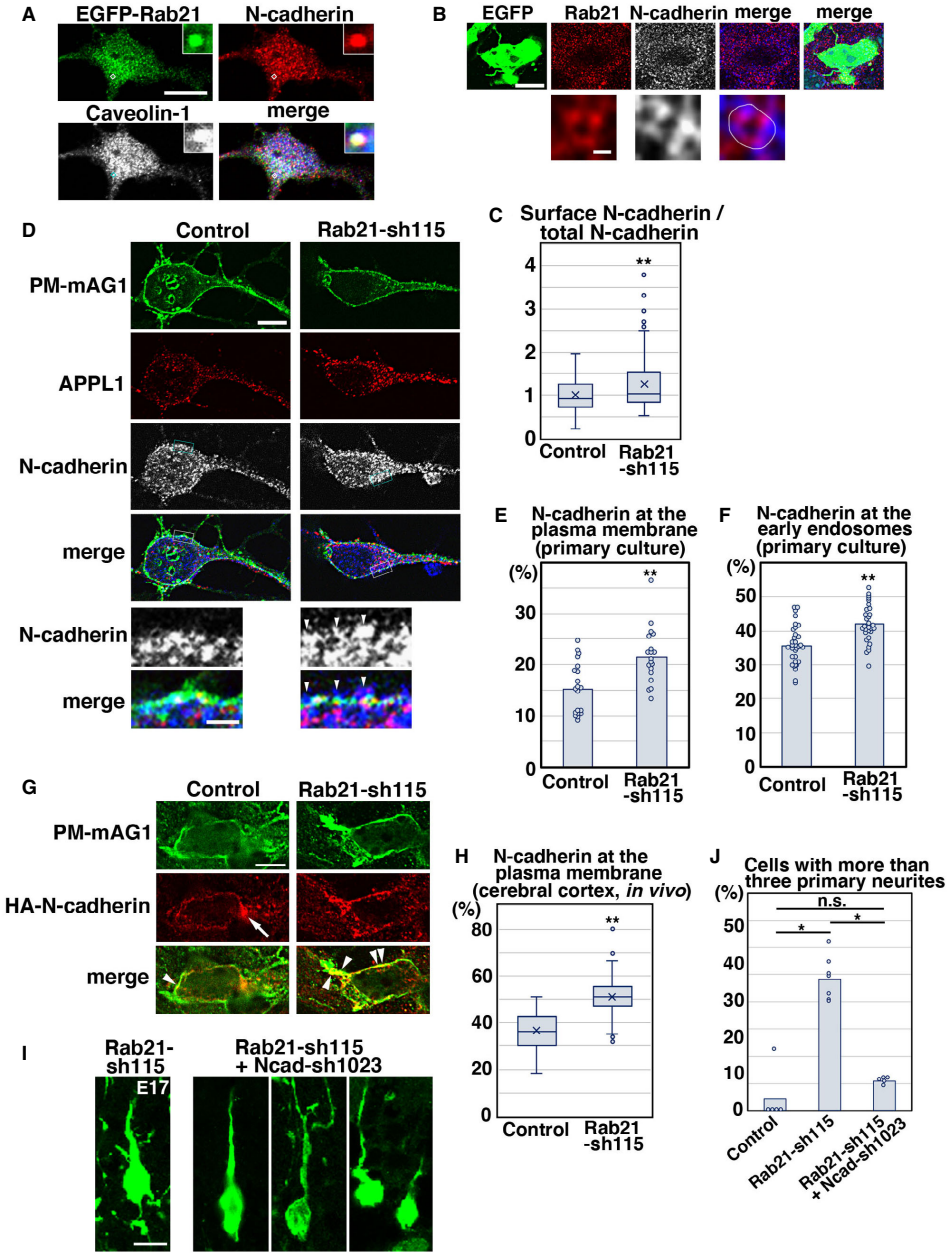
Rab21 promotes the internalization and early endosomal trafficking of N‐cadherin APrimary cortical neurons from E15 cerebral cortices transfected with pCMV‐EGFP‐Rab21, incubated for 2 days *in vitro* and stained with the indicated antibodies. Blue alone channels are shown in black and white images. Insets are high magnification images indicated by white or blue rectangles.BImmature neurons in the IZ of the cerebral cortices at E17, electroporated with pCAG‐EGFP at E14. Frozen sections were immunostained with the indicated antibodies. The images are obtained with high‐resolution microscopy (Nikon). Blue alone channels are shown in black and white images. The lower panels are high magnification images. Rab21 and N‐cadherin were sometimes present on the same vesicular compartments.CThe box‐and‐whisker plot shows the ratio of cell surface to total N‐cadherin in primary cortical neurons (2 days *in vitro*). Control: *n* = 85 cells from 3 biological replicates, Rab21‐sh115: *n* = 143 cells from three biological replicates.D–FPrimary cortical neurons from E15 cerebral cortices were transfected with pCAG‐PM‐mAG1, incubated for 2 days *in vitro* and stained with the indicated antibodies. Blue alone channels are shown in black and white images. Arrowheads indicate accumulation of N‐cadherin at the plasma membrane. Lower panels in (D) are high magnification images indicated by white or blue rectangles. The images are obtained with high‐resolution microscopy (Nikon). The graphs show the ratios of the N‐cadherin staining signals in the plasma membrane (E) and the APPL1‐positive early endosomes (F) to total fluorescence intensities of N‐cadherin in each primary cortical neuron. Each score represents the mean of ratios with the individual points. Control: *n* = 20 cells (E) or 33 cells (F), Rab21‐sh115: *n* = 19 cells (E) or 30 cells (F).G, HImmature neurons in the IZ of the cerebral cortices at E17, electroporated with the indicated plasmids plus pCAG‐PM‐mAG1 and pCAG‐HA‐N‐cadherin at E14. Frozen sections were immunostained with anti‐mAG1 and anti‐HA antibodies. Arrow and arrowheads indicate the accumulation of HA‐N‐cadherin at the perinuclear regions or the plasma membrane, respectively. The box‐and‐whisker plot shows the ratio of the HA‐N‐cadherin staining signals in the plasma membrane to total fluorescence intensities of HA‐N‐cadherin in each immature neuron *in vivo*. Control: *n* = 77 cells from three biological replicates, Rab21‐sh115: *n* = 108 cells from three biological replicates.ILocomoting neurons in the upper IZ of the cerebral cortices at E17, electroporated with the indicated plasmids plus pCAG‐EGFP at E14.JThe ratio of locomoting neurons with more than three primary neurites in the IZ. Each score represents the mean of ratios with the individual points. Control: *n* = 5 brains, Rab21‐sh115: *n* = 7 brains, Rab21‐sh115 + Ncad‐sh1023: *n* = 5 brains. Data information: (C, H) In the box‐and‐whisker plots, the central band and the upper and lower sides of the boxes indicate the median and the upper and lower quartiles. The whiskers of the depicted boxplots go from the minimum to the lower quartile and from the upper quartile to the maximum. “x” indicates the average value. In (C) significance compared to control was determined by Welch's *t*‐test (*P* = 0.0003106). **P < 0.01. In (H) significance compared to control was determined by Student's *t*‐test (*P* = 3.951E‐28). **P < 0.01. (E, F) Significance compared to control was determined by Student's *t*‐test (E: *P* = 0.0005588, F: *P* = 0.00003682). **P < 0.01. (J) Significance was determined by Kruskal–Wallis test with *post hoc* Steel–Dwass test. *Less than the critical value at 5%. Scale bars: 10 μm in (A), 4 μm in (upper panels in B), 0.2 μm in (lower panels in B), 5 μm in (D), 1 μm in (lower panels in D), 4 μm in (G), 10 μm in (I).

Given that Rab21 is involved in caveolin‐mediated endocytic pathways, we examined whether Rab21 controls N‐cadherin trafficking, because our previous study showed that caveolin‐1 regulates N‐cadherin internalization to promote the immature neurite pruning of cortical neurons (Shikanai *et al*, 2018). We found that EGFP‐Rab21, N‐cadherin, and caveolin‐1 are partially co‐localized in primary cortical neurons (Fig 5A). Endogenous Rab21 is also co‐localized with N‐cadherin in primary cortical neurons and immature neurons *in vivo* (Fig 5B and Appendix Fig S1A and B).

To examine whether Rab21 regulates N‐cadherin endocytosis, we analyzed the cell surface levels of N‐cadherin in control and Rab21‐knockdown neurons. The neurons were fixed before permeabilization and treated with antibody recognizing the extracellular region of N‐cadherin to visualize cell surface N‐cadherin. Subsequently, the neurons were permeabilized with Triton X‐100 and treated with another anti‐N‐cadherin antibody that detects total N‐cadherin. The ratio of cell surface N‐cadherin to total N‐cadherin was significantly increased in the Rab21‐knockdown neurons, compared with control (Fig 5C).

Following N‐cadherin endocytosis, the internalized N‐cadherin is transported to the early endosomes, and subsequently to the Rab11‐positive recycling endosomes or the Rab7‐positive late endosomes to lesser extent (Appendix Fig S1A–C; Kawauchi *et al*, 2010). When we quantified the N‐cadherin staining signals in the plasma membrane (PM‐mAG1‐positive regions) and early endosomes (APPL1‐positive regions), increased N‐cadherin staining signals were observed at both the plasma membrane and early endosomes of the Rab21‐sh115‐transfected neurons (Fig 5D–F). These data suggest that Rab21 regulates both N‐cadherin internalization from the plasma membrane and its trafficking in early endosomes.

To analyze the cell surface levels of N‐cadherin *in vivo*, HA‐tagged N‐cadherin and PM‐mAG1 expressing vectors were electroporated into E14 cerebral cortices and N‐cadherin localization was quantified. The ratio of the cell surface N‐cadherin in control immature neurons *in vivo* was higher than that *in vitro*, possibly because cell‐to‐cell adhesion is not much observed in primary cortical neurons *in vitro*. Importantly, colocalization of HA‐tagged N‐cadherin and PM‐mAG1 was further increased in the Rab21‐sh115‐electroporated immature neurons (Fig 5G and H), suggesting that Rab21 regulates N‐cadherin endocytosis in the immature neurons *in vivo*.

We next analyzed whether Rab21‐mediated internalization of N‐cadherin is required for immature neurite pruning. Rab21‐sh115 and a low concentration of the N‐cadherin knockdown vector (Ncad‐sh1023; Kawauchi *et al*, 2010) were co‐electroporated into E14 cerebral cortices and the electroporated brains were fixed at E17, 3 days after electroporation. While Rab21‐sh115‐electroporated locomoting neurons abnormally extended many immature neurites as described above, co‐electroporation with Rab21‐sh115 and Ncad‐sh1023, which were expected to restore the increased cell surface levels of N‐cadherin, rescued the pruning defects in immature neurites (Fig 5I and J), similar to the case of caveolin‐1 knockdown (Shikanai *et al*, 2018). However, some neurons that were co‐transfected with Rab21‐sh115 and Ncad‐sh1023 exhibited a leading process with abnormal morphology, likely due to the requirement of N‐cadherin for the leading process elongation (Shikanai *et al*, 2018). These data suggest that Rab21‐ and caveolin‐1‐mediated immature neurite pruning depends on N‐cadherin internalization.

By contrast, Ncad‐sh1023 could not rescue the migration defects elicited by Rab21‐sh115 (Fig EV4K), although our previous study indicated that a low concentration of Ncad‐sh1023 efficiently restores the migration defects in the caveolin‐1 knockdown neurons (Shikanai *et al*, 2018). It suggests an additional role for Rab21 in migrating neurons. In fact, Rab21 was expressed throughout the cortex (Fig EV4L) and regulates both endocytosis and early endosome‐mediated trafficking of N‐cadherin (Fig 5D–F), whereas caveolin‐1 is expressed in the intermediate zone, but not the cortical plate, of the cortex and only required for the internalization of N‐cadherin from the plasma membrane (Shikanai *et al*, 2018).

### Rab21 maintains the plasma membrane localization and protein levels of caveolin‐1

**Figure 6:**
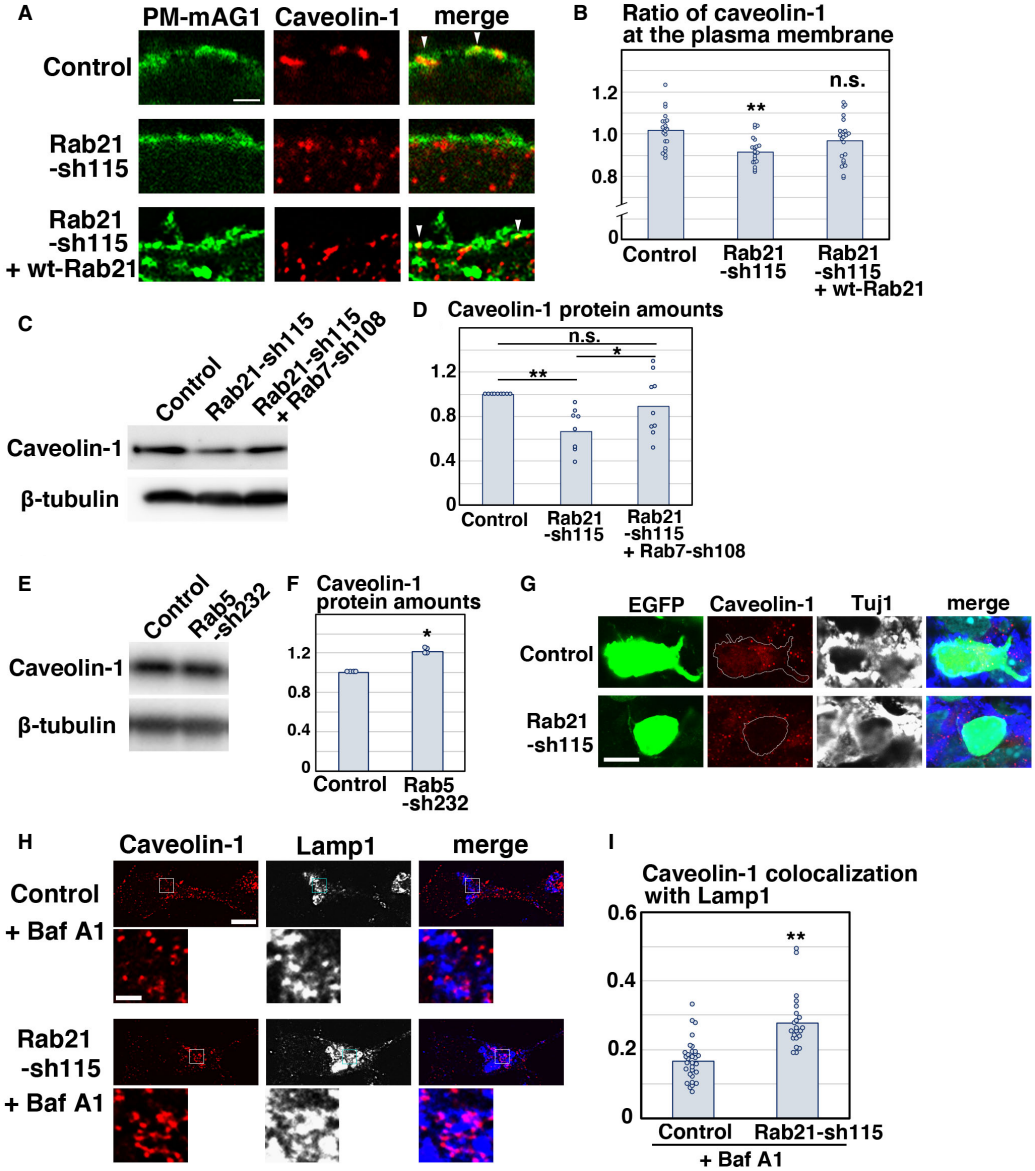
Rab21 regulates caveolin‐1 membrane localization and protein levels A, BPrimary cortical neurons from E15 cerebral cortices were transfected with the indicated plasmids plus pCAG‐PM‐mAG1 (green) and incubated for 2 days *in vitro*. Immunocytochemical analyses with anti‐caveolin‐1 (red) antibody were performed. The images are obtained with high‐resolution microscopy (Nikon). The graph in (B) indicates the ratio of the caveolin‐1 staining signals in the plasma membrane to total fluorescence intensities of caveolin‐1 in each immature neuron. Each score represents the mean of ratios with the individual points. Control: *n* = 20 cells, Rab21‐sh115: *n* = 22 cells, Rab21‐sh115 + pCMV‐wt‐Rab21: *n* = 23 cells. Overexpression of wt‐Rab21 resulted in neuronal cell death, which making it difficult to determine the accurate DNA concentration of pCMV‐wt‐Rab21 in the rescue experiments.C–FPrimary cortical neurons from E15 cerebral cortices were transfected with the indicated plasmids and incubated for 2 days *in vitro*. Immunoblot analyses of cell lysates with the indicated antibodies were performed. The graphs in (D) and (F) indicate the mean ratios of immunoblot band intensities of caveolin‐1/β‐tubulin with the individual points.GImmature neurons in the IZ of the cerebral cortices at E17, electroporated with the indicated plasmids plus pCAG‐EGFP at E14. Frozen sections were immunostained with the indicated antibodies. Blue alone channels are shown in black and white images.H, IPrimary cortical neurons from E15 cerebral cortices were transfected with the indicated plasmids, incubated for 2 days *in vitro*, treated with Bafilomycin A1 (Baf A1) for 6 h and stained with the indicated antibodies. The images are obtained with high‐resolution microscopy (Nikon) and the lower panels are high magnification images of the lysosomes, indicated by white or blue rectangles. Blue alone channels are shown in black and white images. The graph in (I) shows the Pearson's correlation coefficient between caveolin‐1 and Lamp1, a lysosomal marker, in control and Rab21‐sh115‐transfected neurons. Each score represents the mean of ratios with the individual points. Control: *n* = 31 cells, Rab21‐sh115: *n* = 22 cells. Data information: (B) Significance was determined by one‐way ANOVA with *post hoc* Dunnett and Tukey–Kramer. **Less than the critical value at 1%. Significant difference was observed between control and Rab21‐sh115, but not between control and the co‐expression of Rab21‐sh115 and pCMV‐wt‐Rab21, by Dunnett and Tukey–Kramer. However, no significance was observed between Rab21‐sh115 alone and co‐expression of Rab21‐sh115 and pCMV‐wt‐Rab21 by Tukey–Kramer. (D and F) Significance was determined by one‐way ANOVA with *post hoc* Tukey–Kramer (D: *n* = 9 biological replicates) and Mann–Whitney's *U* test (F: *P* = 0.02102, *n* = 4 biological replicates). n.s.: no significant differences, *less than the critical value at 5%, **less than the critical value at 1%. (I) Significance was determined by Mann–Whitney's *U* test (*P* = 0.0000006290). **P < 0.01. Scale bars: 1 μm in (A, lower panels in H), 4 μm in (G), 5 μm in (upper panels in H).

**Figure EV5:**
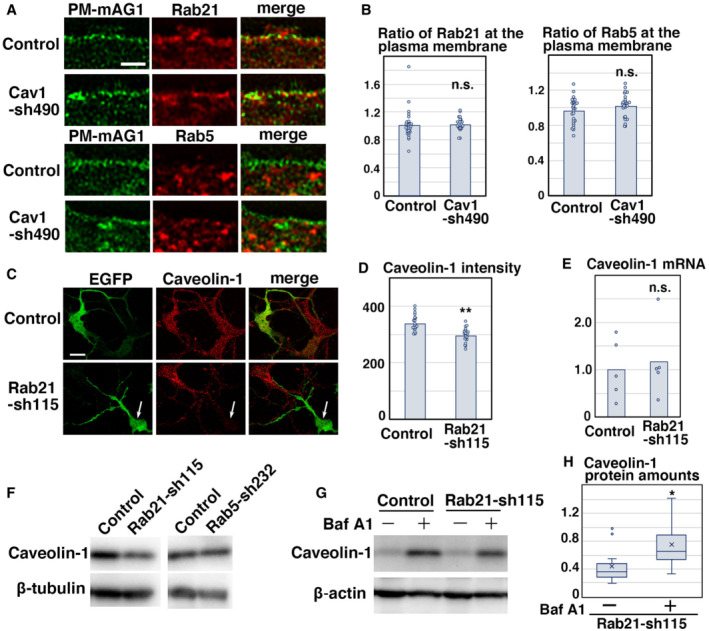
Knockdown of Rab21, but not Rab5, decrease the caveolin‐1 protein levels A, BPrimary cortical neurons from E15 cerebral cortices were transfected with the indicated plasmids plus pCAG‐PM‐mAG1 (green) and incubated for 2 days *in vitro*. Immunocytochemical analyses with anti‐Rab21 or anti‐Rab5 antibody (red) were performed. The graphs in (B) indicate the ratio of the Rab21 or Rab5 staining signals in the plasma membrane to total fluorescence intensities in each immature neuron. Each score represents the mean with the individual points. Control: *n* = 29 cells (left, Rab21) or 27 cells (right, Rab5), Cav1‐sh490: *n* = 24 cells (left, Rab21) or 25 cells (right, Rab5).C, DPrimary cortical neurons from E15 cerebral cortices transfected with the indicated plasmids plus pCAG‐EGFP and incubated for 2 days *in vitro*. Immunocytochemical analyses with anti‐EGFP (green) and anti‐caveolin‐1 (red) antibodies (C, D) and quantitative PCR (E) were performed. White arrows in (C) indicate the Rab21‐knockdown neurons with decreased caveolin‐1 signals. The graph in (D) shows the fluorescence intensity of caveolin‐1 per cell. Each score represents the mean with the individual points. Control: *n* = 21 cells, Rab21‐sh115: *n* = 22 cells. Each score in (E) represents the ratio with the individual points.FPrimary cortical neurons from E15 cerebral cortices transfected with the indicated plasmids and incubated for 5 days *in vitro* (DIV). Immunoblot analyses of cell lysates with the indicated antibodies were performed. Long‐term culture (5 DIV) did not affect the protein levels of caveolin‐1 in the Rab5‐sh232‐transfected cortical neurons, similar to the 2 DIV neurons (Fig 6E), whereas the reduced caveolin‐1 protein levels were still observed in the Rab21‐sh115‐transfected neurons at 5 DIV.G, HPrimary cortical neurons from E15 cerebral cortices transfected with the indicated plasmids, incubated for 1 DIV and treated with 160 nM Bafilomycin A1 (Baf A1) for 22 h. Immunoblot analyses of the cell lysates with the indicated antibodies were performed. The box‐and‐whisker plot in (H) shows the ratios of immunoblot band intensities of caveolin‐1/beta‐actin. Baf A1^−^: *n* = 11 biological replicates, Baf A1^+^: *n* = 10 biological replicates. Data information: (B) Significance was determined by Mann–Whitney's *U* test (Rab21: *P* = 0.2176) or Student's *t*‐test (Rab5: *P* = 0.1599), and no significant difference was observed. n.s.: no significant differences. (D) Significance was determined by Student's *t*‐test (*P* = 0.000004220). **P < 0.01. (E) Significance was determined by Welch's *t*‐test (*n* = 5 biological replicates, *P* = 0.7262). n.s.: no significant differences. (H) In the box‐and‐whisker plots, the central band and the upper and lower sides of the boxes indicate the median and the upper and lower quartiles. The whiskers of the depicted boxplots go from the minimum to the lower quartile and from the upper quartile to the maximum. “x” indicates the average value. Significance was determined by Welch's t‐test (*P* = 0.03234). *P < 0.05. Scale bar: 1 μm in (A), 10 μm in (C).

Given that Rab21 and caveolin‐1 regulate the same step in neuronal maturation, we examined the molecular relationship between Rab21 and caveolin‐1 in cortical neurons. We transfected primary cortical neurons with Rab21‐sh115 and analyzed the membrane localization of caveolin‐1 using co‐transfected PM‐mAG1. Knockdown of Rab21 results in the reduction in plasma membrane‐localized caveolin‐1. This phenotype was partially rescued by the co‐expression of wt‐Rab21; significant difference was observed between control and Rab21‐sh115, but not between control and the co‐expression of Rab21‐sh115 and wt‐Rab21 (Fig 6A and B). However, no significance was observed between Rab21‐sh115 alone and co‐expression of Rab21‐sh115 and wt‐Rab21. Overexpression of wt‐Rab21 resulted in neuronal cell death, which making it difficult to determine the accurate DNA concentration of wt‐Rab21 in the rescue experiments in primary cortical neurons. In contrast to Rab21 knockdown, knockdown of caveolin‐1 did not affect the membrane localization of Rab21 or Rab5 (Fig EV5A and B). These data indicate that Rab21 promotes the plasma membrane localization of caveolin‐1 as an upstream regulator.

Interestingly, we noticed that the caveolin‐1 staining was decreased in a portion of the Rab21‐sh115‐transfected neurons (Fig EV5C). The fluorescence intensities of caveolin‐1 in each cell were decreased in Rab21‐knockdown neurons (Fig EV5D). Immunoblot analyses indicated that caveolin‐1 proteins are significantly decreased at 2 DIV, although mRNA levels are not significantly different by quantitative PCR analyses (Figs 6C and D and EV5E). Immunohistochemical analyses confirmed the reduction in endogenous caveolin‐1 protein levels in the Rab21‐sh115‐transfected neurons *in vivo* (Fig 6G).

In contrast to Rab21 knockdown, Rab5‐sh232 did not decrease the protein levels of caveolin‐1 in cortical neurons at 2 DIV (Fig 6E and F). Rather, the caveolin‐1 protein was slightly increased, which may be consistent with previous reports showing that suppression of one endocytosis type enhances other endocytic pathways (Damke *et al*, 1995). Similar results were obtained when we analyzed the caveolin‐1 protein levels in Rab21‐sh115‐ or Rab5‐sh232‐transfected neurons at 5 DIV (Fig EV5F). These data suggest that Rab21, but not Rab5, is required for the membrane localization and maintaining the protein levels of caveolin‐1 in cortical neurons.

Caveolin‐1 is anchored to the membrane and its degradation occurs in the lysosomes (Hayer *et al*, 2010). Upon inhibition of the activities of lysosomal proteases by treatment of primary cortical neurons with Bafilomycin A1, a lysosomal inhibitor, caveolin‐1 staining signals were increased in the Lamp1‐positive lysosomes in Rab21‐knockdown neurons (Fig 6H and I). These data suggest that in Rab21‐knockdown neurons, where caveolin‐1 membrane localization is decreased, mislocalized caveolin‐1 is transported to the lysosomes and degraded, which may account for the reduction in total protein levels of caveolin‐1 in Rab21‐knockdown neurons.

To confirm this, we attempted to suppress the trafficking pathway to the lysosomes by transfection of a previously reported Rab7 knockdown vector (Rab7‐sh108; Kawauchi *et al*, 2010). Suppression of Rab7‐dependent lysosomal degradation pathways restored the reduced protein levels of caveolin‐1 in the Rab21‐sh115‐transfected neurons, suggesting that knockdown of Rab21 leads to lysosomal degradation of caveolin‐1 possibly due to missorting of caveolin‐1‐containing vesicles to lysosomes, instead of the plasma membrane (Fig 6C and D). Similar results were obtained with Bafilomycin A1 treatment, although Bafilomycin A1 dramatically increased caveolin‐1 protein levels even in control cells (Fig EV5G and H).

### Rab21‐mediated regulation of caveolin‐1 protein levels is required for neuronal maturation

**Figure 7:**
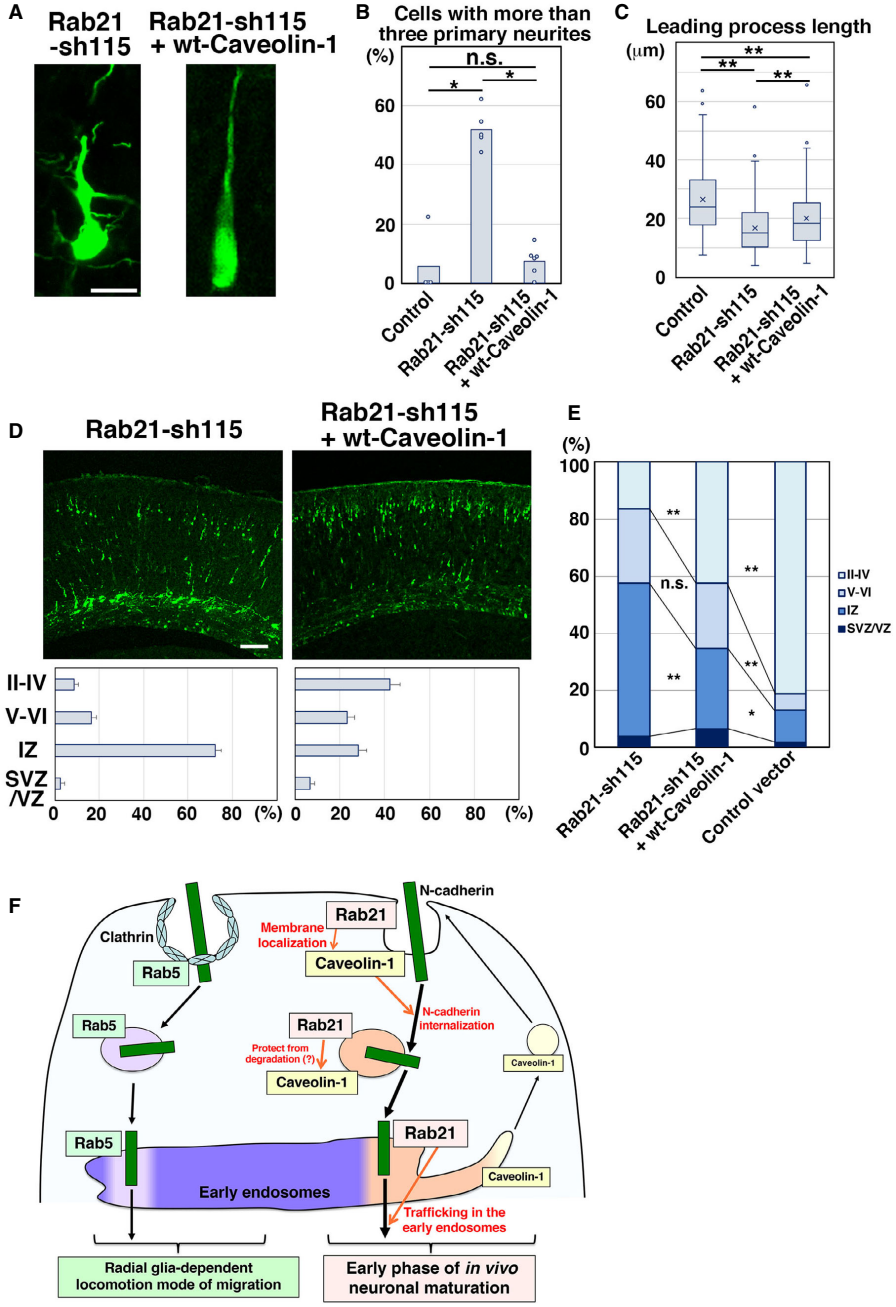
Rab21 regulates neuronal maturation through the maintenance of caveolin‐1 protein levels ALocomoting neurons in the upper IZ of the cerebral cortices at E17, electroporated with the indicated plasmids plus pCAG‐EGFP at E14.BThe ratio of locomoting neurons with more than three primary neurites. Each score represents the mean of ratios with the individual points. Control: *n* = 4 brains, Rab21‐sh115: *n* = 5 brains, Rab21‐sh115 + pCAG‐wt‐Caveolin‐1: *n* = 6 brains.CThe box‐and‐whisker plot shows average leading process length of the locomoting neurons in the IZ. Control: *n* = 115 cells, Rab21‐sh115: *n* = 188 cells, Rab21‐sh115 + Caveolin‐1: *n* = 185 cells.D, ECerebral cortices at P0, electroporated with the indicated plasmids plus pCAG‐EGFP at E14. The lower graphs in (D) and the graph in (E) show the estimation of cell migration, as measured by EGFP fluorescence intensities in distinct regions of the cerebral cortices using Leica SP5 software. Each bar in the graph in (E) represents the mean percentage of relative intensities. Rab21‐sh115: *n* = 4 brains, Rab21‐sh115 + pCAG‐wt‐Caveolin‐1: *n* = 6 brains. II‐IV: layers II‐IV of the cortical plate, V‐VI: layers V‐VI of the cortical plate, IZ: intermediate zone, WM: white matter, SVZ/VZ: subventricular zone/ventricular zone.FRab5 and Rab21, members of the same subfamily, mainly regulate distinct endocytic pathways, clathrin‐mediated and caveolin‐1‐mediated, respectively, and different steps of neuronal maturation and migration. While Rab5 is required for the locomotion mode of neuronal migration (Kawauchi *et al*, 2010), our present study indicates that Rab21 and caveolin‐1 cooperatively regulate immature neurite pruning and leading process elongation. Rab21 promotes the membrane localization of caveolin‐1 and maintains the caveolin‐1 protein levels, which is required for immature neurite pruning via the enhanced internalization of N‐cadherin. Rab21 also has caveolin‐1‐independent functions in the trafficking in early endosomes. Data information: (B) Significance was determined by Kruskal–Wallis test (*P* = 0.007458) with *post hoc* Steel–Dwass test. *Less than the critical value at 5% (compared with control), n.s.: no significant differences. (C) In the box‐and‐whisker plots, the central band and the upper and lower sides of the boxes indicate the median and the upper and lower quartiles. The whiskers of the depicted boxplots go from the minimum to the lower quartile and from the upper quartile to the maximum. “x” indicates the average value. Significance was determined by Kruskal–Wallis test (*P* = 2745E‐13) with *post hoc* Steel–Dwass test. **Less than the critical value at 1%. (E) Significance compared to Rab21‐sh115 was determined by Student's *t*‐test (Rab21‐sh115 + pCAG‐wt‐Caveolin‐1 [Layer II‐IV]: *P* = 0.003543, Rab21‐sh115 + pCAG‐wt‐Caveolin‐1 [IZ]: *P* = 0.003405) and one‐way ANOVA with *post hoc* Tukey–Kramer. **P < 0.01. n.s.: no significant differences, *less than the critical value at 5%, **less than the critical value at 1%. See Fig EV2B and C for the ratio of the number of the electroporated cells in each layer. Scale bars: 10 μm in (A), 100 μm in (D).

To determine whether Rab21‐mediated maintenance of caveolin‐1 protein levels is required for neuronal maturation, we performed rescue experiments. Rab21‐sh115 and pCAG‐wt‐caveolin‐1 were co‐electroporated into E14 mouse cerebral cortices, and the electroporated brains were fixed at E17 or P0. Co‐expression of wt‐caveolin‐1, which is expected to increase the overall cellular caveolin‐1 protein levels and possibly also in the plasma membrane, as a low‐level Rab21 expression remained in the knockdown cells, dramatically restored the immature neurite pruning in the Rab21 knockdown neurons (Fig 7A and B). Caveolin‐1 might also act as a scaffold protein to indirectly activate Rab21 because previous reports indicate that overexpression of caveolin‐1 activates other small GTPases (Grande‐Garcia *et al*, 2007; Diaz *et al*, 2014), although our results show that knockdown of caveolin‐1 does not affect the plasma membrane localization of Rab21 (Fig EV5A and B). Furthermore, the expression of wt‐caveolin‐1 partially rescued the shortened leading process length and neuronal positioning defects caused by Rab21 knockdown (Fig 7C–E). These data suggest that Rab21‐mediated regulation of caveolin‐1 plays a pivotal role in immature neurite pruning. This regulation is also required for proper neuronal positioning to some extent, although Rab21 may have an additional caveolin‐1‐independent function in the neuronal migration.

### Discussion

Almost all types of endocytosis, including clathrin‐ and caveolin‐mediated, lead to the early endosomes where the internalized transmembrane proteins are sorted to many subcellular compartments (therefore, early endosomes are also referred as to sorting endosomes). Thus, the early endosomes are thought of as a converging point of the endocytic pathways, and it was believed that Rab5 is a key regulator for both clathrin‐ and caveolin‐mediated endocytic pathways. In this study, however, we show that Rab5 and Rab21 have distinct roles in the regulation of endocytic trafficking in cortical neurons and in cerebral cortical development *in vivo* (Fig 7F). Our present data indicate that Rab5 and Rab21 are localized in the distinct populations of the early endosomes and preferentially regulate clathrin‐ and caveolin‐mediated endocytic pathways, respectively, suggesting that clathrin‐ and caveolin‐mediated endocytic pathways run in parallel, rather than converge in the early endosomes. Our observations are consistent with previous reports revealing that EEA1 and APPL1, markers for the early endosomes, are differentially localized on the early endosomal membrane *in vitro* (Kalaidzidis *et al*, 2015). However, another report also indicates that the early endosomes consist of several subdomains (Rink *et al*, 2005; Franke *et al*, 2019). Future studies are important to clarify whether Rab21‐ and Rab5‐positive compartments are located in different subdomains of the early endosomes or in distinct endosomes.

Our previous study indicated that *in vivo* knockdown of Rab5 disturbs the radial fiber‐dependent long‐distance migration of the leading process‐possessing locomoting neurons and suppresses the immature neurite formation (Kawauchi *et al*, 2010). The present study shows that Rab21, but not Rab5, is required for the pruning of the immature neurites during the multipolar‐to‐bipolar transition. Interestingly, a major cargo molecule of both Rab5 and Rab21 to promote neuronal migration and maturation, respectively, is N‐cadherin. This suggests that the precise spatiotemporal regulation of N‐cadherin endocytic trafficking is crucial for multiple steps of cortical development; Rab5‐ and Rab21‐mediated regulation of N‐cadherin is required for the radial fiber‐dependent neuronal migration and immature neurite pruning, respectively, which are distinct steps of neuronal maturation in the developing cerebral cortex.

At subcellular levels, clathrin‐mediated endocytosis occurs in the nonraft membrane domain, whereas caveolin‐1 is mainly localized in the cholesterol‐ and ganglioside‐rich detergent‐insoluble membrane domain (Parton, 1994; Nakashima *et al*, 2007). Consistently, caveolin‐1 is co‐localized with GD3 ganglioside, but not CD71, a nonraft marker (Macdonald & Pike, 2005; Mayle *et al*, 2012), in cortical neurons (Shikanai *et al*, 2018). Because our previous report indicates that N‐cadherin is localized in both GD3‐positive and GD3‐negative membrane domains (Shikanai *et al*, 2018), Rab5 and Rab21 may regulate different populations of N‐cadherin on the specific plasma membrane domains.

An important question raised by this study is how Rab5 and Rab21 control different trafficking pathways. A previous study using APEX2‐mediated proximity labeling of Rab5 and Rab21 indicates that the interaction between Rab21 and APPL1, an effector for Rab5 and Rab21, is slightly stronger than that between Rab5 and APPL1 (Del Olmo *et al*, 2019). In addition, several, but not all, guanine nucleotide exchange factors (GEFs) and GTPase‐activating proteins (GAPs), activators and suppressors for Rab proteins, respectively, exhibit a preference of binding to either Rab5 or Rab21, which may explain the role‐allocation of these proteins at the molecular level (Mori & Fukuda, 2013; Del Olmo *et al*, 2019).

Our study reveals the functional association between Rab21 and caveolin‐1. We show that Rab21 and caveolin‐1 are highly co‐localized at the plasma membrane and that knockdown of Rab21 decreases the membrane localization of caveolin‐1. The mislocalized caveolin‐1 in Rab21‐knockdown neurons is transported to the lysosomes and degraded. As a result, Rab21 knockdown results in the reduction in caveolin‐1 protein levels. Importantly, caveolin‐1 expression can rescue the defects in the immature neurite pruning of the Rab21‐knockdown immature neurons *in vivo*. By contrast, knockdown of caveolin‐1 does not affect the membrane localization of Rab21 and Rab5. These findings strongly indicate that Rab21 is an upstream regulator for caveolin‐1. Although the interlinked mechanisms of Rab21 and caveolin‐1 will be addressed in future studies, we speculate that Rab21 may directly or indirectly promote the proper localization and/or oligomerization of caveolin‐1 because defects in these processes appear to result in the lysosomal degradation of caveolin‐1 (Hayer *et al*, 2010; Burana *et al*, 2016).

## Materials and Methods

### Antibodies and chemical regents

Primary antibodies used in this study were anti‐Rab21 (R4405, Sigma), anti‐Rab5 (3547, Cell Signaling Technology; 46449, Cell Signaling Technology), anti‐APPL1 (3858, Cell Signaling Technology), anti‐EEA1 (07‐292, Upstate; 610456, BD Biosciences), anti‐caveolin‐1 (3238, Cell Signaling Technology for staining and immunoblotting; 610406, BD Biosciences for staining; 3267, Cell Signaling Technology for immunoblotting), anti‐Rab11 (5589, Cell Signaling Technology), anti‐Rab7 (9367, Cell Signaling Technology), anti‐Rab6 (9625, Cell Signaling Technology), anti‐Lamp1 (sc‐19992, Santa Cruz), anti‐Syntaxin‐6 (610635, BD Biosciences), anti‐Calnexin (610523, BD Biosciences), anti‐KDEL (sc‐58774, Santa Cruz), anti‐TfR (13‐6800, Invitrogen), anti‐CD44 (NB100‐65905, Novus), anti‐Endophilin‐II (sc‐365704, Santa Cruz), anti‐GFP (A‐6455, Molecular Probes; 04404‐84, Nacalai; AB16901, Millipore), anti‐mAG1 (PM052M, MBL), anti‐HA (2367, Cell Signaling Technology), anti‐N‐cadherin (C3865, Sigma; sc‐7939, Santa Cruz Biotechnology; ab98952, Abcam), anti‐Phospho Histone H3 (9701, Cell Signaling Technology), anti‐Ki67 (NCL‐Ki67p, Leica), anti‐βIII tubulin (Tuj1) (MMS‐435P, Covance), anti‐MAP2ab (ab11268, Abcam), anti‐β‐tubulin (T5201, Sigma), and anti‐β‐actin (A5441, Sigma). BODIPY‐FL C5‐LacCer (LacCer), Alexa555‐conjugated Cholera Toxin Subunit B (CTxB) and Alexa594‐ or Alexa555‐conjugated transferrin (Tf) were purchased from Molecular Probes. 4′,6‐diamidino‐2‐phenylindole dihydrochloride solution (DAPI), and Bafilomycin A1 were purchased from Wako (340‐07971) and Sigma (SML‐1661), respectively.

### Plasmids

Plasmids were prepared using the EndoFree plasmid purification kit (Qiagen). Human Rab21 cDNA was amplified from Marathon‐Ready human brain cDNA (BD Biosciences) by standard PCR techniques. EGFP‐Rab21 (human) cDNA was inserted into pCAG‐MCS2 and pTα1‐MCS1 to generate pCAG‐Rab21 and pTα1‐Rab21. pCAG‐EGFP, pCMV‐EGFP‐Rab21 (mouse), pCMV‐EGFP‐Rab5, Rab5‐sh232, pCAG‐wt‐Caveolin‐1, Cav1‐sh490, pCAG‐HA‐N‐cadherin, Ncad‐sh1023, Rab7‐sh108 and pCAG‐PM‐mAG1 were described previously (Kawauchi *et al*, 2003; Kawauchi *et al*, 2005; Kawauchi *et al*, 2010; Ohbayashi *et al*, 2012; Shikanai *et al*, 2018). pCMV‐Rab21 and pCMV‐Rab5 were generated from pCMV‐EGFP‐Rab21 (mouse) and pCMV‐EGFP‐Rab5, respectively.

To construct shRNA‐expressing vectors, oligonucleotides targeting the *Rab21* coding sequence (5’‐GAGAACAAGTTCAACGACA‐3′) and their complementary sequences were inserted into the pSilencer 3.1‐H1 vector (Ambion). All contain a hairpin loop sequence (5’‐TTCAAGAGA‐3′). These sequences were designed based on information from shRNA sequence analyses (B‐Bridge International, Inc). A control vector containing a scrambled nontargeting sequence was purchased from Ambion.

### *In utero* electroporation

Pregnant ICR mice were purchased from SLC Japan. Animals were handled in accordance with guidelines established by Keio University, Kyoto University, Tohoku Medical and Pharmaceutical University, FBRI and RIKEN‐BDR. All electroporations in this report were performed on E14 embryos.

*In utero* electroporation experiments were performed as described previously with minor modifications (Kawauchi *et al*, 2003). Pregnant mice were deeply anesthetized and an abdominal or right dorsal incision was made to access the uterus. Approximately 1 μl of plasmid DNA (shRNA experiments: 3 μg/μl, low concentration of Ncad‐sh1023: 1 μg/μl, rescue experiments: 1–10 μg/μl, pCAG‐EGFP: 0.5 μg/μl) in endotoxin‐free TE buffer (Qiagen) containing Fast Green was injected into the lateral ventricle of embryonic brains with a glass micropipette (GD‐1, Narishige). Holding the embryo *in utero* with forceps‐type electrodes (NEPA GENE or BEX), 50 ms electric pulses of 35 V were delivered five times at intervals of 450 ms with a square electroporator (NEPA21, NEPA GENE or CUY21, BEX). After electroporation, the uterus was placed back into the abdominal cavity, allowing embryos to continue developing. At indicated stages, embryos were harvested and coronal sections of electroporated brains were prepared by using a cryostat (Leica).

### Cortical slice cultures

Slice culture of embryonic cerebral cortices was performed as described previously (Nishimura *et al*, 2010). E16 embryonic brains, electroporated at E14, were cut into 300 μm coronal slices with a microtome (Leica) in DMEM/F‐12 1:1 media (Invitrogen). Cortical slices were cultured on the insert membranes (Millipore) in 2 ml of enriched media (100 μg/ml transferrin, 25 μg/ml insulin, 20 nM progesterone, 60 μM putrescine, 10 ng/ml EGF, 10 ng/ml bFGF, 5% Fetal bovine serum and 5% Horse serum; Miyata *et al*, 2002) in a CO_2_/O_2_ incubator (37°C, 5% CO_2_, 40 or 60% O_2_) under confocal laser scanning time‐lapse microscopy, FV1000 (Olympus) or TCL‐SP2 (Leica).

### Immunohistochemistry

Immunohistochemical analyses were performed as described previously with minor modifications (Kawauchi *et al*, 2010; Nishimura *et al*, 2014). Embryonic brains were fixed in 4% paraformaldehyde (PFA) in phosphate buffered saline (PBS) for several hours at 4°C. Frozen cortical sections were washed with PBS, treated with GS‐PBS (10% goat serum in PBS) or DS‐PBS (10% donkey serum in PBS) containing 0.05% Triton X‐100 for 1 h at room temperature (RT) and subsequently incubated with diluted primary antibodies in GS‐PBT (GS‐PBS containing 0.1% Tween 20) or DS‐PBT (DS‐PBS containing 0.1% Tween 20) at 4°C overnight. After three washes in PBS, sections were treated with Alexa488‐, Alexa555‐, or Alexa647‐conjugated secondary antibodies (Molecular Probes) diluted in PBS for 1 h at RT, followed by three washes in PBS. The nuclei were stained with DAPI. Fluorescence images were obtained by TCL‐SP5 laser scanning confocal microscopy (Leica) or A1R laser scanning confocal microscopy with a high sensitivity GaAsP detector (Nikon). For high‐resolution images, confocal images were obtained by Nikon A1R using the narrowed pinhole (0.5 or 0.6 Airy unit) and subjected to deconvolution processing with the Richardson–Lucy algorithm in the NIS‐ER software (Nikon). For staining with anti‐Ki67 or anti‐PH3 or anti‐Rab21 or anti‐Caveolin‐1 (only for high‐resolution images) antibodies, frozen cortical sections were treated with HistoVT‐One (Nacalai) for 20 min at 70°C after the fixation.

### Primary cultures, transfection, and immunocytochemistry

Primary culture of embryonic cortical neurons was performed as described previously with minor modifications (Kawauchi *et al*, 2006). E15 mouse embryonic cerebral cortices were treated with 0.25% Trypsin–EDTA for 10–15 min at 37°C and dissociated into single cells by gentle trituration. Cells were suspended in 500–1,000 μl of Neurobasal medium or Neurobasal plus medium (Invitrogen) supplemented with B‐27 or B‐27 plus (Invitrogen), 2 mM L‐glutamine (Sigma; Invitrogen) or GlutaMAX (Invitrogen) and 100 unit/ml penicillin and 0.1 mg/ml streptomycin (Sigma), and then plated on coverslips or 6 cm dishes or six‐well plates or 12‐well plates coated with 0.1 or 1 mg/ml poly‐D‐lysine (Sigma). Cells were incubated at 37°C for 2 or 5 days. Transfections into primary cultures of E15 cerebral cortices were performed using Amaxa mouse neuron nucleofector kit (Lonza) according to the manufacturer's instructions with some modifications.

For immunocytochemistry, cells were fixed with 4% PFA in PBS for 20 min, permeabilized with GS‐PBS or DS‐PBS containing 0.15% Triton X‐100 for 5 min, and blocked with GS‐PBS or DS‐PBS for 1 h at RT. Primary and secondary antibodies were treated as described above for immunohistochemistry. The nuclei were stained with DAPI. Fluorescence images were obtained by TCL‐SP5 laser scanning confocal microscopy (Leica) or A1R laser scanning confocal microscopy with a high‐sensitivity GaAsP detector (Nikon). For high‐resolution images, confocal images were obtained by Nikon A1R with the narrowed pinhole (0.3 Airy unit) and subjected to deconvolution processing with the Richardson–Lucy algorithm in the NIS‐ER software (Nikon).

### BODIPY‐LacCer or CTxB or transferrin uptake assay

E15 cerebral cortices were dissociated and cultured for 2 days. Primary cultured neurons were incubated with 5 μM BODIPY‐FL C5‐LacCer (Molecular Probes) in OPTI‐MEM medium (Invitrogen) for 10 min on ice and further incubated for 30 min at 37°C. Cells were washed six times with OPTI‐MEM containing 1% BSA for 10 min at 10°C and fixed with 4% PFA in PBS for 20 min. For CTxB uptake assay, primary cultured neurons were incubated with 5 μg/ml Alexa555‐conjugated Cholera Toxin Subunit B (CTxB) (Molecular Probes) in OPTI‐MEM for 10 min on ice, washed with Neurobasal medium and further incubated with Neurobasal medium for 30 min at 37°C. After fixation with 4% PFA in PBS for 20 min, cells were subjected to immunocytochemical analyses. For transferrin uptake assay, primary cultured neurons were incubated in OPTI‐MEM media for 30 min at 37°C and treated with 20 μg/ml Alexa594‐ or Alexa555‐conjugated transferrin (Tf) (Molecular Probes) in OPTI‐MEM media. After incubation for 5–15 min on ice or for 30 min at 10°C, cultured media was replaced with preincubated neurobasal media without Alexa dye‐conjugated Tf. Subsequently, neurons were incubated for 10 or 30 min at 37°C and fixed with 4% PFA in PBS for 20 min.

### Cell surface biotinylation assay

Primary cultured cortical neurons (2 DIV) were incubated with 0.5 mg/ml Ezlink sulfo‐NHS‐biotin (Thermo Fisher Scientific) in PBS for 30 min at 4°C under gentle agitation. After the incubation, cells were washed twice with 50 mM glycine in PBS. Subsequently, cells were treated with lysis buffer (20 mM Tris–HCl [pH 7.5], 150 mM NaCl, 1% Triton X‐100 and EDTA‐free Complete protease inhibitor cocktail [Roche]) and harvested with a cell scraper. After 30‐min incubation on ice, the lysates were sonicated and centrifuged at 8,000 *g* for 5 min at 4°C to remove cell debris.

The clarified supernatant containing the solubilized biotinylated proteins were precipitated with 50% streptavidin‐sepharose slurry (GE Healthcare) at 4°C overnight. Streptavidin‐sepharose beads were extensively washed in Lysis buffer and then resuspended in SDS sample buffer (50 mM Tris–HCl (pH 6.8), 2% SDS, 10% glycerol, 100 mM dithiothreitol (DTT) and bromophenol blue) and boiled for 5 min.

### Bafilomycin A1 treatment

E15 cerebral cortices were dissociated and cultured for 2 days. Primary cultured neurons (2 DIV) were incubated with 160 nM Bafilomycin A1 (Sigma) in Neurobasal medium for 6 h. Cells were fixed with 4% PFA in PBS for 20 min and subjected to immunocytochemistry. For immunoblotting, E15 cerebral cortices were dissociated and cultured for 1 day. Primary cultured neurons (1 DIV) were incubated with 160 nM Bafilomycin A1 (Sigma) in Neurobasal medium for 22 h. Cells were treated with lysis buffer, harvested with cell scraper, and subjected to immunoblot analyses.

### CD44 antibody feeding assay

CD44 antibody feeding assay was performed as described previously with minor modifications (Maldonado‐Baez *et al*, 2013). E15 cerebral cortices were dissociated and cultured on 18 mm coverslips for 2 days. The cultured primary cortical neurons were incubated with 10 μg/ml anti‐CD44 antibody for 1 h at 37°C. After antibody internalization, cells were washed twice with PBS and treated with low‐pH solution (0.5% acetic acid and 0.5 M NaCl, pH3.0) for 5 s to remove cell surface antibodies that were not internalized, followed by two rinses with PBS. Subsequently, cells were fixed with 2% PFA in PBS for 10 min and washed twice with PBS. The CD44 antibodies retained on the cell surface were blocked by incubating with unlabeled goat anti‐mouse IgG (1:100, BD Biosciences) in PBS for 30 min at room temperature. Internalized CD44 antibodies were labeled by incubating with Alexa Fluor 555‐conjugated secondary antibody in a PBS‐based buffer containing 10% FBS and 0.1% saponin for 30 min at room temperature. Coverslips were washed twice with PBS buffer containing 10% FBS for 15 min and mounted using prolong diamond Antifade Mountant with DAPI (Invitrogen).

### Immunoblotting

Immunoblot analyses were performed as described previously with minor modifications (Kawauchi *et al*, 2006; Nishimura *et al*, 2010).

For preparing cell lysates, primary cultured neurons were washed with ice‐cold PBS, treated with lysis buffer (20 mM Tris–HCl [pH 7.5], 150 mM NaCl, 1% Triton X‐100, EDTA‐free Complete protease inhibitor cocktail [Roche] and Phosphatase inhibitor cocktail [EDTA‐free; Nacalai Tesque]) and harvested with a cell scraper. After 1‐h incubation on ice, the lysates were sonicated and centrifuged at 5,000 rpm for 5 min at 4°C to remove cell debris. The supernatants were mixed with SDS sample buffer (50 mM Tris–HCl [pH 6.8], 2% SDS, 10% glycerol, 100 mM dithiothreitol [DTT] and bromophenol blue).

Cell lysates in the SDS sample buffer were separated with SDS–PAGE and transferred by electrophoresis onto polyvinylidene difluoride (PVDF) membranes. Membranes were blocked with 5% skim milk in TBST (20 mM Tris–HCl [pH 7.5], 150 mM NaCl, and 0.05% Tween20) or Blocking One (Nacalai Tesque) for 1 h and probed with primary antibodies in 5% skim milk in TBST or Can Get Signal reagents (TOYOBO), followed by treatment with horseradish peroxidase‐conjugated secondary antibodies and ECL Plus or ECL Prime Western blotting detection reagents (Amersham). Signals were detected with a cooled CCD camera (LAS‐4000mini, Fujifilm or FUSION‐FX7.EDGE, Vilber‐Lourmat) and measured with the Multi Gauge software (Fujifilm) or Image J.

### Quantitative real‐time PCR

Total RNA was extracted from primary cultured cortical neurons (2 DIV) using RNeasy Mini Kit (Qiagen) and transcribed into first strand cDNA using ReverTra Ace qPCR RT Master Mix with gDNA Remover (TOYOBO), according to the manufacturer's instructions. Quantitative real‐time PCR was performed with StepOnePlus Real‐Time PCR System (Applied Biosystems) using TaqMan Expression Assay Probes for caveolin‐1 (assay ID: Mm04260140_s1) and 18S RNA (4319413E) (Applied Biosystems). Relative expression levels were calculated by the relative standard curve method using 18S RNA as the internal control.

### Cell line culture and transfection

Human epithelial HeLa cells were cultured in minimum essential media (MEM) (Nacalai) with 10% fetal bovine serum (Gibco), 100 unit/ml penicillin and 0.1 mg/ml streptomycin (Sigma). NIH3T3 mouse fibroblasts (JCRB0615, clone 5611, JCRB) were cultured in Dulbecco's modified Eagle media (DMEM) (Nacalai) with 10% calf serum (CCT/Sanko Junyaku), 100 unit/ml penicillin and 0.1 mg/ml streptomycin. African green monkey fibroblasts, COS1 cells, were cultured in DMEM medium with 10% fetal bovine serum, 100 unit/ml penicillin and 0.1 mg/ml streptomycin. HeLa and COS‐1 cells were provided from Dr. Michisuke Yuzaki, Keio University.

For transfection of cultured cell lines, plasmid DNAs were transfected using Lipofectamine 3000 reagent (Thermo Fisher Scientific), according to the manufacturer's instructions, and the transfected cells were incubated for an additional 24 h.

### Quantitative analysis for the ratio of cells with different morphology

Morphology of the immature neurons in the IZ was analyzed on frozen sections of the cerebral cortices at E17, 3 days after electroporation. The numbers of cells with a locomoting or round or multipolar (without a leading process) were counted in the IZ. Locomoting cells were defined as cells with a thick and pia‐directed leading process. Using our definition, both round and multipolar cells do not possess a leading process. Multipolar cells were defined as cells with more than three neurites or polygonal morphology.

### Quantitative analysis for the leading process length and branching and the number of primary neurites

The ratio of the locomoting neurons (leading process‐possessing cells) with a branched leading process or more than three primary neurites to the total number of the locomoting neurons in the IZ was counted on frozen sections of the cerebral cortices at E17, 3 days after electroporation. Leading process length of the locomoting neurons in the IZ was measured by the Leica SP5 or Nikon NIS elements software.

### Quantitative analysis for the neuronal positioning

The extent of migration was estimated by recording fluorescence intensities of EGFP in distinct regions of the cerebral cortices, as described previously (Kawauchi *et al*, 2003, 2006). Fluorescence images of the frozen sections of the electroporated brains were captured by TCS‐SP5 laser scanning confocal microscopy (Leica). Fluorescence intensities within the same width regions in the layers II‐IV, V‐VI, IZ, and SVZ/VZ of the cerebral cortices were measured by the Leica SP5 software. Relative intensities to the total fluorescence were calculated and plotted in the graphs with standard errors. For cell quantification, cell numbers within the same width regions in the layers II‐IV, V‐VI, IZ, and SVZ/VZ of the cerebral cortices were counted and the ratios of the cell numbers in each layer to total cell numbers were calculated and plotted in the graphs with standard errors.

### Quantitative analysis for colocalization

The efficiency of colocalization of two proteins in neurons was measured, and the Pearson's correlation coefficient was calculated using NIS elements software (Nikon) or Image J.

### Quantitative estimation of cell surface levels of N‐cadherin

Primary cortical neurons (2 DIV) were washed with ice‐cold PBS and fixed with 4% PFA in PBS for 20 min on ice. After two washes with PBS, cells were blocked with DS‐PBS for 30 min at RT and incubated with diluted primary antibody (anti‐N‐cadherin antibody [C3865, Sigma] for surface N‐cadherin staining) in DS‐PBS for 60 min at RT. After three washes in PBS, cells were permeabilized with DS‐PBS containing 0.15% Triton X‐100 for 5 min at RT, and incubated with diluted primary antibodies (anti‐N‐cadherin antibody [sc‐7939, Santa Cruz Biotechnology] for total N‐cadherin and anti‐GFP chick antibody [AB16901, Millipore]) in DS‐PBT at 4°C overnight. Secondary antibodies were applied as described above. After three washes in PBS, cells were treated with Alexa488‐ or Alexa555‐conjugated secondary antibodies (Molecular Probes) diluted in PBS for 60 min at RT, followed by three washes in PBS.

### Quantitative estimation of N‐cadherin localization on the plasma membrane and early endosomes

Primary cortical neurons were transfected as indicated with pCAG‐PM‐mAG1, pCAG‐HA‐N‐cadherin and Rab21‐sh115 or control vectors. After 2 days of culture *in vitro*, cells were fixed with 4% PFA in PBS for 20 min and subjected to immunocytochemical analyses for anti‐HA and anti‐APPL1 antibodies. Fluorescence intensities of HA‐tagged N‐cadherin in the PM‐mAG1‐positive region (plasma membrane) and APPL1‐positive region (early endosomes) in each neuron were measured using the NIS elements software (Nikon). The ratio of the fluorescence intensities in the PM‐mAG1‐ or APPL1‐positive regions to that of whole cells was calculated.

For *in vivo* analyses, frozen sections of E17 cerebral cortices, electroporated with pCAG‐PM‐mAG1, pCAG‐HA‐N‐cadherin and Rab21‐sh115 or control vectors at E14, were examined immunohistochemically with anti‐HA antibody. Fluorescence intensities of HA‐tagged N‐cadherin in the PM‐mAG1‐positive region (plasma membrane) in each neuron were measured using the NIS elements software (Nikon). The ratio of the fluorescence intensities in the PM‐mAG1‐positive regions to that of whole cells was calculated.

### Quantitative estimation of the plasma membrane localization

Primary cortical neurons were transfected as indicated with pCAG‐PM‐mAG1 and Rab21‐sh115 or Cav1‐sh490 or control vectors. After 2 days of culture *in vitro*, cells were fixed with 4% PFA in PBS for 20 min and subjected to immunocytochemical analyses for anti‐caveolin‐1 or anti‐Rab21 or anti‐Rab5 antibodies. Fluorescence intensities of endogenous caveolin‐1 in the PM‐mAG1‐positive region (plasma membrane) in each neuron were measured using Image J.

#### Statistical analyses

Statistical significance was calculated using two‐tailed Student's *t*‐test (for data showing normal distribution and equality of variance), Welch's *t*‐test (for data showing normal distribution, but not equality of variance), Mann–Whitney's *U* test (for data that are not normal distribution or for categorical data), paired *t*‐test (for paired sample) or multiple comparison analyses (one‐way ANOVA with *post hoc* Tukey–Kramer test (parametric), Dunnett test (parametric) and Kruskal–Wallis test with *post hoc* Steel–Dwass test (nonparametric)), using the Statcel3 software (OMS). A *P*‐value of < 0.05 was considered statistically significant. Sample size are shown in the corresponding figure legends, and the number of technical replicates is greater than two.

## Author contributions

**Mima Shikanai:** Investigation. **Shiho Ito:** Formal analysis; validation; investigation; visualization; methodology; writing—review and editing. **Yoshiaki V Nishimura:** Formal analysis; investigation. **Remi Akagawa:** Investigation. **Mitsunori Fukuda:** Resources; investigation; writing—review and editing. **Michisuke Yuzaki:** Resources; supervision; writing—review and editing. **Yo‐ichi Nabeshima:** Supervision. **Takeshi Kawauchi:** Conceptualization; data curation; formal analysis; supervision; funding acquisition; investigation; visualization; methodology; writing—original draft; project administration.

## Disclosure and competing interests statement

The authors declare that they have no conflict of interest.

## Supporting information



Appendix S1

Expanded View Figures PDF

PDF+

## Data Availability

This study includes no data deposited in public repositories.

## References

[embr202254701-bib-0001] Allen JA , Yadav PN , Setola V , Farrell M , Roth BL (2011) Schizophrenia risk gene CAV1 is both pro‐psychotic and required for atypical antipsychotic drug actions *in vivo* . Transl Psychiatry 1: e33 22832607 10.1038/tp.2011.35 PMC3309505

[embr202254701-bib-0002] Ariotti N , Parton RG (2013) SnapShot: caveolae, caveolins, and cavins. Cell 154: 704–704.e1 23911330 10.1016/j.cell.2013.07.009

[embr202254701-bib-0003] Bitsikas V , Correa IR Jr , Nichols BJ (2014) Clathrin‐independent pathways do not contribute significantly to endocytic flux. Elife 3: e03970 25232658 10.7554/eLife.03970 PMC4185422

[embr202254701-bib-0004] Boucrot E , Ferreira AP , Almeida‐Souza L , Debard S , Vallis Y , Howard G , Bertot L , Sauvonnet N , McMahon HT (2015) Endophilin marks and controls a clathrin‐independent endocytic pathway. Nature 517: 460–465 25517094 10.1038/nature14067

[embr202254701-bib-0005] Burana D , Yoshihara H , Tanno H , Yamamoto A , Saeki Y , Tanaka K , Komada M (2016) The Ankrd13 family of ubiquitin‐interacting motif‐bearing proteins regulates Valosin‐containing protein/p97 protein‐mediated lysosomal trafficking of Caveolin 1. J Biol Chem 291: 6218–6231 26797118 10.1074/jbc.M115.710707 PMC4813590

[embr202254701-bib-0006] Chaudhary N , Gomez GA , Howes MT , Lo HP , McMahon KA , Rae JA , Schieber NL , Hill MM , Gaus K , Yap AS *et al* (2014) Endocytic crosstalk: cavins, caveolins, and caveolae regulate clathrin‐independent endocytosis. PLoS Biol 12: e1001832 24714042 10.1371/journal.pbio.1001832 PMC3979662

[embr202254701-bib-0007] Damke H , Baba T , van der Bliek AM , Schmid SL (1995) Clathrin‐independent pinocytosis is induced in cells overexpressing a temperature‐sensitive mutant of dynamin. J Cell Biol 131: 69–80 7559787 10.1083/jcb.131.1.69 PMC2120592

[embr202254701-bib-0008] Del Olmo T , Lauzier A , Normandin C , Larcher R , Lecours M , Jean D , Lessard L , Steinberg F , Boisvert FM , Jean S (2019) APEX2‐mediated RAB proximity labeling identifies a role for RAB21 in clathrin‐independent cargo sorting. EMBO Rep 20: e47192 30610016 10.15252/embr.201847192 PMC6362359

[embr202254701-bib-0009] Diaz J , Mendoza P , Ortiz R , Diaz N , Leyton L , Stupack D , Quest AF , Torres VA (2014) Rab5 is required in metastatic cancer cells for Caveolin‐1‐enhanced Rac1 activation, migration and invasion. J Cell Sci 127: 2401–2406 24659799 10.1242/jcs.141689 PMC4074264

[embr202254701-bib-0010] Dotti CG , Sullivan CA , Banker GA (1988) The establishment of polarity by hippocampal neurons in culture. J Neurosci 8: 1454–1468 3282038 10.1523/JNEUROSCI.08-04-01454.1988 PMC6569279

[embr202254701-bib-0011] Egami Y , Araki N (2009) Dynamic changes in the spatiotemporal localization of Rab21 in live RAW264 cells during macropinocytosis. PLoS One 4: e6689 19693279 10.1371/journal.pone.0006689 PMC2726762

[embr202254701-bib-0012] Ehlers MD , Polleux F (2010) Neuronal and glial cell biology. Curr Opin Neurobiol 20: 529–530 20678922 10.1016/j.conb.2010.06.004

[embr202254701-bib-0013] Franke C , Repnik U , Segeletz S , Brouilly N , Kalaidzidis Y , Verbavatz JM , Zerial M (2019) Correlative single‐molecule localization microscopy and electron tomography reveals endosome nanoscale domains. Traffic 20: 601–617 31206952 10.1111/tra.12671 PMC6771687

[embr202254701-bib-0014] Grande‐Garcia A , Echarri A , de Rooij J , Alderson NB , Waterman‐Storer CM , Valdivielso JM , del Pozo MA (2007) Caveolin‐1 regulates cell polarization and directional migration through Src kinase and rho GTPases. J Cell Biol 177: 683–694 17517963 10.1083/jcb.200701006 PMC2064213

[embr202254701-bib-0015] Hagiwara M , Shirai Y , Nomura R , Sasaki M , Kobayashi K , Tadokoro T , Yamamoto Y (2009) Caveolin‐1 activates Rab5 and enhances endocytosis through direct interaction. Biochem Biophys Res Commun 378: 73–78 19013132 10.1016/j.bbrc.2008.10.172

[embr202254701-bib-0016] Hayer A , Stoeber M , Ritz D , Engel S , Meyer HH , Helenius A (2010) Caveolin‐1 is ubiquitinated and targeted to intralumenal vesicles in endolysosomes for degradation. J Cell Biol 191: 615–629 21041450 10.1083/jcb.201003086 PMC3003328

[embr202254701-bib-0017] Howes MT , Mayor S , Parton RG (2010) Molecules, mechanisms, and cellular roles of clathrin‐independent endocytosis. Curr Opin Cell Biol 22: 519–527 20439156 10.1016/j.ceb.2010.04.001

[embr202254701-bib-0018] Kalaidzidis I , Miaczynska M , Brewińska‐Olchowik M , Hupalowska A , Ferguson C , Parton RG , Kalaidzidis Y , Zerial M (2015) APPL endosomes are not obligatory endocytic intermediates but act as stable cargo‐sorting compartments. J Cell Biol 211: 123–144 26459602 10.1083/jcb.201311117 PMC4602042

[embr202254701-bib-0019] Kawauchi T (2015) Cellullar insights into cerebral cortical development: focusing on the locomotion mode of neuronal migration. Front Cell Neurosci 9: 394 26500496 10.3389/fncel.2015.00394 PMC4595654

[embr202254701-bib-0020] Kawauchi T , Hoshino M (2008) Molecular pathways regulating cytoskeletal organization and morphological changes in migrating neurons. Dev Neurosci 30: 36–46 18075253 10.1159/000109850

[embr202254701-bib-0021] Kawauchi T , Chihama K , Nabeshima Y , Hoshino M (2003) The *in vivo* roles of STEF/Tiam1, Rac1 and JNK in cortical neuronal migration. EMBO J 22: 4190–4201 12912917 10.1093/emboj/cdg413 PMC175802

[embr202254701-bib-0022] Kawauchi T , Chihama K , Nishimura YV , Nabeshima Y , Hoshino M (2005) MAP1B phosphorylation is differentially regulated by Cdk5/p35, Cdk5/p25, and JNK. Biochem Biophys Res Commun 331: 50–55 15845356 10.1016/j.bbrc.2005.03.132

[embr202254701-bib-0023] Kawauchi T , Chihama K , Nabeshima Y , Hoshino M (2006) Cdk5 Phosphorylates and stabilizes p27kip1 contributing to Actin organization and cortical neuronal migration. Nat Cell Biol 8: 17–26 16341208 10.1038/ncb1338

[embr202254701-bib-0024] Kawauchi T , Sekine K , Shikanai M , Chihama K , Tomita K , Kubo K , Nakajima K , Nabeshima Y , Hoshino M (2010) Rab GTPases‐dependent endocytic pathways regulate neuronal migration and maturation through N‐cadherin trafficking. Neuron 67: 588–602 20797536 10.1016/j.neuron.2010.07.007

[embr202254701-bib-0025] Macdonald JL , Pike LJ (2005) A simplified method for the preparation of detergent‐free lipid rafts. J Lipid Res 46: 1061–1067 15722565 10.1194/jlr.D400041-JLR200

[embr202254701-bib-0026] Maldonado‐Baez L , Cole NB , Kramer H , Donaldson JG (2013) Microtubule‐dependent endosomal sorting of clathrin‐independent cargo by Hook1. J Cell Biol 201: 233–247 23589492 10.1083/jcb.201208172 PMC3628520

[embr202254701-bib-0027] Marin O , Valiente M , Ge X , Tsai LH (2010) Guiding neuronal cell migrations. Cold Spring Harb Perspect Biol 2: a001834 20182622 10.1101/cshperspect.a001834 PMC2828271

[embr202254701-bib-0028] Mayle KM , Le AM , Kamei DT (2012) The intracellular trafficking pathway of transferrin. Biochim Biophys Acta 1820: 264–281 21968002 10.1016/j.bbagen.2011.09.009 PMC3288267

[embr202254701-bib-0029] Miaczynska M , Christoforidis S , Giner A , Shevchenko A , Uttenweiler‐Joseph S , Habermann B , Wilm M , Parton RG , Zerial M (2004) APPL proteins link Rab5 to nuclear signal transduction via an endosomal compartment. Cell 116: 445–456 15016378 10.1016/s0092-8674(04)00117-5

[embr202254701-bib-0030] Miyata T , Kawaguchi A , Saito K , Kuramochi H , Ogawa M (2002) Visualization of cell cycling by an improvement in slice culture methods. J Neurosci Res 69: 861–868 12205679 10.1002/jnr.10335

[embr202254701-bib-0031] Mori Y , Fukuda M (2013) Rabex‐5 determines the neurite localization of its downstream Rab proteins in hippocampal neurons. Commun Integr Biol 6: e25433 24265856 10.4161/cib.25433 PMC3829932

[embr202254701-bib-0032] Mu FT , Callaghan JM , Steele‐Mortimer O , Stenmark H , Parton RG , Campbell PL , McCluskey J , Yeo JP , Tock EP , Toh BH (1995) EEA1, an early endosome‐associated protein. EEA1 is a conserved alpha‐helical peripheral membrane protein flanked by cysteine "fingers" and contains a calmodulin‐binding IQ motif. J Biol Chem 270: 13503–13511 7768953 10.1074/jbc.270.22.13503

[embr202254701-bib-0033] Nakashima H , Hamamura K , Houjou T , Taguchi R , Yamamoto N , Mitsudo K , Tohnai I , Ueda M , Urano T , Furukawa K *et al* (2007) Overexpression of caveolin‐1 in a human melanoma cell line results in dispersion of ganglioside GD3 from lipid rafts and alteration of leading edges, leading to attenuation of malignant properties. Cancer Sci 98: 512–520 17284246 10.1111/j.1349-7006.2007.00419.x PMC11159806

[embr202254701-bib-0034] Nishimura YV , Sekine K , Chihama K , Nakajima K , Hoshino M , Nabeshima Y , Kawauchi T (2010) Dissecting the factors involved in the locomotion mode of neuronal migration in the developing cerebral cortex. J Biol Chem 285: 5878–5887 20022952 10.1074/jbc.M109.033761 PMC2820813

[embr202254701-bib-0035] Nishimura YV , Shikanai M , Hoshino M , Ohshima T , Nabeshima Y , Mizutani K , Nagata K , Nakajima K , Kawauchi T (2014) Cdk5 and its substrates, dcx and p27kip1, regulate cytoplasmic dilation formation and nuclear elongation in migrating neurons. Development 141: 3540–3550 25183872 10.1242/dev.111294

[embr202254701-bib-0036] Noctor SC , Martinez‐Cerdeno V , Ivic L , Kriegstein AR (2004) Cortical neurons arise in symmetric and asymmetric division zones and migrate through specific phases. Nat Neurosci 7: 136–144 14703572 10.1038/nn1172

[embr202254701-bib-0037] Ohbayashi N , Yatsu A , Tamura K , Fukuda M (2012) The Rab21‐GEF activity of Varp, but not its Rab32/38 effector function, is required for dendrite formation in melanocytes. Mol Biol Cell 23: 669–678 22171327 10.1091/mbc.E11-04-0324 PMC3279394

[embr202254701-bib-0038] Opdam FJM , Kamps G , Croes H , van Bokhoven H , Ginsel LA , Fransen JAM (2000) Expression of Rab small GTPases in epithelial Caco‐2 cells: Rab21 is an apically located GTP‐binding protein in polarised intestinal epithelial cells. Eur J Cell Biol 79: 308–316 10887961 10.1078/S0171-9335(04)70034-5

[embr202254701-bib-0039] Parton RG (1994) Ultrastructural localization of gangliosides; GM1 is concentrated in caveolae. J Histochem Cytochem 42: 155–166 8288861 10.1177/42.2.8288861

[embr202254701-bib-0040] Parton RG (2018) Caveolae: structure, function, and relationship to disease. Annu Rev Cell Dev Biol 34: 111–136 30296391 10.1146/annurev-cellbio-100617-062737

[embr202254701-bib-0041] Pelkmans L , Zerial M (2005) Kinase‐regulated quantal assemblies and kiss‐and‐run recycling of caveolae. Nature 436: 128–133 16001074 10.1038/nature03866

[embr202254701-bib-0042] Pelkmans L , Burli T , Zerial M , Helenius A (2004) Caveolin‐stabilized membrane domains as multifunctional transport and sorting devices in endocytic membrane traffic. Cell 118: 767–780 15369675 10.1016/j.cell.2004.09.003

[embr202254701-bib-0043] Renard HF , Boucrot E (2021) Unconventional endocytic mechanisms. Curr Opin Cell Biol 71: 120–129 33862329 10.1016/j.ceb.2021.03.001

[embr202254701-bib-0044] Renard HF , Simunovic M , Lemiere J , Boucrot E , Garcia‐Castillo MD , Arumugam S , Chambon V , Lamaze C , Wunder C , Kenworthy AK *et al* (2015) Endophilin‐A2 functions in membrane scission in clathrin‐independent endocytosis. Nature 517: 493–496 25517096 10.1038/nature14064 PMC4342003

[embr202254701-bib-0045] Rink J , Ghigo E , Kalaidzidis Y , Zerial M (2005) Rab conversion as a mechanism of progression from early to late endosomes. Cell 122: 735–749 16143105 10.1016/j.cell.2005.06.043

[embr202254701-bib-0046] Shieh JC , Schaar BT , Srinivasan K , Brodsky FM , McConnell SK (2011) Endocytosis regulates cell soma translocation and the distribution of adhesion proteins in migrating neurons. PLoS One 6: e17802 21445347 10.1371/journal.pone.0017802 PMC3062553

[embr202254701-bib-0047] Shikanai M , Nishimura YV , Sakurai M , Nabeshima YI , Yuzaki M , Kawauchi T (2018) Caveolin‐1 promotes early neuronal maturation via Caveolae‐independent trafficking of N‐cadherin and L1. iScience 7: 53–67 30267686 10.1016/j.isci.2018.08.014 PMC6135901

[embr202254701-bib-0048] Shvets E , Bitsikas V , Howard G , Hansen CG , Nichols BJ (2015) Dynamic caveolae exclude bulk membrane proteins and are required for sorting of excess glycosphingolipids. Nat Commun 6: 6867 25897946 10.1038/ncomms7867 PMC4410672

[embr202254701-bib-0049] Simpson JC , Griffiths G , Wessling‐Resnick M , Fransen JA , Bennett H , Jones AT (2004) A role for the small GTPase Rab21 in the early endocytic pathway. J Cell Sci 117: 6297–6311 15561770 10.1242/jcs.01560

[embr202254701-bib-0050] Singh RD , Puri V , Valiyaveettil JT , Marks DL , Bittman R , Pagano RE (2003) Selective caveolin‐1‐dependent endocytosis of glycosphingolipids. Mol Biol Cell 14: 3254–3265 12925761 10.1091/mbc.E02-12-0809 PMC181565

[embr202254701-bib-0051] Stenmark H (2009) Rab GTPases as coordinators of vesicle traffic. Nat Rev Mol Cell Biol 10: 513–525 19603039 10.1038/nrm2728

[embr202254701-bib-0052] Tabata H , Nakajima K (2003) Multipolar migration: the third mode of radial neuronal migration in the developing cerebral cortex. J Neurosci 23: 9996–10001 14602813 10.1523/JNEUROSCI.23-31-09996.2003 PMC6740853

[embr202254701-bib-0053] Tobys D , Kowalski LM , Cziudaj E , Müller S , Zentis P , Pach E , Zigrino P , Blaeske T , Höning S (2021) Inhibition of clathrin‐mediated endocytosis by knockdown of AP‐2 leads to alterations in the plasma membrane proteome. Traffic 22: 6–22 33225555 10.1111/tra.12770

[embr202254701-bib-0054] Trischler M , Stoorvogel W , Ullrich O (1999) Biochemical analysis of distinct Rab5‐ and Rab11‐positive endosomes along the transferrin pathway. J Cell Sci 112: 4773–4783 10574724 10.1242/jcs.112.24.4773

[embr202254701-bib-0055] Winckler B , Mellman I (2010) Trafficking guidance receptors. Cold Spring Harb Perspect Biol 2: a001826 20504966 10.1101/cshperspect.a001826 PMC2890194

[embr202254701-bib-0056] Yap AS , Crampton MS , Hardin J (2007) Making and breaking contacts: the cellular biology of cadherin regulation. Curr Opin Cell Biol 19: 508–514 17935963 10.1016/j.ceb.2007.09.008 PMC2128038

[embr202254701-bib-0057] Zerial M , McBride H (2001) Rab proteins as membrane organizers. Nat Rev Mol Cell Biol 2: 107–117 11252952 10.1038/35052055

