# Supplementary material for: Rab21 regulates caveolin‐1‐mediated endocytic trafficking to promote immature neurite pruning
Source: EMBO Rep. 2023 Jan 23;24(3):e54701. doi: 10.15252/embr.202254701 (PMC9986827; doi:10.15252/embr.202254701)
Supplement: Supplementary file 1 — Appendix S1 [file EMBR-24-e54701-s002.pdf]

Appendix

Rab21 regulates caveolin-1-mediated endocytic trafficking to promote immature neurite pruning

Mima Shikanai[1,*], Shiho Ito[2,3,*], Yoshiaki V. Nishimura[4], Remi Akagawa[3], Mitsunori Fukuda[5], Michisuke Yuzaki[1], Yo-ichi Nabeshima[2,3], Takeshi Kawauchi[1,2,3,§]

[*] M.S. and S.I. contributed equally to this work.

§ Correspondence: Takeshi Kawauchi

E-mail: kawauchi.takeshi.i92@kyoto-u.jp

Table of Contents:

Appendix Figure S1 – page 2

Appendix Figure S1

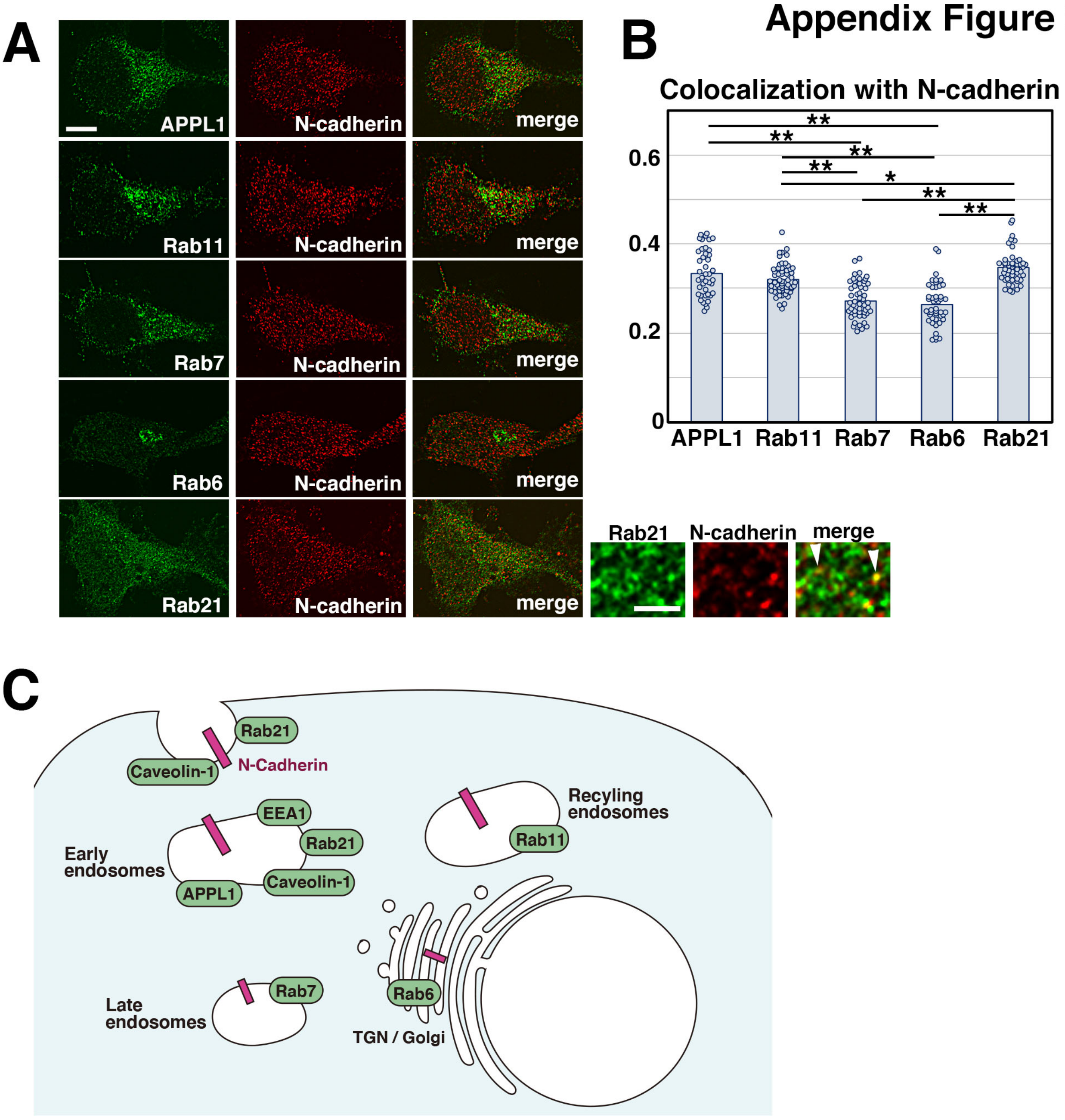

Appendix Figure S1. Endocytic trafficking pathways of N-cadherin in primary cortical neurons. (A-B) Primary cortical neurons from E15 cerebral cortices were incubated for two days *in vitro* and immunostained with the indicated antibodies. The images are obtained with high-resolution microscopy (Nikon). The graph in (B) shows the Pearson's correlation coefficient between N-cadherin and organelle markers. Each score represents the mean with the individual points. APPL1: n = 42 cells, Rab11: n = 57 cells, Rab7: n = 52 cells, Rab6: n = 46 cells, Rab21: n = 48 cells. (C) N-cadherin internalization is regulated by Rab21, as well as Rab5. Caveolin-1 localizes in the plasma membrane and early endosomes, but rarely colocalizes with Rab11. Because the internalized N-cadherin is mainly transported to the Rab11-positive recycling endosomes, caveolin-1 regulates the endocytosis and the early step of the endosomal trafficking of N-cadherin.

Data information: (B) Significance compared to control was determined by one-way ANOVA with post hoc Tukey-Kramer. Significant differences were observed between APPL1/Rab11/Rab21 (with N-cadherin) and Rab7/Rab6 (with N-cadherin). In addition, Rab21 exhibited higher efficiency of colocalization with N-cadherin, compared to Rab11. **: < the critical value at 1% (APPL1 and Rab7 or Rab6, Rab11 and Rab7 or Rab6, Rab21 and Rab7 or Rab6), *: < the critical value at 5% (Rab21 and Rab11). **: < the critical value at 1%. Scale bars:, 5 µm in (left panels in A), 1 µm in (right panels in A).
