# Supplementary material for: Rab21 regulates caveolin‐1‐mediated endocytic trafficking to promote immature neurite pruning
Source: EMBO Rep. 2023 Jan 23;24(3):e54701. doi: 10.15252/embr.202254701 (PMC9986827; doi:10.15252/embr.202254701)
Supplement: Supplementary file 2 — Expanded View Figures PDF [file EMBR-24-e54701-s004.pdf]

Expanded View Figures

Figure EV1. Rab5 and Rab21 localize in distinct vesicular compartments.

A–C Primary cortical neurons from E15 cerebral cortices incubated for 2 days *in vitro* and stained with the indicated antibodies. Right panels in (A) and (B) are high magnification images indicated by white rectangles. The images are obtained with high-resolution microscopy (Nikon). The graph in (C) shows the Pearson's correlation coefficient between EEA1 and Rab21 or Rab5. Each score represents the mean of ratios with the individual points. Rab21: $n = 29$ cells, Rab5: $n = 28$ cells.

D Primary cortical neurons from E15 cerebral cortices were transfected with EGFP-Rab21 and incubated for 2 days *in vitro*. To maintain moderate expression levels, a CMV promoter was used to express EGFP-Rab21. Cells were immunostained with anti-EGFP (green) and anti-Rab5 antibodies.

E Primary cortical neurons from E15 cerebral cortices were transfected with the indicated plasmids, incubated for 2 days *in vitro* and subjected to immunoblot analyses of cell lysates with the indicated antibodies.

F Immature neurons in the IZ of the cerebral cortices at E17, electroporated with the indicated plasmids plus pCAG-EGFP at E14. Frozen sections were immunostained with the indicated antibodies. The images are obtained with A1R with a high sensitivity GaAsP detector (Nikon). Blue alone channels are shown in black and white images.

G, H Primary cortical neurons from E15 cerebral cortices were transfected with the indicated plasmids, incubated for 2 days *in vitro* and subjected to cell surface biotinylation assay, followed by immunoblot analyses of cell lysates with anti-TfR antibody. The upper and lower panels indicate the precipitates with streptavidin-sepharose beads and lysates before pull down (input), respectively. The graph in (H) shows the ratio of cell surface biotinylated transferrin receptor (TfR) to total TfR. Each score represents the mean with the individual points. Control: $n = 5$ biological replicates, Rab21-sh115: $n = 5$ biological replicates, Rab5-sh232: $n = 5$ biological replicates, Cav1-sh490: $n = 5$ biological replicates. Significance was determined by one-way ANOVA with *post hoc* Dunnett. No significant difference was observed between control and Rab21-sh115 or Rab5-sh232 or Cav1-sh490. n.s., no significant differences.

Data information: (C) Significance was determined by Student's *t*-test ($P = 0.00007779$). $^{**}P < 0.01$. (H) Significance was determined by one-way ANOVA with *post hoc* Dunnett. No significant difference was observed between control and Rab21-sh115 or Rab5-sh232 or Cav1-sh490. n.s.: no significant differences. Scale bars: 5 μm in (left panels in A, B), 1 μm in (right panels in A, B), 3 μm in (D), 4 μm in (F).

© 2023 The Authors

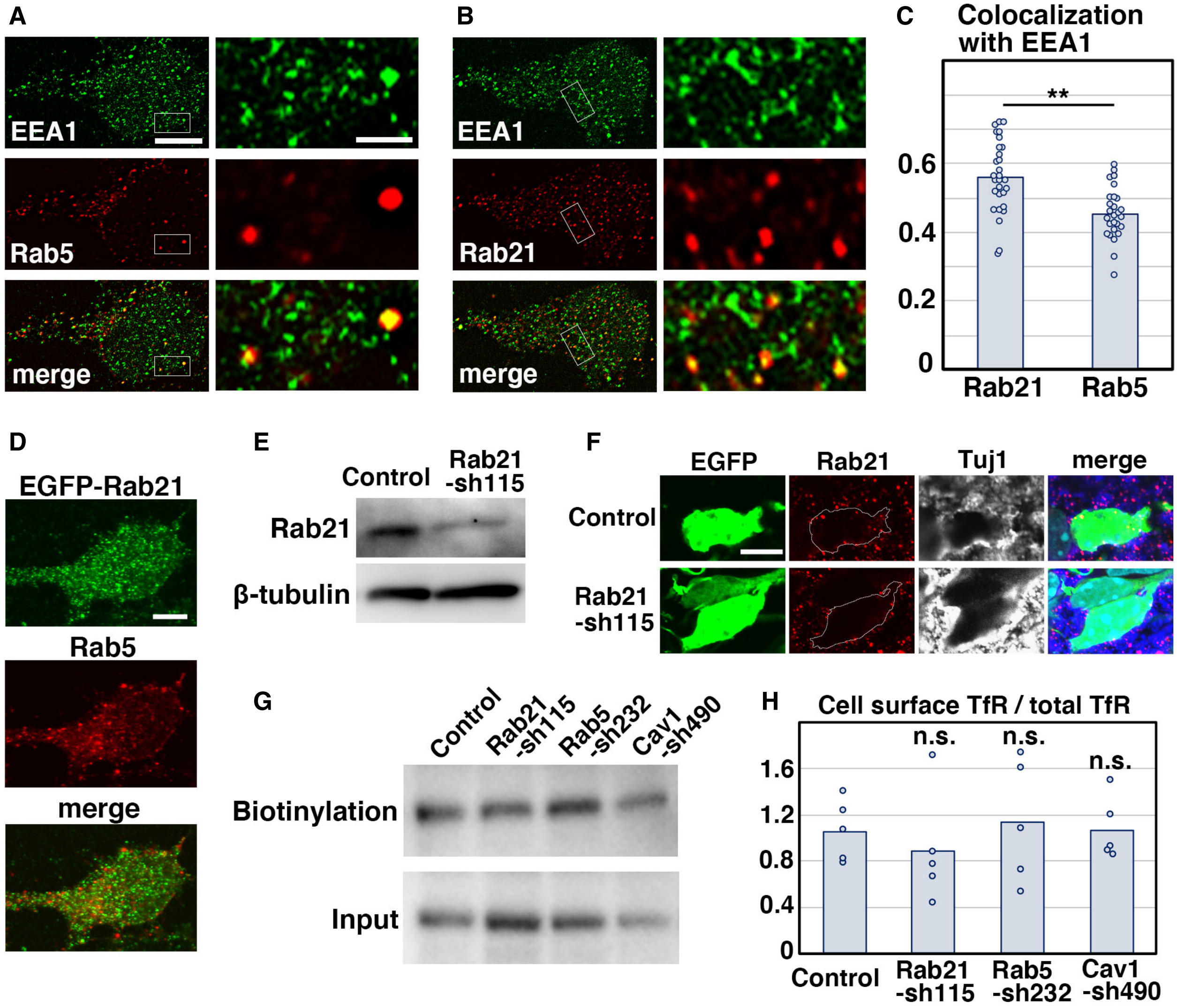

Figure EV1.

 © 2023 The Authors

Figure EV2. Knockdown of Rab21, as well as Rab5, suppresses cortical neuronal migration, but not the proliferation and differentiation of neural progenitors.

A, B Cerebral cortices at P0, electroporated with the indicated plasmids plus pCAG-EGFP at E14. The lower graphs in (B) show the estimation of cell migration, which was carried out by recording fluorescence intensities of EGFP in distinct regions of the cerebral cortices using Leica SP5 software. Each bar represents the mean percentage of relative intensities ± SEM. Control: $n = 6$ brains, Rab21-sh115: $n = 5$ brains, Rab21-sh115 + pCAG-wt-Rab21: $n = 5$ brains, Rab21-sh115 + pTα1-wt-Rab21: $n = 6$ brains. II-IV, layers II-IV of the cortical plate; IZ, intermediate zone; SVZ/VZ, subventricular zone/ventricular zone; V-VI, layers V-VI of the cortical plate; WM, white matter.

C The ratio of the number of the electroporated cells in the upper layers (layers II–IV). Each score represents the mean of ratios with the individual points. Control: $n = 5$ brains, Rab21-sh115: $n = 4$ brains, Rab21-sh115 + control vector: $n = 5$ brains, Rab21-sh115 + CAG-wt-Rab21: $n = 4$ brains, Rab21-sh115 + Tα1-wt-Rab21: $n = 5$ brains, Rab21-sh115 + wt-Caveolin-1: $n = 5$ brains. The sample numbers show biological replicates. In case of co-electroporation with wt-Rab21 driven by CAG promoter, the Rab21-sh115-mediated migration defect was partially rescued, but its efficiency was lower than that of Tα1-wt-Rab21, implicating that excess Rab21 in neural progenitors might have a negative effect on proper neuronal positioning.

D Cerebral cortices at E17, electroporated with the indicated plasmids plus pCAG-EGFP at E14. The graph shows the ratio of cells with the indicated morphology in the IZ. Control and Rab21-sh115: $n = 3$ brains.

E–G Cerebral cortices at E16 (46 h after electroporation) (E and G) and E17 (F), electroporated with the indicated plasmids plus pCAG-EGFP at E14. Frozen sections were immunostained with anti-EGFP and anti-phospho-Histone H3 (left panels in E) or anti-Ki67 (right panels in E) or anti-Tuj1 (F) antibodies. Blue alone channels are shown in black and white images. The graph in (G) shows the ratio of phospho-Histone H3-positive cells in the electroporated cells in the VZ. Each score represents the mean of ratios with the individual points. Control: $n = 6$ brains (762 cells), Rab21-sh115: $n = 6$ brains (374 cells).

Data information: (B) Significance compared to control was determined by Student's *t*-test (Rab21-sh115 [Layer II–IV]: $P = 0.000001254$, Rab21-sh115 [IZ]: 0.00008495). $^{**}P < 0.01$. (C) Significance was determined by one-way ANOVA with *post hoc* Tukey–Kramer test. *Less than the critical value at 5% (Control vs. Rab21-sh115 + ppCAG-wt-Rab21, Control vs. Rab21-sh115 + pCAG-wt-Caveolin-1), **less than the critical value at 1% (Control vs. Rab21-sh115, Control vs. Rab21-sh115 + control vector, Rab21-sh115 vs. Rab21-sh115 + pCAG-wt-Rab21, Rab21-sh115 vs. Rab21-sh115 + pTα1-wt-Rab21, Rab21-sh115 vs. Rab21-sh115 + pCAG-wt-Caveolin-1, Rab21-sh115 + control vector vs. Rab21-sh115 + pCAG-wt-Rab21, Rab21-sh115 + control vector vs. Rab21-sh115 + pTα1-wt-Rab21, Rab21-sh115 + control vector vs. Rab21-sh115 + pCAG-wt-Caveolin-1). (D) No significant differences (n.s.) between control and Rab21-sh115-electroporated neurons were found by Mann–Whitney's U test and Student's *t*-test (Locomotion: $P = 0.5127$ or 0.4841, Round: $P = 0.1266$ or 0.1916, Multipolar: $P = 0.2752$ or 0.1786, respectively). (G) Significance compared to control was determined by Student's *t*-test ($P = 0.1149$). n.s.: no significant differences. Scale bars: 100 μm in (A, B). 25 μm in (E), 5 μm in (F).

© 2023 The Authors

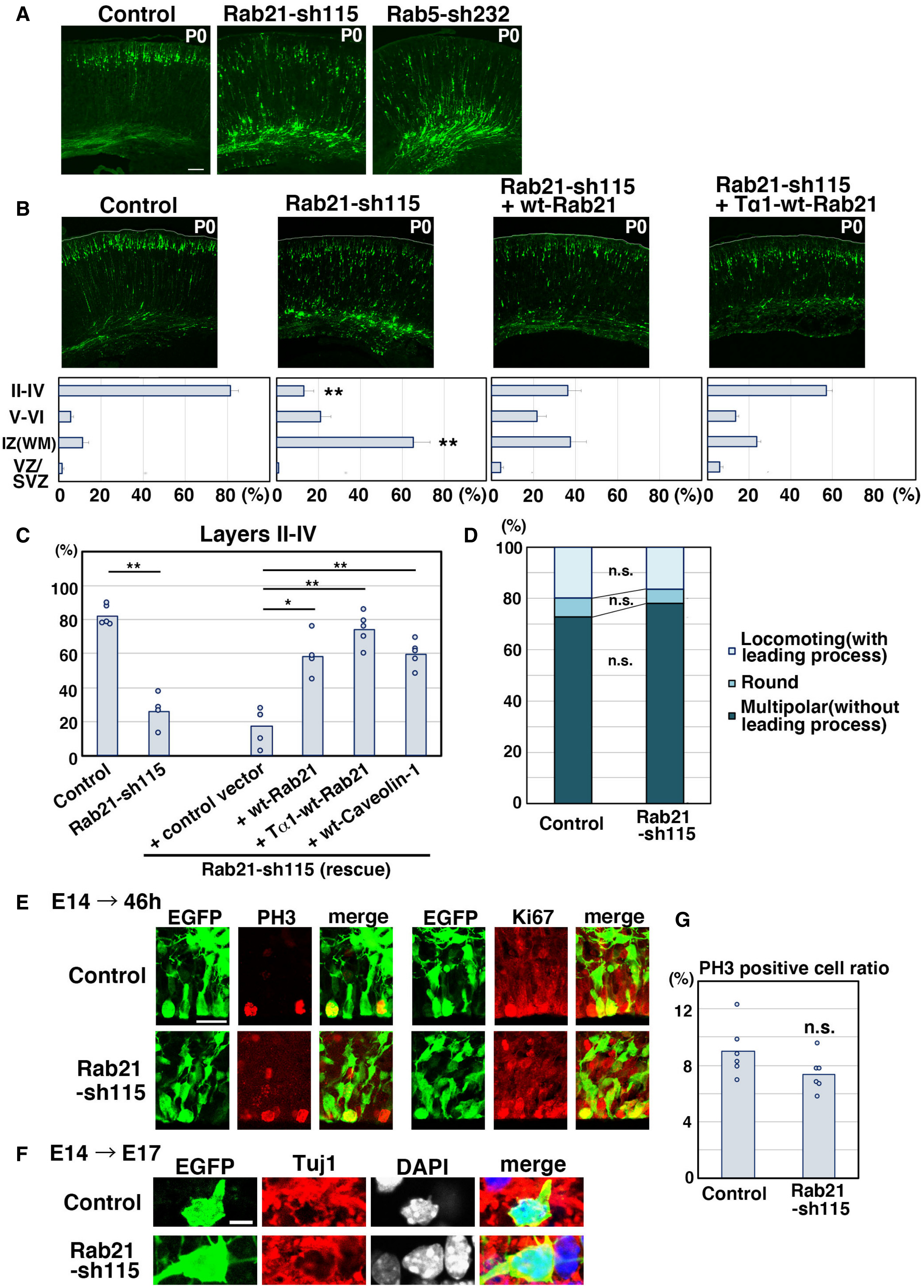

Figure EV2.

© 2023 The Authors

Figure EV3. Subcellular localization of Rab21 and caveolin-1 in primary cortical neurons and nonneuronal cultured cells.

A, B Primary cortical neurons from E15 cerebral cortices were transfected with EGFP-Rab5 (upper panels in A) or EGFP-Rab21 (lower panels in A) and incubated for 2 days *in vitro*. To maintain moderate expression levels, CMV promoter was used to express EGFP-Rab5 and EGFP-Rab21. Cells were immunostained with anti-EGFP (green) and anti-caveolin-1 (red) antibodies. White arrows in (A) indicate colocalization of EGFP-Rab21 and caveolin-1. The images are obtained with TCS-SP5 (Leica). The graphs in (B) show the estimation of colocalization, which was carried out by recording fluorescence intensities of EGFP-Rab5 or EGFP-Rab21 and caveolin-1 staining signals along the white line in the upper panels using Leica SP5 software. Red arrows indicate the colocalization of these proteins on the same peaks.

C–F Primary cortical neurons from E15 cerebral cortices were transfected with the indicated plasmids, incubated for 2 days *in vitro* and immunostained with the indicated antibodies. Lower panels in (C) and (E) are high magnification images indicated by white and blue rectangles in upper panels. Blue alone channels are shown in black and white images. The graphs in (D) and (F) show the Pearson's correlation coefficient between caveolin-1 (D) or Rab21 (F) and organelle markers. Each score represents the mean with the individual points. Caveolin-1—APPL1: $n = 38$ cells, Caveolin-1—calnexin: $n = 51$ cells, Rab21—calnexin: $n = 59$ cells, Rab21—KDEL: $n = 51$ cells.

G–J NIH3T3 or COS-1 cells were immunostained with the indicated antibodies. The images are obtained with high-resolution microscopy (Nikon). The graphs in (H) and (J) show the Pearson's correlation coefficient between caveolin-1 and Rab5 or Rab21 or between Rab21 and Rab5. Each score represents the mean with the individual points. Caveolin-1—Rab21: $n = 41$ cells (H) or 63 cells (J), Caveolin-1—Rab5: $n = 63$ cells (H) or 69 cells (J), Rab21—Rab5: 34 cells.

Data information: (D) Significance was determined by Mann–Whitney's *U* test ($P = 1.310E-14$, $^{**}P < 0.01$). (F) Significance was determined by one-way ANOVA with *post hoc* Tukey–Kramer. No significant difference was observed between Rab21—Calnexin and Rab21—KDEL, but compared to a negative control (Rab5—Lamp1 in Fig 1B), a significant difference was observed (less than the critical value at 1%: Rab21—calnexin, Rab21—KDEL [compared to a negative control]). (H, J) Significance between Caveolin-1—Rab21 and Caveolin-1—Rab5 was determined by Welch's t-test (H: $P = 5.795E-27$, J: $P = 7.367E-24$). $^{**}P < 0.01$. Scale bar: 3 μm in (A), 10 μm in (upper panels in C, E), 1 μm in (lower panels in C, E), 5 μm in (G, I).

© 2023 The Authors

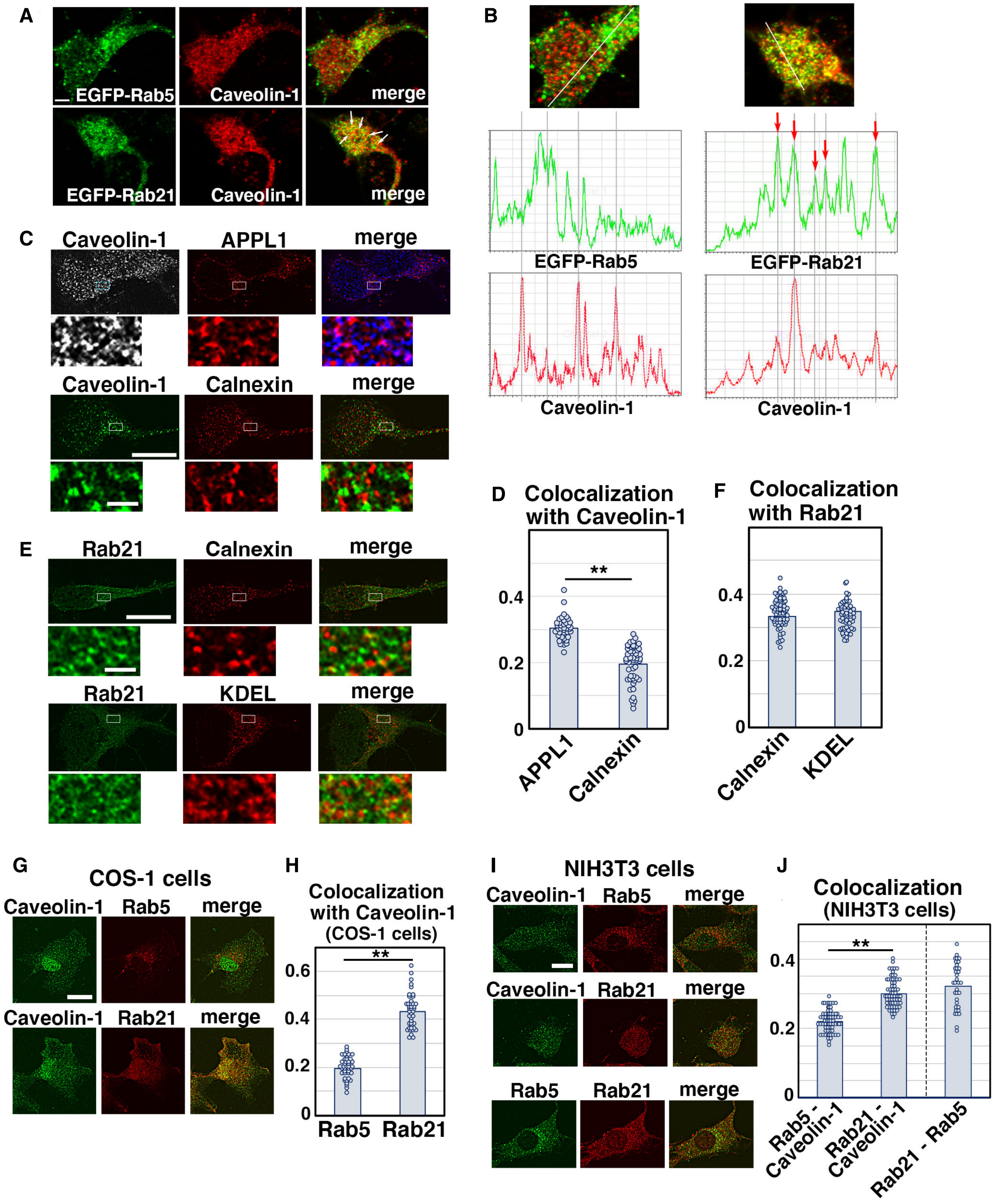

Figure EV3.

 © 2023 The Authors

Figure EV4. Rab21 is required for the uptake of LacCer, but not CD44 internalization.

A, B NIH3T3 fibroblasts were transfected with the indicated plasmids and treated with BODIPY-LacCer (LacCer) for 30 min before fixation. The images are obtained with high-resolution microscopy (Nikon). The graph in (B) shows the ratio of cells with perinuclear accumulation of LacCer. Each score represents the mean with the individual points. Control: $n = 4$ biological replicates, Rab21-sh115: $n = 4$ biological replicates, Rab5-sh232: $n = 4$ biological replicates.

C–E Primary cortical neurons from E15 cerebral cortices were incubated for 2 days *in vitro* and immunostained with the indicated antibodies. The images are obtained with high-resolution microscopy (Nikon). The graph in (E) shows the Pearson's correlation coefficient between Endophilin and Rab21 or caveolin-1 or between Rab21 and CD44. Each score represents the mean with the individual points. Caveolin-1—Endophilin: $n = 43$ cells, Rab21—Endophilin: $n = 53$ cells, Rab21—CD44: $n = 45$ cells.

F–H Primary cortical neurons from E15 cerebral cortices were transfected with the indicated plasmids plus pCAG-PM-mAG1, incubated for 2 days *in vitro* and subjected to CD44 antibody feeding assay. The graphs in (G) and (H) show the number of the internalized CD44-positive dots and its total fluorescence intensity per cell. Each score represents the mean with the individual points. Control: $n = 15$ cells (G and H), Rab21-sh115: $n = 12$ cells (G and H).

I, J Primary cortical neurons from E15 cerebral cortices were transfected with the indicated plasmids plus pCAG-EGFP, incubated for 8 days *in vitro* and stained with MAP2ab, a marker for dendrites. The graphs in (J) show the dendrite length, dendrite branch number and the number of primary dendrites. Each score represents the mean with the individual points. Control: $n = 31$ cells, Rab21-sh115: $n = 38$ cells, Rab5-sh232: $n = 31$ cells, Cav1-sh490: $n = 31$ cells.

K Cerebral cortex at P0, electroporated with the indicated plasmids plus pCAG-EGFP at E14.

L Frozen sections of E17 cerebral cortex immunostained with anti-Rab21 antibody and DAPI.

Data information: (B) Significance was determined by one-way ANOVA with *post hoc* Dunnett and Tukey–Kramer. **Less than the critical value at 1%, *less than the critical value at 5%, n.s.: no significant differences. (E) Significance was determined by one-way ANOVA with *post hoc* Tukey–Kramer. **Less than the critical value at 1%. (G, H) Significance was determined by Welch's *t*-test (G: $P = 0.1942$) or Mann–Whitney's *U* test (H: $P = 0.2225$). n.s.: no significant differences. (J) Significance compared to control was determined by one-way ANOVA with *post hoc* Dunnett. Significant difference was observed between control and Rab21-sh115 in the number of primary dendrites. *Less than the critical value at 5%, n.s.: no significant differences. Scale bars: 5 μm in (A, C, D, F), 10 μm in (I), 150 μm in (K), 100 μm in (L).

© 2023 The Authors

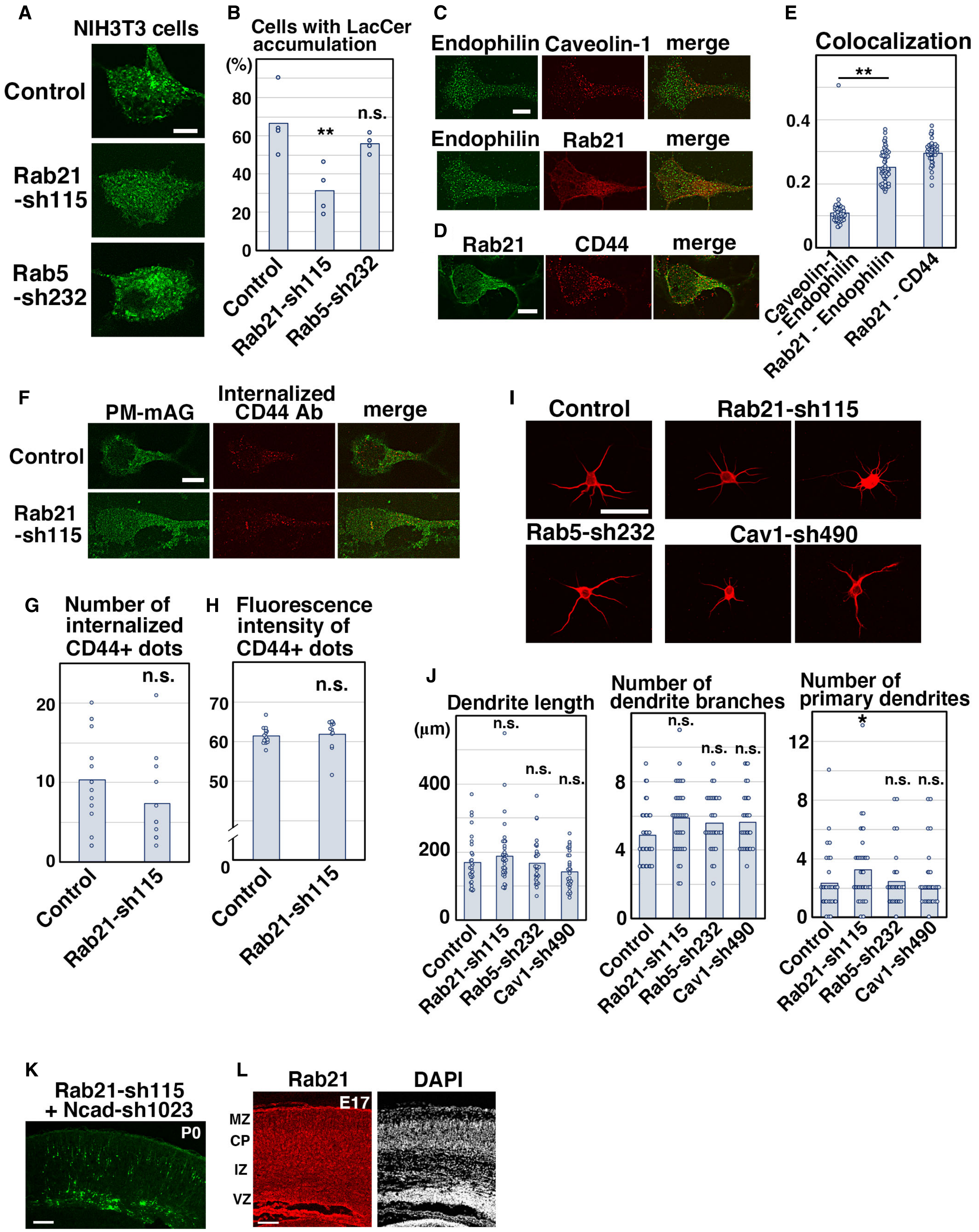

Figure EV4.

 © 2023 The Authors

Figure EV5. Knockdown of Rab21, but not Rab5, decrease the caveolin-1 protein levels.

A, B Primary cortical neurons from E15 cerebral cortices were transfected with the indicated plasmids plus pCAG-PM-mAG1 (green) and incubated for 2 days *in vitro*. Immunocytochemical analyses with anti-Rab21 or anti-Rab5 antibody (red) were performed. The graphs in (B) indicate the ratio of the Rab21 or Rab5 staining signals in the plasma membrane to total fluorescence intensities in each immature neuron. Each score represents the mean with the individual points. Control: $n = 29$ cells (left, Rab21) or 27 cells (right, Rab5), Cav1-sh490: $n = 24$ cells (left, Rab21) or 25 cells (right, Rab5).

C, D Primary cortical neurons from E15 cerebral cortices transfected with the indicated plasmids plus pCAG-EGFP and incubated for 2 days *in vitro*. Immunocytochemical analyses with anti-EGFP (green) and anti-caveolin-1 (red) antibodies (C, D) and quantitative PCR (E) were performed. White arrows in (C) indicate the Rab21-knockdown neurons with decreased caveolin-1 signals. The graph in (D) shows the fluorescence intensity of caveolin-1 per cell. Each score represents the mean with the individual points. Control: $n = 21$ cells, Rab21-sh115: $n = 22$ cells. Each score in (E) represents the ratio with the individual points.

F Primary cortical neurons from E15 cerebral cortices transfected with the indicated plasmids and incubated for 5 days *in vitro* (DIV). Immunoblot analyses of cell lysates with the indicated antibodies were performed. Long-term culture (5 DIV) did not affect the protein levels of caveolin-1 in the Rab5-sh232-transfected cortical neurons, similar to the 2 DIV neurons (Fig 6E), whereas the reduced caveolin-1 protein levels were still observed in the Rab21-sh115-transfected neurons at 5 DIV.

G, H Primary cortical neurons from E15 cerebral cortices transfected with the indicated plasmids, incubated for 1 DIV and treated with 160 nM Bafilomycin A1 (Baf A1) for 22 h. Immunoblot analyses of the cell lysates with the indicated antibodies were performed. The box-and-whisker plot in (H) shows the ratios of immunoblot band intensities of caveolin-1/beta-actin. Baf $A1^{-}$: $n = 11$ biological replicates, Baf $A1^{+}$: $n = 10$ biological replicates.

Data information: (B) Significance was determined by Mann–Whitney's *U* test (Rab21: $P = 0.2176$) or Student's *t*-test (Rab5: $P = 0.1599$), and no significant difference was observed. n.s.: no significant differences. (D) Significance was determined by Student's *t*-test ($P = 0.000004220$). $^{**}P < 0.01$. (E) Significance was determined by Welch's *t*-test ($n = 5$ biological replicates, $P = 0.7262$). n.s.: no significant differences. (H) In the box-and-whisker plots, the central band and the upper and lower sides of the boxes indicate the median and the upper and lower quartiles. The whiskers of the depicted boxplots go from the minimum to the lower quartile and from the upper quartile to the maximum. "x" indicates the average value. Significance was determined by Welch's t-test ($P = 0.03234$). $^{*}P < 0.05$. Scale bar: 1 μm in (A), 10 μm in (C).

© 2023 The Authors

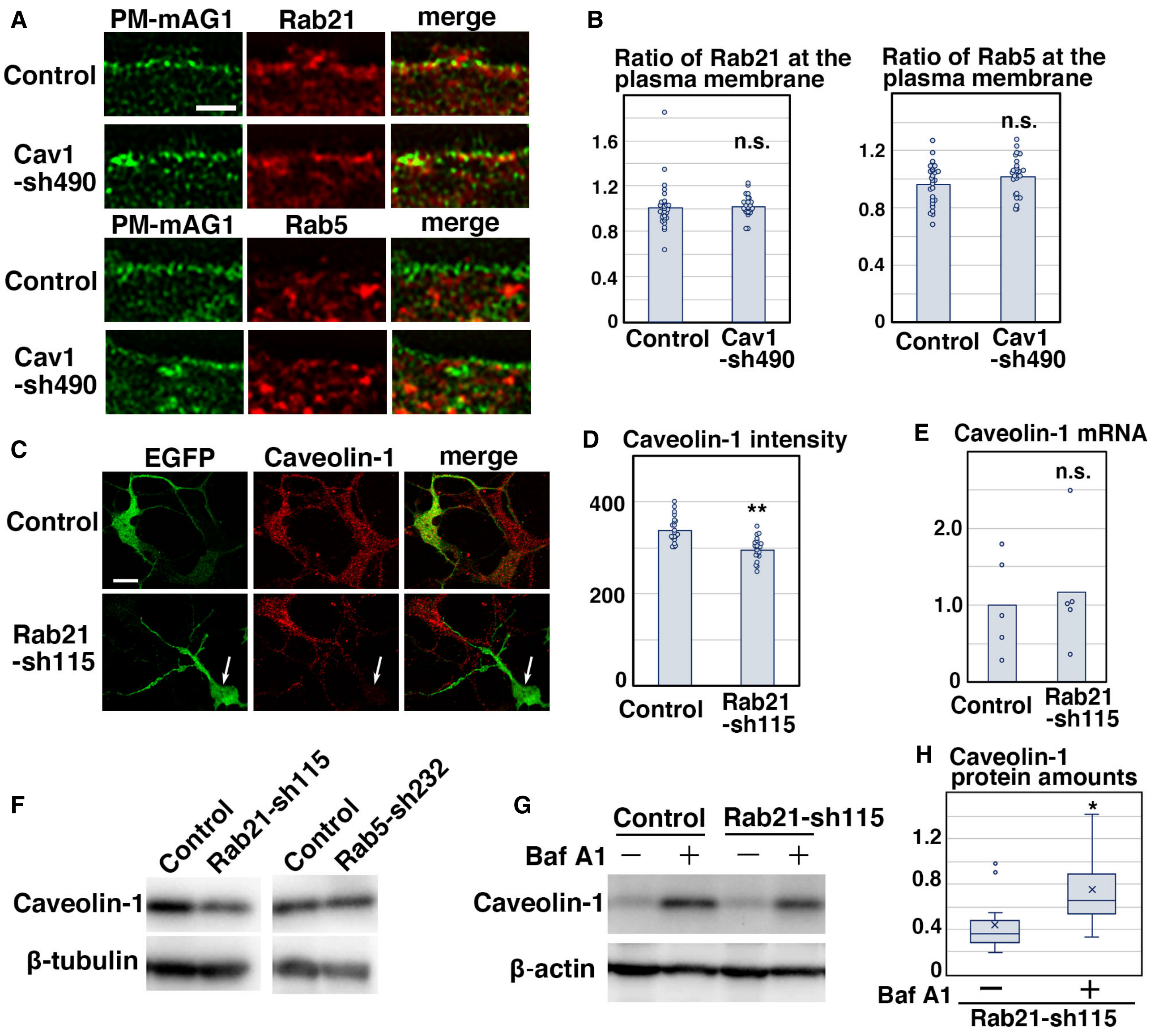

Figure EV5.

 © 2023 The Authors
