# Supplementary material for: Rab21 regulates caveolin‐1‐mediated endocytic trafficking to promote immature neurite pruning
Source: EMBO Rep. 2023 Jan 23;24(3):e54701. doi: 10.15252/embr.202254701 (PMC9986827; doi:10.15252/embr.202254701)
Supplement: Supplementary file 4 [file EMBR-24-e54701-s001.pdf]

EMBO reports

Rab21 regulates caveolin-1-mediated endocytic trafficking to promote immature neurite pruning

Mima Shikanai, Shiho Ito, Yoshiaki Nishimura, Remi Akagawa, Mitsunori Fukuda, Michisuke Yuzaki, Yo-ichi Nabeshima, and Takeshi Kawauchi
DOI: 10.15252/embr.202254701

Corresponding author(s): Takeshi Kawauchi (kawauchi.takeshi.i92@kyoto-u.jp)

Review Timeline:

Submission Date:	20th Jan 22
Editorial Decision:	18th Mar 22
Revision Received:	29th Aug 22
Editorial Decision:	13th Oct 22
Revision Received:	9th Dec 22
Accepted:	21st Dec 22

Editor: Martina Rembold

Transaction Report:

(Note: With the exception of the correction of typographical or spelling errors that could be a source of ambiguity, letters and reports are not edited. Depending on transfer agreements, referee reports obtained elsewhere may or may not be included in this compilation. Referee reports are anonymous unless the Referee chooses to sign their reports.)

Dear Dr. Kawauchi

Thank you for the submission of your research manuscript to our journal. I apologize for the delay in handling your manuscript, but we have only now received the full set of referee reports that is copied below.

As you will see, the referees acknowledge that the findings are potentially interesting. However, they also point out several conceptual and technical concerns and have a number of suggestions for how the study should be strengthened, and I think that all of them should be addressed.

Given these constructive comments, we would like to invite you to revise your manuscript with the understanding that the referee concerns (as detailed above and in their reports) must be fully addressed and their suggestions taken on board. Please address all referee concerns in a complete point-by-point response. Acceptance of the manuscript will depend on a positive outcome of a second round of review. It is EMBO reports policy to allow a single round of revision only and acceptance or rejection of the manuscript will therefore depend on the completeness of your responses included in the next, final version of the manuscript.

We realize that it is difficult to revise to a specific deadline. In the interest of protecting the conceptual advance provided by the work, we recommend a revision within 3 months (June 18, 2022). Please discuss the revision progress ahead of this time with the editor if you require more time to complete the revisions.

You can either publish the study as a short report or as a full article. For short reports, the revised manuscript should not exceed 27,000 characters (including spaces but excluding materials & methods and references) and 5 main plus 5 expanded view figures. The results and discussion sections must further be combined, which will help to shorten the manuscript text by eliminating some redundancy that is inevitable when discussing the same experiments twice. For a normal article there are no length limitations, but it should have more than 5 main figures and the results and discussion sections must be separate. In both cases, the entire materials and methods must be included in the main manuscript file.

***IMPORTANT NOTE:
We perform an initial quality control of all revised manuscripts before re-review. Your manuscript will FAIL this control and the handling will be DELAYED if the following APPLIES:

1) A data availability section providing access to data deposited in public databases is missing. If you have not deposited any data, please add a sentence to the data availability section that explains that.

2) Your manuscript contains statistics and error bars based on n=2. Please use scatter blots in these cases. No statistics should be calculated if n=2. ***

When submitting your revised manuscript, please carefully review the instructions that follow below. Failure to include requested items will delay the evaluation of your revision.

When submitting your revised manuscript, we will require:

1) a .docx formatted version of the manuscript text (including legends for main figures, EV figures and tables). Please make sure that the changes are highlighted to be clearly visible.

2) individual production quality figure files as .eps, .tif, .jpg (one file per figure).
Please download our Figure Preparation Guidelines (figure preparation pdf) from our Author Guidelines pages https://www.embopress.org/page/journal/14693178/authorguide for more info on how to prepare your figures.

3) a .docx formatted letter INCLUDING the reviewers' reports and your detailed point-by-point responses to their comments. As part of the EMBO Press transparent editorial process, the point-by-point response is part of the Review Process File (RPF), which will be published alongside your paper.

4) a complete author checklist, which you can download from our author guidelines (). Please insert information in the checklist that is also reflected in the manuscript. The completed author checklist will also be part of the RPF.

5) Please note that all corresponding authors are required to supply an ORCID ID for their name upon submission of a revised manuscript (). Please find instructions on how to link your ORCID ID to your account in our manuscript tracking system in our Author guidelines

()

6) We replaced Supplementary Information with Expanded View (EV) Figures and Tables that are collapsible/expandable online. A maximum of 5 EV Figures can be typeset. EV Figures should be cited as 'Figure EV1, Figure EV2" etc... in the text and their respective legends should be included in the main text after the legends of regular figures.

- For the figures that you do NOT wish to display as Expanded View figures, they should be bundled together with their legends in a single PDF file called *Appendix*, which should start with a short Table of Content. Appendix figures should be referred to in the main text as: "Appendix Figure S1, Appendix Figure S2" etc. See detailed instructions regarding expanded view here:

- Additional Tables/Datasets should be labeled and referred to as Table EV1, Dataset EV1, etc. Legends have to be provided in a separate tab in case of .xls files. Alternatively, the legend can be supplied as a separate text file (README) and zipped together with the Table/Dataset file.

7) Please note that a Data Availability section at the end of Materials and Methods is now mandatory. In case you have no data that requires deposition in a public database, please state so instead of refereeing to the database.
See also < https://www.embopress.org/page/journal/14693178/authorguide#dataavailability>).
Please note that the Data Availability Section is restricted to new primary data that are part of this study.

8) Figure legends and data quantification:
The following points must be specified in each figure legend:
- the name of the statistical test used to generate error bars and P values,
- the number (n) of independent experiments (please specify technical or biological replicates) underlying each data point,
- the nature of the bars and error bars (s.d., s.e.m.)
- If the data are obtained from n {less than or equal to} 2, use scatter blots showing the individual data points.
Discussion of statistical methodology can be reported in the materials and methods section, but figure legends should contain a basic description of n, P and the test applied.
See also the guidelines for figure legend preparation:
https://www.embopress.org/page/journal/14693178/authorguide#figureformat
- Please also include scale bars in all microscopy images.

9) We would also encourage you to include the source data for figure panels that show essential data. Numerical data should be provided as individual .xls or .csv files (including a tab describing the data). For blots or microscopy, uncropped images should be submitted (using a zip archive if multiple images need to be supplied for one panel). Additional information on source data and instruction on how to label the files are available .

10) Our journal encourages inclusion of *data citations in the reference list* to directly cite datasets that were re-used and obtained from public databases. Data citations in the article text are distinct from normal bibliographical citations and should directly link to the database records from which the data can be accessed. In the main text, data citations are formatted as follows: "Data ref: Smith et al, 2001" or "Data ref: NCBI Sequence Read Archive PRJNA342805, 2017". In the Reference list, data citations must be labeled with "[DATASET]". A data reference must provide the database name, accession number/identifiers and a resolvable link to the landing page from which the data can be accessed at the end of the reference. Further instructions are available at .

11) As part of the EMBO publication's Transparent Editorial Process, EMBO reports publishes online a Review Process File to accompany accepted manuscripts. This File will be published in conjunction with your paper and will include the referee reports, your point-by-point response and all pertinent correspondence relating to the manuscript.

You are able to opt out of this by letting the editorial office know (emboreports@embo.org). If you do opt out, the Review Process File link will point to the following statement: "No Review Process File is available with this article, as the authors have chosen not to make the review process public in this case."

We would also welcome the submission of cover suggestions, or motifs to be used by our Graphics Illustrator in designing a cover.

I look forward to seeing a revised form of your manuscript when it is ready and please let me know if you have questions or comments regarding the revision.

Yours sincerely,

Martina Rembold, PhD
Senior Editor
EMBO reports

****

Referee #1:

Manuscript by Shikanai et al.

In this manuscript, the authors follow up on their previous publications associating caveolin-1 and clathrin-independent endocytosis in neuronal maturation. Here they found that this process occurs through Rab21 but not Rab5 at the endosomal level. This study is interesting as it reports new findings about Rab21. There are however a few controls that need to be performed to better support the conclusions made by the authors.

As a first general comment, this manuscript relies mostly on cell imaging that suffers from a general lack of quantification and statistical validation. Colocalization should be assessed by the Pearson's correlation coefficient. This index is sometimes mentioned in the text but it should be also displayed on the corresponding panels. Another point that appears weak here is the nature of the intracellular organelle where Rab21 and Cav1 may colocalize.

Fig 1B-C: Quantification is missing. I found the data not very convincing in terms of efficiency of Tf uptake inhibition. Tf upake should be quantified with standard assays such as FACS or biotin assays. We need to know where Tf is located under Rab21 and Rab5 inhibition. Colocalization with markets of the perinuclear compartment and the early endosome should be provided.

Fig 2: Quantification and Pearson's colocalization coefficient should be displayed for all colocalization immunofluorescence. The caveolin-1 staining appears quite unconventional as it is not dotty and rather diffuse typical of ER staining. Same for Rab21 that has indeed already been reported as localized in the ER. The authors should therefore rule out a staining at the ER with ER markers. In B same magnification should be shown for Rab5. The localization of Rab5 and Rab21 should be done under Cav-1 inhibition.
The authors have quantified the effect of Cav-1 inhibition in Fig EV4 C but the corresponding IF panels are not shown. They should be shown.

The role of caveolae in endocytosis has been revisited recently and appears to be minor. There seem to be a confusion here as for the role of caveolae vs caveolin endocytosis. Whereas caveolae may indeed contribute to the uptake of LacCer and CtxB, these markers are not the best reporters of Cav-1 mediated endocytosis. To follow Cav-1 mediated uptake that is the so-called GCLIC/GEEC pathway, the authors ought to examine cargos and regulators of this pathway such as CD44 or GPI and endophilin, respectively (DOI: 10.1371/journal.pbio.1001832). Rab21 effect should therefore be tested on CD44 or GPI uptake as already done (PMID: 30610016).

Fig 3: A-B: Pearson's index should be shown. Corresponding IF panels for D-G should be included. Cav-1 and Rab5 KO should be shown for comparison.

Fig 4A: Panel 4A and EV6 A are quite different. Cav1 staining after Rab21-sh115 seems to be localized to intracellular structures in 4A. Are these structures the lysosomes that they mention later? Lysotracker should be used to document this point. Although Rab7 reversion is interesting, direct lysosomal inhibition should be done to test this hypothesis.
I do not understand the point made by the authors to explain the increase of Cav1 levels through upregulated endocytosis since they show here that Cav1 was not dependent on Rab5.

Fig 5: Cav1 rescue of Rab21 on neurite pruning phenotype should be correlated with a rescued effect on N-cadherin internalization.

Referee #2:

This manuscript investigates the role of Rab21 in early neuronal development in mouse cortex. Rab21 is in the same class of Rabs as Rab5 which is better studied. The two rabs are considered to be quite similar in their cellular functions. This work builds on previous work from this group investigating Rab5 and Rab7 in neuronal migration in developing mouse cortex. The authors make the surprising discovery that Rab5 and Rab21 knockdown result in different early cortical phenotypes, where shRab21 causes supernumerary dendrites prior to initiating migration. This phenotype is interpreted as a failure to prune the multiple processes of multipolar cells in order to take on the migratory bipolar morphology. This is quite interesting and not commonly investigated. The authors propose a model based on their data in which Ncadherin requires caveolin to be removed from the surface which is hypothesized to cause the pruning. There are strong data from in utero electroporation as well as primary neuronal cultures to show that shRab21 (but not Rab5) regulates caveolin levels and Ncad surface abundance. The strengths of this manuscript are the in vivo experiments in combination with cell biological assays carried out in primary neurons. In addition, the fact that shRab21 phenotypes can be rescued by shNcad or caveolin overexpression makes a strong case supporting their

hypothesis.

Detailed comments:
1) please show individual data points for all bar graphs.
2) The link with Ncad trafficking is not completely brought to a close. Ncad is shown to accumulate in early endosomes, but where is it normally going after endocytosis? Is it recycling or degrading? shRab21 leads to less caveolin but more Ncad on surface even though Ncad endocytosis requires caveolin. Are they endocytosing together? If so, where are the segregating? Why is caveolin low in shRab21? Is this a trafficking problem?
3) Are dendrites defective in cultured neurons after shRab21?
4) I find the figures that use pink and red hard to see. The pink and red are too similar to easily distinguish. Could you put the Pearson co-efficients next to the pictures in the figures?

Referee #3:

Shikanai et.al examines the role of the small GTPases, Rab5 and Rab21, in different types of endocytosis. They conclude that these GTPases differentially regulate clathrin- and caveolin-mediated endocytosis, and the regulation of caveolin-mediated endocytosis by Rab21 is required for neurite pruning.

Overall, the paper is interesting but there are conceptual and technical issues that needs to be addressed:

Conceptual:

1. One of the main claims of the paper is that Rab5 and 21 do not colocalize, which is in disagreement with previous studies (Egami & Araki, 2009, Simpson et al., 2004).
Can this lack of colocalization neurons specific ? In case the authors believe it is a general phenomenon they should show it in other cells.

2. Same goes for the role of Rab5 in caveolin-mediated endocytosis that demonstrated in non-neuronal cells (Ariotti & Parton, 2013, Hagiwara et al., 2009, Hayer et al., 2010, Pelkmans et al., 2004).

3. Rescue of the Rab21 KD by caveolin OE. I am somehow surprised by these results. Does It means that the main role of Rab21 is to control the levels of caveolin and not localization ?

Technical:
1. Overall, the colocalization experiments are not of the highest quality but they are largely convincing. In 2C both Rabs doesn't seem to strongly localized with the PM.

2. How the authors can be sure that Rab21 and caveolin are present in the same vesicular compartment ?

3. looking at the EV3D it does not look like pruning defect to me. Indeed, in the control there is elimination of immature neurites. But I am not sure the same neurites hang around in the Rab21-sh for the entire time. Looks to me that some are eliminated and new ones are formed.

4. The internalization experiments in Fig2 and EV4 require rescue experiments.

5. Fig 3C only 20% effect - is this minor effect can drive the biology?

6. Fig 5A not really resembling the phenotype in 1D

Minor:
1. The term sticky neurons is no clear

2. Fig 3D-G please interest the Y axis.

Point-by-point responses to the reviewers' comments
MS. No.: EMBOR-2022-54701V1

Our point-by-point response to all the issues raised by the reviewers is provided below. For your convenience, the editor's and the reviewers' comments are shown below in italic and bold.

IMPORTANT NOTE:
1) A data availability section providing access to data deposited in public databases is missing. If you have not deposited any data, please add a sentence to the data availability section that explains that.

[Response]
The submitted manuscript does not include sequence data nor big data, and therefore there is no data that should be deposited in public databases.
We have now added a "Data availability" section, where we state "This study includes no data deposited in public repositories".

2) Your manuscript contains statistics and error bars based on n=2. Please use scatter blots in these cases. No statistics should be calculated if n=2. ***

[Response]
The revised manuscript does not contain statistics based on n=2.
In the revised manuscript, we have replaced the graphs with the bar graphs showing the individual data points or box-and-whisker plots.

****
Referee #1:

Manuscript by Shikanai et al.

In this manuscript, the authors follow up on their previous publications associating caveolin-1 and clathrin-independent endocytosis in neuronal maturation. Here they found that this process occurs through Rab21 but not Rab5 at the endosomal level. This study is interesting as it reports new findings about Rab21. There are however a few controls that need to be performed to better support the conclusions made by the authors.

As a first general comment, this manuscript relies mostly on cell imaging that suffers from a general lack of quantification and statistical validation. Colocalization should be assessed by the Pearson's correlation coefficient. This index is sometimes mentioned in the text but it should be also displayed on the corresponding panels. Another point that appears weak here is the nature of the intracellular organelle where Rab21 and Cav1 may colocalize.

[Response-1]

According to the reviewer's suggestion, we quantified the colocalization data by using Pearson's correlation coefficient. The results are shown in Figures. 1B, 1G, 3C, 3G, 3I, 6I, EV1C, EV4D, EV4F, EV5B, EV5D, EV5I and EV6F.

Regarding the second point, we performed detailed localization analyses of Rab21 and caveolin-1 in cortical neurons. We show new data that Rab21 is mainly localized in the plasma membrane and early endosomes (Pearson's correlation coefficients between Rab21 and the marker proteins are 0.528 and 0.558, respectively) (Fig. 3C and Fig. EV1C). Caveolin-1 is also localized in the plasma membrane (Pearson's correlation coefficient = 0.405), and there is some caveolin-1 localized in the early endosomes to a lesser extent (Pearson's correlation coefficient = 0.304) (Fig. 3C and Fig. EV4F). Caveolin-1 is barely localized with the Rab11-positive recycling endosomes, Rab6-positive Golgi apparatus and calnexin-positive ER (Pearson's correlation coefficient of caveolin-1 and Rab11 or Rab6 or calnexin is less than 0.2), as shown below (Supplemental Figure for the reviewers) (see also Fig. EV4F).

Given that both Rab21 and caveolin-1 are localized at the plasma membrane, we examined the colocalization of Rab21 and caveolin-1 in the transfected Plasma Membrane-targeted monomeric Azami Green 1 (PM-mAG1)-positive regions. We found that these proteins are colocalized at the plasma membrane (Pearson's correlation coefficient = 0.620). Rab21 and caveolin-1 may also colocalize at the early endosomes to some extent, but Rab21, caveolin-1 and EEA1 (or APPL1) cannot be simultaneously

stained due to the overlapping antibody species. We were therefore unable to examine this.

These results are shown in Fig. 3B-C, EV1B-1C and Fig. EV4C-F.

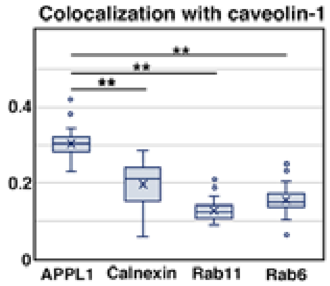

Supplemental Figure for the reviewers

Fig 1B-C: Quantification is missing. I found the data not very convincing in terms of efficiency of Tf uptake inhibition. Tf upake should be quantified with standard assays such as FACS or biotin assays. We need to know where Tf is located under Rab21 and Rab5 inhibition. Colocalization with markets of the perinuclear compartment and the early endosome should be provided.

[Response-2]

To address this issue, we performed a surface biotinylation assay after the transferrin (Tf) uptake. Knockdown of Rab21 did not increase the cell surface levels of Tf, suggesting that Rab21 is not involved in the internalization of Tf. The results are shown in Fig. EV1G-H

We next analyzed subcellular localization of Tf with the Rab21 and Rab5 knockdown. Although knockdown of Rab5 results in only a small increase of the surface Tf, several dot-like subcellular localizations of Tf was observed. The Tf-positive dots were partially colocalized with early endosomal marker, APPL1. However, the efficiency of colocalization is not very high, probably because APPL1 is a Rab5 effector protein and requires Rab5 to translocate to the early endosomes. Importantly, compared with control and Rab21-knockdown neurons, where the Tf was found in the Rab11-positive recycling endosomes, the ratio of the Tf localized at the Rab11-positive recycling endosomes is significantly low in Rab5-knockdown neurons. This suggests that Rab5, but not Rab21, is required for the trafficking of Tf to the recycling endosomes. These new data are shown in Fig. 1E-G.

Fig 2: Quantification and Pearson's colocalization coefficient should be displayed for all colocalization immunofluorescence. The caveolin-1 staining appears quite unconventional as it is not dotty and rather diffuse typical of ER staining. Same for Rab21 that has indeed already been reported as localized in the ER. The authors should therefore rule out a staining at the ER with ER markers. In B same magnification should be shown for Rab5. The localization of Rab5 and Rab21 should be done under Cav-1 inhibition.

The authors have quantified the effect of Cav-1 inhibition in Fig EV4 C but the corresponding IF panels are not shown. They should be shown.

[Response-3]

According to the reviewer's suggestion, we added the Pearson's correlation coefficient data, which clearly show that Rab21 preferentially colocalizes with caveolin-1, compared to Rab5. The results are shown in Fig. 3C and 3G.

We also examined the localization of Rab21 and caveolin-1 in the ER. Although a small amount of Rab21 is colocalized with calnexin, an ER marker, caveolin-1 does not localize in the ER. Therefore, Rab21-mediated regulation of caveolin-1 does not occur in the ER. The results are shown in Fig. EV4C-F

Regarding the original Fig. 2B and Fig. EV4C, we have now added images of Rab5 and caveolin-1 staining (Fig. 3A) and CTxB localization with caveolin-1 knockdown (Fig. 4C). The localization data of endogenous Rab21 and Rab5 are now shown in the revised version of Fig. 1A-B. The remaining panels from original Fig. 2A have been moved to Expanded View Figure (Fig. EV4A).

Lastly, we analyzed the effects of caveolin-1 knockdown on the localization of Rab5 and Rab21. Our quantitative analyses indicate that knockdown of caveolin-1 does not affect the plasma membrane localization of Rab21 and Rab5, suggesting that Rab21 is an upstream regulator for caveolin-1. The results are shown in Fig. EV5M-N.

The role of caveolae in endocytosis has been revisited recently and appears to be minor. There seem to be a confusion here as for the role of caveolae vs caveolin endocytosis. Whereas caveolae may indeed contribute to the uptake of LacCer and CtxB, these markers are not the best reporters of Cav-1 mediated endocytosis. To follow Cav-1 mediated uptake that is the so-called GCLIC/GEEC pathway, the

authors ought to examine cargos and regulators of this pathway such as CD44 or GPI and endophilin, respectively (DOI: 10.1371/journal.pbio.1001832). Rab21 effect should therefore be tested on CD44 or GPI uptake as already done (PMID: 30610016).

[Response-4]

To address this issue, we first examined the localization of Rab21 and CD44 or endophilin. In primary cortical neurons, Rab21 and endophilin are hardly colocalized. Although a little colocalization between Rab21 and CD44 was observed, previously reported CD44-positive tubular structures (EMBO Rep, 20, e47192, 2019) are barely observed. In addition, unlike non-neuronal cells, knockdown of Rab21 or Rab5 does not affect the uptake of anti-CD44 antibody in primary cortical neurons. We do not understand the reason underlying the difference in the contribution of the CLIC/GEEC pathway between primary cortical neurons and non-neuronal cells, but we believe that this is beyond the scope of this manuscript, although it is an interesting phenomenon for the future studies. The results are shown in Fig. EV5G-I and EV5J-L.

Fig 3: A-B: Pearson's index should be shown. Corresponding IF panels for D-G should be included. Cav-1 and Rab5 KO should be shown for comparison.

[Response-5]

The Pearson's correlation coefficient between Rab21 and N-cadherin is 0.346 (Fig. EV6E-F). This moderate value may be due to the fact that N-cadherin is transported through many endosomes/organelles, whereas Rab21 is mainly localized in the plasma membrane and early endosomes, and thus colocalization of Rab21 and N-cadherin may occur transiently during the trafficking of N-cadherin.

We have previously published the knockdown of caveolin-1 and Rab5 (Neuron, 67, 588-602, 2010; iScience, 7, 53-67, 2018). Knockdown of caveolin-1 increases the cell surface levels of N-cadherin, similar to Rab21 knockdown. However, unlike Rab21 knockdown, caveolin-1 knockdown does not affect the early endosomal N-cadherin (iScience, 7, 53-67, 2018). In contrast, Rab5 knockdown also increases the cell surface N-cadherin, but Rab5-mediated regulation of Rab5 is involved in the radial fiber-dependent locomotion mode of neuronal migration (Neuron, 67, 588-602, 2010), which is a different maturation step of immature neurite pruning. Thus, both Rab5 and

Rab21 regulate N-cadherin internalization, but act in distinct steps of neuronal maturation. These points are mentioned in the Discussion as shown below.

"Interestingly, a major cargo molecule of both Rab5 and Rab21 to promote neuronal migration and maturation, respectively, is N-cadherin. This suggests that the precise spatio-temporal regulation of N-cadherin endocytic trafficking is crucial for multiple steps of cortical development; Rab5- and Rab21-mediated regulation of N-cadherin is required for the radial fiber-dependent neuronal migration and immature neurite pruning, respectively, which are distinct steps of neuronal maturation in the developing cerebral cortex.

At subcellular levels, clathrin-mediated endocytosis occurs in the non-raft membrane domain, whereas caveolin-1 is mainly localized in the cholesterol- and ganglioside-rich detergent-insoluble membrane domain (Nakashima *et al*, 2007; Parton, 1994). Consistently, caveolin-1 is colocalized with GD3 ganglioside, but not CD71, a non-raft marker (Macdonald & Pike, 2005; Mayle *et al*, 2012), in cortical neurons (Shikanai *et al.*, 2018). Because our previous report indicates that N-cadherin is localized in both GD3-positive and GD3-negative membrane domains (Shikanai *et al.*, 2018), Rab5 and Rab21 may regulate different populations of N-cadherin on the specific plasma membrane domains."

Fig 4A: Panel 4A and EV6 A are quite different. Cav1 staining after Rab21-sh115 seems to be localized to intracellular structures in 4A. Are these structures the lysosomes that they mention later? Lysotracker should be used to document this point. Although Rab7 reversion is interesting, direct lysosomal inhibition should be done to test this hypothesis.
I do not understand the point made by the authors to explain the increase of Cav1 levels through upregulated endocytosis since they show here that Cav1 was not dependent on Rab5.

[Response-6]

As the reviewer pointed out, we observed two phenotypes among the Rab21-knockdown neurons. One shows defects in the plasma membrane localization of caveolin-1 (Fig. 4A in the original manuscript) and the other exhibits a decrease of caveolin-1 staining signals (Fig. EV6A in the original manuscript). Our additional experiments suggest that Rab21 is required for proper localization of caveolin-1 in the

plasma membrane and that in absence of Rab21, mislocalized caveolin-1 is transported to the lysosomes. Thus, the two phenotypes observed in the Rab21-knockdown neurons result from the same abnormality.

We first examined the relationship between the mislocalized caveolin-1 and lysosomes in Rab21-knockdown neurons. We used Lamp1 as a lysosome marker, because the staining signals of Lamp1 was sharp, compared to the lysotracker. Because colocalization of caveolin-1 and Lamp1 is barely observed, we treated primary cortical neurons with Bafilomycin A1, a lysosome inhibitor, for 6 hours to detect the caveolin-1 transported to the lysosomes. Colocalization of caveolin-1 and Lamp1 is increased in the Rab21-knockdown neurons, compared to control neurons, suggesting that the mislocalized caveolin-1 is transported to the lysosomes at least in part. Other caveolin-1-positive intracellular structures including the early endosomes are also observed.

We then analyzed total fluorescence intensities of caveolin-1 per neuron, and found that overall expression levels of caveolin-1 were decreased in the Rab21-knockdown, consistent with the immunoblot results. Similar to the Rab7 inhibition, treatment with Bafilomycin A1 increased the protein levels of caveolin-1. However, treatment with Bafilomycin A1 for 22h also increased the protein levels of caveolin-1 in control neurons, making it hard to see the rescue effects.

These results suggest that Rab21 regulates the plasma membrane localization of caveolin-1 and that knockdown of Rab21 decreases the caveolin-1 at the plasma membrane. The mislocalized caveolin-1 is largely transported to the lysosomes and degraded, resulting in the reduction of the protein levels of caveolin-1. Treatment with Bafilomycin A1 (or inhibition of Rab7-dependent lysosomal trafficking pathways) restored the protein levels of caveolin-1, but caveolin-1 is still mislocalized at the lysosomes in the cells treated with Bafilomycin A1.

We have added the new data (Fig. 6H-I and Fig. EV7B and EV7E-F) and the corresponding explanation for our current model in the Discussion as shown below.

"We show that Rab21 and caveolin-1 are highly colocalized at the plasma membrane and that knockdown of Rab21 decreases the membrane localization of caveolin-1. The mislocalized caveolin-1 in Rab21-knockdown neurons is transported to the lysosomes and degraded. As a result, Rab21 knockdown results in the reduction of caveolin-1 protein levels."

Fig 5: Cav1 rescue of Rab21 on neurite pruning phenotype should be correlated with a rescued effect on N-cadherin internalization.

[Response-7]

Both overexpression of caveolin-1 or weak knockdown of N-cadherin restores the immature neurite pruning in Rab21-knockdown neurons. However, some Rab21-knockdown neurons with a weak knockdown of N-cadherin exhibit branched leading processes. This is because N-cadherin is required for the formation of the leading process, as well as immature neurites (Neuron, 67, 588-602, 2010; iScience, 7, 53-67, 2018).

During the multipolar-to-bipolar transition, N-cadherin needs to be internalized to retract the immature neurites, but N-cadherin is also required for the leading process formation. Thus Rab21- and caveolin-1-mediated N-cadherin endocytosis occurs in the immature neurites, while N-cadherin is transported to the plasma membrane of the leading process to elongate it. This dual function of N-cadherin may cause confusion with the two rescue experiments. Weak knockdown of N-cadherin restores the abnormally increased N-cadherin on the cell surface of the immature neurites, but as the leading process still lacks N-cadherin, this may cause abnormal morphologies of the leading processes.

These results are presented in the main text of the revised manuscript.

"While Rab21-sh115-electroporated neurons abnormally extended many immature neurites as described above, co-electroporation with Rab21-sh115 and Ncad-sh1023, which were expected to restore the increased cell surface levels of N-cadherin, rescued the pruning defects in immature neurites (Fig. EV6A-B), similar to the case of caveolin-1 knockdown (Shikanai *et al.*, 2018). However, some neurons that were cotransfected with Rab21-sh115 and Ncad-sh1023 exhibited a leading process with abnormal morphology, likely due to the requirement of N-cadherin for the leading process elongation (Shikanai *et al.*, 2018)."

Referee #2:

This manuscript investigates the role of Rab21 in early neuronal development in mouse cortex. Rab21 is in the same class of Rabs as Rab5 which is better studied. The

two rabs are considered to be quite similar in their cellular functions. This work builds on previous work from this group investigating Rab5 and Rab7 in neuronal migration in developing mouse cortex. The authors make the surprising discovery that Rab5 and Rab21 knockdown result in different early cortical phenotypes, where shRab21 causes supernumerary dendrites prior to initiating migration. This phenotype is interpreted as a failure to prune the multiple processes of multipolar cells in order to take on the migratory bipolar morphology. This is quite interesting and not commonly investigated. The authors propose a model based on their data in which Ncadherin requires caveolin to be removed from the surface which is hypothesized to cause the pruning. There are strong data from in utero electroporation as well as primary neuronal cultures to show that shRab21 (but not Rab5) regulates caveolin levels and Ncad surface abundance. The strengths of this manuscript are the in vivo experiments in combination with cell biological assays carried out in primary neurons. In addition, the fact that shRab21 phenotypes can be rescued by shNcad or caveolin overexpression makes a strong case supporting their hypothesis.

Detailed comments:
1) please show individual data points for all bar graphs.

[Response-8]

As suggested by the reviewer, we added the individual data points for the bar graphs. For the experiments with sample numbers greater than 10, the individual points overlap and are difficult to distinguish, so we applied box-and-whisker plots to show the dispersion.

2) The link with Ncad trafficking is not completely brought to a close. Ncad is shown to accumulate in early endosomes, but where is it normally going after endocytosis? Is it recycling or degrading? shRab21 leads to less caveolin but more Ncad on surface even though Ncad endocytosis requires caveolin. Are they endocytosing together? If so, where are the segregating? Why is caveolin low in shRab21? Is this a trafficking problem?

[Response-9]

To address this issue, we analyzed the subcellular localization of N-cadherin. Consistent with our previous report (Neuron, 67, 588-602, 2010), N-cadherin is preferentially transported to the Rab11-positive recycling endosomes after internalization to the early endosomes. Some low levels of N-cadherin were also observed in Rab7-positive lysosomal degradation pathways. The Pearson's correlation coefficients between N-cadherin and APPL1 (early endosomes) or Rab11 (recycling endosomes) or Rab7 (late endosomes) are 0.334, 0.320 and 0.271, respectively (Significance was observed between APPL1 or Rab11 and Rab7). The results are shown in Fig. EV6E-F.

We have previously reported that both N-cadherin and caveolin-1 are localized in the GD3 ganglioside-positive plasma membrane (iScience, 7, 53-67, 2018). Our new data show that caveolin-1 localization is decreased in the early endosomes, compared to the plasma membrane, and caveolin-1 is barely observed in the Rab11-positive recycling endosomes (Pearson's correlation coefficients between caveolin-1 and PM-mAG1 (plasma membrane) or APPL1 (early endosomes) or Rab11 (recycling endosomes) are 0.405, 0.304 and 0.127, respectively). Considering that N-cadherin localizes in both APPL1-positive early endosomes and Rab11-positive early endosomes (Pearson's correlation coefficient are 0.334 and 0.320, respectively), these data suggest that caveolin-1 dissociates from the endocytic pathways during trafficking from the early endosomes to the recycling endosomes, whereas N-cadherin continues to be transported to the recycling endosomes. The results are shown in Fig. EV4F and Supplemental Figure for the reviewers (please see our Response-1).

Rab21 is colocalized with caveolin-1 in the plasma membrane and promotes its plasma membrane localization because knockdown of Rab21 decreases caveolin-1 in the plasma membrane. In the Rab21-knockdown cells, the mislocalized caveolin-1 is mainly transported to the lysosomes and degraded, resulting in the reduction of caveolin-1 protein levels. This is our current model for why caveolin-1 is low in the Rab21-knockdown neurons. The results are shown in Figure 6.

3) Are dendrites defective in cultured neurons after shRab21?

[Response-10]

To address this issue, we analyzed dendrite length and the number of primary dendrites and dendrite branches in primary cultured cortical neurons. Because our data indicate that Rab21 and caveolin-1 regulate immature neurite pruning, which is an early

phase of neuronal maturation, we used primary neurons at 8 days-in-vitro, an early phase of dendrite maturation.

Although Rab21 knockdown slightly increased the number of primary dendrites, knockdown of Rab21 or caveolin-1 or Rab5 basically did not affect the dendrite morphologies of primary cortical neurons, which is consistent with the hypothesis that immature neurite pruning is only observed *in vivo*. Therefore, the dendrite maturation in cortical neurons differs between *in vitro* and *in vivo*. The results are shown in Fig. EV3D-E

4) I find the figures that use pink and red hard to see. The pink and red are too similar to easily distinguish. Could you put the Pearson co-efficients next to the pictures in the figures?

[Response-11]

As suggested by the reviewer, we added the Pearson's correlation coefficient graphs next to the corresponding images. The results are shown in Figures. 1B, 1G, 3C, 3G, 3I, 6I, EV1C, EV4D, EV4F, EV5B, EV5D, EV5I and EV6F.

Referee #3:

Shikanai et.al examines the role of the small GTPases, Rab5 and Rab21, in different types of endocytosis. They conclude that these GTPases differentially regulate clathrin- and caveolin-mediated endocytosis, and the regulation of caveolin-mediated endocytosis by Rab21 is required for neurite pruning.

Overall, the paper is interesting but there are conceptual and technical issues that needs to be addressed:

Conceptual:

1. One of the main claims of the paper is that Rab5 and 21 do not colocalize, which is in disagreement with previous studies (Egami & Araki, 2009, Simpson et al., 2004).

Can this lack of colocalization neurons specific ? In case the authors believe it is a general phenomenon they should show it in other cells.

[Response-12]

To address the issue brought up by the reviewer, we examined the localization of Rab21 and Rab5 in NIH3T3 fibroblasts. As previously reported, we found some overlapping staining signals of Rab21 and Rab5. The Pearson's correlation between Rab21 and Rab5 is 0.322, suggesting the low level colocalization of these proteins. However, we found that caveolin-1 preferentially colocalizes with Rab21, compared to Rab5, even in NIH3T3 cells, as shown in Fig. EV5C-D.

We obtained similar results in HeLa and COS-1 cells. The Pearson's correlation coefficients between caveolin-1 and Rab21 are 0.523 (HeLa cells) and 0.433 (COS-1 cells), whereas that between caveolin-1 and Rab5 are 0.359 (HeLa cells) and 0.197 (COS-1 cells). Thus, it appears that Rab21 also preferentially colocalizes with caveolin-1 in non-neuronal cells, although the colocalization efficiency depends on the cell lines. The results are shown in Fig. 3H and Fig. EV5A-B.

2. Same goes for the role of Rab5 in caveolin-mediated endocytosis that demonstrated in non-neuronal cells (Ariotti & Parton, 2013, Hagiwara et al., 2009, Hayer et al., 2010, Pelkmans et al., 2004).

[Response-13]

To address this issue, we performed LacCer uptake assay using NIH3T3 cells. Knockdown of Rab5 causes a slight decrease of the LacCer uptake. In contrast, Rab21 knockdown significantly reduces the LacCer uptake in NIH3T3 cells. Thus, although Rab5 may have a role in caveolin-mediated endocytosis in NIH3T3 fibroblasts, we find that Rab21 is a main contributor to caveolin-mediated endocytosis. The results are shown in Fig. EV5E-F.

3. Rescue of the Rab21 KD by caveolin OE. I am somehow surprised by these results. Does It means that the main role of Rab21 is to control the levels of caveolin and not localization ?

[Response-14]

Knockdown of Rab21 results in reduction of caveolin-1 protein levels, but we think this is a consequence of the mislocalization of caveolin-1. Our new data show that in the Rab21-knockdown neurons treated with Bafilomycin A1, a lysosomal inhibitor, many caveolin-1 proteins are observed in the lysosomes, suggesting that mislocalized caveolin-1 is transported to the lysosomes in Rab21-knockdown neurons.

In our current model, knockdown of Rab21 primarily decreases the caveolin-1 in the plasma membrane and increases the mislocalized caveolin-1, which is transported to the lysosomes and degraded. This results in a decrease in the protein levels of caveolin-1. The results are shown in Fig. 6A-I.

Regarding the rescue experiments, the remaining residual Rab21 may regulate overexpressed caveolin-1 to restore the phenotypes (because these are knockdown, not knockout, experiments). Caveolin-1 might also act as a scaffold protein to indirectly activate Rab21 because previous reports indicate that overexpression of caveolin-1 activates other small GTPases (J Cell Biol, 177, 683-694, 2007; J Cell Sci, 127, 2401-2406, 2014), although our results show that knockdown of caveolin-1 does not affect the plasma membrane localization of Rab21.

This explanation is now included in the revised manuscript as follows.

"Co-expression of wt-caveolin-1, which is expected to increase the overall cellular caveolin-1 protein levels and possibly also in the plasma membrane, as a low-level Rab21 expression remained in the knockdown cells, dramatically restored the immature neurite pruning in the Rab21 knockdown neurons (Fig. 7A-B). Caveolin-1 might also act as a scaffold protein to indirectly activate Rab21 because previous reports indicate that overexpression of caveolin-1 activates other small GTPases (Diaz *et al*, 2014; Grande-Garcia *et al*, 2007), although our results show that knockdown of caveolin-1 does not affect the plasma membrane localization of Rab21 (Fig. EV5M-N)."

Technical:

1. Overall, the colocalization experiments are not of the highest quality but they are largely convincing. In 2C both Rabs doesn't seem to strongly localized with the PM.

[Response-15]

To address this issue, we provide quantitative data using Pearson's correlation coefficient. The results are shown in Figures. 1B, 1G, 3C, 3G, 3I, 6I, EV1C, EV4D, EV4F, EV5B, EV5D, EV5I and EV6F.

Rab21 is strongly localized not only in the plasma membrane but also in the early endosomes. Because Rab21 is also weakly localized in other organelles such as the ER, its cytosolic signals may be noticeable in the confocal section. Our quantitative data indicate that the Pearson's correlation coefficients are 0.528 (Rab21 and the plasma membrane) and 0.558 (Rab21 and the early endosomes).

The explanation of Rab21 and caveolin-1 localization is now included in the revised manuscript as follows.

"Endogenous Rab21 and caveolin-1 were found to be colocalized near the plasma membrane (as visualized with transfected plasma membrane-targeted monomeric Azami-Green 1 (PM-mAG1)) (Fig. 3B). The Pearson's correlation coefficient between PM-mAG1 and Rab21 or caveolin-1 is 0.528 and 0.405, respectively, and the Pearson's correlation coefficient between Rab21 and caveolin-1 was higher than that of Rab5 and caveolin-1 in whole cells (Rab21: 0.660, Rab5: 0.264) and at the plasma membrane (Rab21: 0.620, Rab5: 0.203) (Fig. 3C). These data indicate that Rab21 preferentially colocalizes with caveolin-1 at the plasma membrane.

The colocalization of Rab21 and caveolin-1 was also observed in the vesicular components (Fig. 3B lower panels). Because both Rab21 and caveolin-1 were localized at the early endosomes (Figs. EV1B-C and EV4E-F), the vesicular components near the plasma membrane may be early endosomes. We observed some localization of Rab21 in the ER, which was visualized with anti-calnexin or anti-KDEL antibodies (Fig. EV4C-D), as previously reported (Opdam *et al*, 2000). However, caveolin-1 was rarely observed in the ER (Fig. EV4E-F), suggesting that cooperation between Rab21 and caveolin-1 does not occur in the ER."

2. How the authors can be sure that Rab21 and caveolin are present in the same vesicular compartment ?

[Response-16]

Rab21 and caveolin-1 are colocalized in primary cultured neurons (Pearson's correlation coefficient = 0.660) and immature neurons in the developing cerebral cortex (Pearson's correlation coefficient = 0.612). Our high-resolution microscopy analyses indicate that both Rab21 and caveolin-1 exhibit a vesicular-like localization, suggesting that Rab21 and caveolin-1 are colocalized in the same vesicular compartment (Fig. 3F).

However, as the reviewer pointed out, it is difficult to quantify these results, and therefore we have modified our statements in the revised manuscript as follows.

"High resolution microscopy analyses revealed that Rab21 and caveolin-1 were sometimes present on the same vesicular compartments in the immature neurons (Fig. 3F)."

3. looking at the EV3D it does not look like pruning defect to me. Indeed, in the control there is elimination of immature neurites. But I am not sure the same neurites hang around in the Rab21-sh for the entire time. Looks to me that some are eliminated and new ones are formed.

[Response-17]

We and others have previously reported that multipolar-shaped immature cortical neurons frequently extend and retract their neurites, which is a major characteristic of the multipolar neurons. In contrast, coincident with the leading process formation, immature neurite pruning occurs and additional neurite extension is suppressed (Nature Neurosci, 7, 136-144, 2004; J Neurosci, 23, 9996-10001, 2003; Nature Cell Biol, 8, 17-26, 2006; J Biol Chem, 285, 5878-5887, 2010). Thus, prior to leading process formation, control neurons also retract and extend their immature neurites. However, in Rab21-knockdown neurons, even after the leading process formation, we observed the continual extension and retraction of many neurites, similar that of immature neurites of the multipolar neurons.

As the reviewer pointed out, our original description was confusing. In the revised manuscript, we have tried to clarify by adding the following sentences.

"Our time-lapse imaging analyses showed that these abnormal neurites frequently extended and retracted, which is a major characteristic of the immature neurites of the multipolar-shaped neurons. This suggests that these additional neurites of Rab21-knockdown neurons are derived from the immature neurites."

4. The internalization experiments in Fig2 and EV4 require rescue experiments.

[Response-18]

As suggested by the reviewer, we have added rescue experiments for Rab21 knockdown to the original Fig. 2 and Fig. EV4 (corresponding to Fig. 4A-B and Fig. 4E-F in the revised version of the manuscript, respectively).

5. Fig 3C only 20% effect - is this minor effect can drive the biology?

[Response-19]

Cell surface levels of N-cadherin are increased by 24% in the Rab21-knockdown primary cortical neurons, but the ratio of N-cadherin in the plasma membrane is increased by 40% *in vivo* (Fig. 5 in the revised manuscript). In addition, knockdown of Rab21 also disturbs the early endosomal trafficking. Together with our immunohistochemical data showing that the HA-N-cadherin staining signals are increased in the immature neurites of the Rab21-knockdown cortical neurons (Fig. 5G), we believed that the amount of abnormally increased N-cadherin is sufficient to elicit the observed defects in immature neurite pruning. This hypothesis is also supported by our results showing that weak reduction of N-cadherin can rescue the immature neurite pruning defects of Rab21-knockdown neurons (Fig. EV6A-B).

6. Fig 5A not really resembling the phenotype in 1D

[Response-20]

As the reviewer pointed out, the image of the Rab21-knockdown neurons in Fig. 5A in the original version of the manuscript was obtained with lower laser power (and/or low gain), and thereby it is hard to recognize the abnormally extended immature neurites from the cell soma.

In the revised manuscript, we have replaced the image with a new neuron displaying more typical morphology. The results are shown in Fig. 7A (corresponding to the original Fig. 5A).

Minor:

1. The term sticky neurons is no clear

[Response-21]

We have revised that term with “the immature neurites of Rab21 knockdown multipolar-shaped neurons were sometimes observed to be attached to one another”.

2. Fig 3D-G please interest the Y axis.

[Response-22]

As the reviewer pointed out, the cell surface levels of N-cadherin in neurons *in vitro* and *in vivo* are different. This may be because cell-cell adhesion is not much observed in primary cortical neurons *in vitro*. Cadherins in the cell-cell adhesion complex are generally stable, and the cell surface levels of N-cadherin may be low in primary cortical neurons, compared with immature neurons *in vivo*.

According to the reviewer’s suggestion, we discuss this point in the revised manuscript as follows.

“The ratio of the cell surface N-cadherin in control immature neurons *in vivo* was higher than that *in vitro*, possibly because cell-to-cell adhesion is not much observed in primary cortical neurons *in vitro*. Importantly, colocalization of HA-tagged N-cadherin and PM-mAG1 was further increased in the Rab21-sh115-electroporated immature neurons (Fig. 5G-H), suggesting that Rab21 regulates N-cadherin endocytosis in the immature neurons *in vivo*.”

1st Revision - Editorial Decision 13th Oct 2022

Dear Dr. Kawauchi

Thank you for the submission of your revised manuscript to EMBO reports. We have now received the full set of referee reports that is copied below.

As you will see, all referees are very positive about the study and request only minor changes to clarify text and some interpretations and to more carefully phrase some of the conclusions.

From the editorial side, there are also a few things that we need before we can proceed with the official acceptance of your study.

- Please provide all figures as individual production quality files as .eps, .tif, .jpg (one file per figure).
Please download our Figure Preparation Guidelines (figure preparation pdf) from our Author Guidelines pages https://www.embopress.org/page/journal/14693178/authorguide for more info on how to prepare your figures.

- I should add that we recommend arranging the figures so that the figure panels can be called out in an alphabetical order, which is currently not the case. Where possible, please rearrange.

- Please note that we can only typeset up to 5 EV figure; you currently have 7. You can either merge some figures to reduce their number to 5, move 2 EV figures to an Appendix or alternatively, convert all EV figures into and Appendix.
- The Appendix is a single PDF file that contains the figures and their legends, which starts with a short Table of Content incl. page numbers. Appendix figures should be referred to in the main text as: "Appendix Figure S1, Appendix Figure S2" etc. See detailed instructions regarding expanded view here:
<https://www.embopress.org/page/journal/14693178/authorguide#expandedview>

- Please update the 'Conflict of interest' paragraph to our new 'Disclosure and competing interests statement'. For more information see
https://www.embopress.org/page/journal/14693178/authorguide#conflictsofinterest

- Regarding the Author Contributions, we now use CRediT to specify the contributions of each author in the journal submission system. CRediT replaces the author contribution section, which needs to be removed from the manuscript. Please use the free text box in our online submission system to provide more detailed descriptions if you wish. See also guide to authors https://www.embopress.org/page/journal/14693178/authorguide#authorshipguidelines.

- Please state the ethical committee that approved the animal work in the materials and methods section. Currently, you only refer to guidelines established by Keio University.

- I attach to this email a related manuscript file with comments by our data editors. Please address all comments and upload a revised file with tracked changes with your final manuscript submission. Please also keep in mind that statistical analysis should only be performed on data from more than 3 independent experiments and that cells in a culture dish do not conform with this. Thus in case the analysed cells (e.g. in Fig. EV7) are not from different experiments, the individual data points should be shown instead of mean or average values.

- As a standard procedure, we edit the title and abstract of manuscripts to make them more accessible to a general readership. Please find my suggestions also in the attached manuscript file.

- Author checklist: please indicate in which section of the manuscript the information is available and please complete the question on Dual Use Research of Concern.

- Finally, EMBO reports papers are accompanied online by A) a short (1-2 sentences) summary of the findings and their significance, B) 2-3 bullet points highlighting key results and C) a synopsis image that is 550x300-600 pixels large (width x height) in PNG for JPG format. You can either show a model or key data in the synopsis image. Please note that the size is rather small and that text needs to be readable at the final size. Please send us this information along with the revised manuscript.

We look forward to seeing a final version of your manuscript as soon as possible.

With kind regards,

Martina Rembold, PhD

Senior Editor
EMBO reports

****

Referee #1:

The reviewers satisfactorily addressed my main criticisms.

Referee #2:

This is a revised manuscript. The authors report interesting findings that Rab5 and Rab21 regulate endocytosis into early endosomes differently. In particular, downregulation of Rab21 affects caveolin 1 stability and thus caveolin-dependent endocytic pathways. The authors previously showed a role for caveolin 1 in regulating pruning of processes in multipolar neurons in the developing cortex prior to becoming bipolar and migratory. Interestingly, Rab21 also regulates early developmental pruning and is upstream of caveolin1. The regulation of this pruning step is poorly understood and this manuscript makes an important contribution to this field. The authors addressed most of the reviewers' suggestions, including Pearson coefficients, dendrite development in cultured neurons. I have no major issues at this point. A minor issue is that the paragraph about the phenotype of shRab21 in dendrite morphology in cultured cortical neurons is somewhat misleading. The authors use the term "rarely" in the subheading which is not the right term. The data show that there are more primary dendrites. It also appears from the images that shCaveolin1 causes sad-looking dendrites. Is the experiment sufficiently powered to see a statistical difference? These data are interesting and should be more fully acknowledged. It feels like the authors are trying to downplay the effects they have because they want to argue that dendrite pruning occurs only in vivo. It does not take away from the conclusion if Rab21 also plays a role in cultured neurons. I also recommend that all single channel panels should be shown in black and white. It is really hard to see the blue alone channels. I recommend publication.

Referee #3:

Shikanai et.al made a big experimental effort to address the comments that I raised.
My only remining concern is that they still overstate/ mispresent some of the results.

Specifically:

1. The colocalization of Rab5 and 21 seems like an open issue, for example the Pearson's correlation for caveolin-1 and Rab5 is 0.359 (HeLa cells), seems high to me and I am not sure it is really different from the 0.523 for rab21. Same for the LacCer uptake assay there is an effect for Rab5 KD. Maybe tagging of the endogenous proteins could clear things but with the current data lack of Rab5 and 21 colocalization seems like overstatement, and the authors should tone it down most importantly in the abstract

2. In their response to my comment on EV3D the authors write that" However, in Rab21-knockdown neurons, even after the leading process formation, we observed the continual extension and retraction of many neurites, similar that of immature neurites of the multipolar neurons".
This is an interesting phenotype; it seems that the process of extension and retraction of neurites that is suppressed by the formation of a leading process in WT neurons continue in the Rab21-knockdown neurons. BUT this is NOT a pruning defect as stated in the abstract and the title

Point-by-point responses to the editorial and reviewers' comments

MS. No.: EMBOR-2022-54701V2

Our point-by-point response to all the issues raised by the editor(s) and reviewers is provided below. For your convenience, the editor's and the editors' and reviewers' comments are shown below in italic and bold.

[Editorial comments]

- Please provide all figures as individual production quality files as .eps, .tif, .jpg (one file per figure). Please download our Figure Preparation Guidelines (figure preparation pdf) from our Author Guidelines pages https://www.embopress.org/page/journal/14693178/authorguide for more info on how to prepare your figures.

[Response-1]

According to the editorial comments, we have prepared and uploaded all figures as single TIFF files with 300 pixel per inch (RGB).

- I should add that we recommend arranging the figures so that the figure panels can be called out in an alphabetical order, which is currently not the case. Where possible, please rearrange.

[Response-2]

We tried our best to arrange the figure panels in alphabetical order.

- Please note that we can only typeset up to 5 EV figure; you currently have 7. You can either merge some figures to reduce their number to 5, move 2 EV figures to an Appendix or alternatively, convert all EV figures into and Appendix.

[Response-3]

Rearrangement of the figures results in 5 EV figures and 1 appendix, as well as 7 main figures.

- The Appendix is a single PDF file that contains the figures and their legends, which starts with a

short Table of Content incl. page numbers. Appendix figures should be referred to in the main text as: "Appendix Figure S1, Appendix Figure S2" etc. See detailed instructions regarding expanded view here:
<https://www.embopress.org/page/journal/14693178/authorguide#expandedview&gt;

[Response-4]
Our manuscript includes only one Appendix figure, but we added a short Table of Content page before Appendix Figure S1.

- Please update the 'Conflict of interest' paragraph to our new 'Disclosure and competing interests statement'. For more information see
https://www.embopress.org/page/journal/14693178/authorguide#conflictsofinterest

[Response-5]
“Conflict of interest” was moved into “Disclosure and competing interests statements” in the revised manuscript.

- Regarding the Author Contributions, we now use CRediT to specify the contributions of each author in the journal submission system. CRediT replaces the author contribution section, which needs to be removed from the manuscript. Please use the free text box in our online submission system to provide more detailed descriptions if you wish. See also guide to authors https://www.embopress.org/page/journal/14693178/authorguide#authorshipguidelines.

[Response-6]
We have removed the Author Contributions and replaced it with CRediT in the revised manuscript.

- Please state the ethical committee that approved the animal work in the materials and methods section. Currently, you only refer to guidelines established by Keio University.

[Response-7]
Thank you for your suggestion. We added the statements that the ethical committees of Kyoto University and Tohoku Medical and Pharmaceutical University approved the animal work. In the original version of the Materials and Methods, “Keio University”, “RIKEN Center for

Developmental Biology" and "Institute of Biomedical Research and Innovation", which were all different institutes, approved the animal work. Because "RIKEN Center for Developmental Biology" has been renamed into "RIKEN BDR" and "Institute of Biomedical Research and Innovation" is a subsidiary institute of FBRI, we have corrected this.

- I attach to this email a related manuscript file with comments by our data editors. Please address all comments and upload a revised file with tracked changes with your final manuscript submission. Please also keep in mind that statistical analysis should only be performed on data from more than 3 independent experiments and that cells in a culture dish do not conform with this. Thus in case the analysed cells (e.g. in Fig. EV7) are not from different experiments, the individual data points should be shown instead of mean or average values.

[Response-8]
According to the editorial comments in the manuscript file, we defined the nature of the replications as "n" and added the explanation for the box-and-whisker plots in the figure legends (although most of the box-and-whisker plots have been replaced as described below).
Regarding statistical analyses, we have replaced the box-and-whisker plots with the bar graphs showing the individual data points.
Please note that we used "track changes", when rewriting the manuscript. Because the EV Figures have largely been rearranged (see the Response-3 and -4), most of the figure legends appear seemingly revised, but basically, we have only changed the issues requested by the editors.

- As a standard procedure, we edit the title and abstract of manuscripts to make them more accessible to a general readership. Please find my suggestions also in the attached manuscript file.

[Response-9]
Thank you very much for revising the Abstract. We think it has been improved.

- Author checklist: please indicate in which section of the manuscript the information is available and please complete the question on Dual Use Research of Concern.

[Response-10]

We completed “Dual Use Research of Concern” in the Author Checklist.

- Finally, EMBO reports papers are accompanied online by A) a short (1-2 sentences) summary of the findings and their significance, B) 2-3 bullet points highlighting key results and C) a synopsis image that is 550x300-600 pixels large (width x height) in PNG for JPG format. You can either show a model or key data in the synopsis image. Please note that the size is rather small and that text needs to be readable at the final size. Please send us this information along with the revised manuscript.

[Response-11]
We have uploaded the short summary, highlight and synopsis image.

****

Referee #1:
The reviewers satisfactorily addressed my main criticisms.

Referee #2:
This is a revised manuscript. The authors report interesting findings that Rab5 and Rab21 regulate endocytosis into early endosomes differently. In particular, downregulation of Rab21 affects caveolin 1 stability and thus caveolin-dependent endocytic pathways. The authors previously showed a role for caveolin 1 in regulating pruning of processes in multipolar neurons in the developing cortex prior to becoming bipolar and migratory. Interestingly, Rab21 also regulates early developmental pruning and is upstream of caveolin1. The regulation of this pruning step is poorly understood and this manuscript makes an important contribution to this field. The authors addressed most of the reviewers' suggestions, including Pearson coefficients, dendrite development in cultured neurons. I have no major issues at this point. A minor issue is that the paragraph about the phenotype of shRab21 in dendrite morphology in cultured cortical neurons is somewhat misleading. The authors use the term "rarely" in the subheading which is not the right term. The data show that there are more primary dendrites. It also appears from the images that shCaveolin1 causes sad-looking dendrites. Is the experiment sufficiently powered to

see a statistical difference? These data are interesting and should be more fully acknowledged. It feels like the authors are trying to downplay the effects they have because they want to argue that dendrite pruning occurs only in vivo. It does not take away from the conclusion if Rab21 also plays a role in cultured neurons. I also recommend that all single channel panels should be shown in black and white. It is really hard to see the blue alone channels. I recommend publication.

[Response-12]
According to the reviewer's suggestion, we have revised the manuscript, as follows. We changed the subheading into "*Rab21 is partly involved in dendrite maturation in vitro*".

"Rab21-knockdown slightly but significantly increased the number of primary dendrites, which may resemble the *in vivo* phenotypes of Rab21-sh115-electroporated neurons. In contrast, no differences were observed in the dendrite length and branch number between control and Rab21- or caveolin-1- or Rab5-knockdown neurons. Although immature neurite pruning, the early step of neuronal maturation that requires Rab21 and caveolin-1, is only observed *in vivo*, primary cortical neurons may mimic the molecular machinery of *in vivo* neuronal maturation at least in part."

Regarding the blue alone channels, we have changed all blue channels into black and white. When all single channels are changed into black and white, it is difficult to recognize which is a green (or red) image. Therefore, with reference to the previously published papers on EMBO Rep (EMBO Rep, e55470, 2022; EMBO Rep, e54421, 2022), we changed only blue alone channels.

Referee #3:
Shikanai et.al made a big experimental effort to address the comments that I raised.
My only remining concern is that they still overstate/ mispresent some of the results.

Specifically:

1. The colocalization of Rab5 and 21 seems like an open issue, for example the Pearson's correlation for caveolin-1 and Rab5 is 0.359 (HeLa cells), seems high to me and I am not sure it is really different from the 0.523 for rab21. Same for the LacCer uptake assay there is an effect for Rab5 KD. Maybe tagging of the endogenous proteins could clear things but with the current data lack of Rab5 and 21 colocalization seems like overstatement, and the authors should tone it down

most importantly in the abstract

[Response-13]

As the reviewer pointed out, it is difficult to determine the criteria for colocalization in HeLa, NIH3T3 and COS-1 cells. Therefore, we only mentioned the experimental results of the colocalization and LacCer uptake assays in cultured non-neuronal cells; Pearson's correlation coefficient values and the results of statistical analyses. Because colocalization between Rab21 and Rab5 was relatively high in NIH3T3 cells (the Pearson's correlation coefficient is 0.32), we have modified the statements into "some overlap between Rab21 and Rab5 was observed in NIH3T3 cells, as previously reported".

Regarding primary cortical neurons, colocalization of Rab21 and caveolin-1 is significantly higher than that of Rab5 and caveolin-1, and the Pearson's correlation coefficients differ by more than 2.5 fold (Rab21: 0.66, Rab5: 0.26). Moreover, the Pearson's correlation coefficient of Rab5-Rab21 is less than 0.3 (0.25), which is similar to that of Rab5-Lamp1 (a negative control: 0.28). Thus, at least in primary cortical neurons, we can conclude that Rab21 preferentially colocalizes with caveolin-1, compared with Rab5 and that colocalization between Rab21 and Rab5 is low.

2. In their response to my comment on EV3D the authors write that" However, in Rab21-knockdown neurons, even after the leading process formation, we observed the continual extension and retraction of many neurites, similar that of immature neurites of the multipolar neurons".

This is an interesting phenotype; it seems that the process of extension and retraction of neurites that is suppressed by the formation of a leading process in WT neurons continue in the Rab21-knockdown neurons. BUT this is NOT a pruning defect as stated in the abstract and the title

[Response-14]

The immature neurites exhibit an acute retraction before the transition into the leading process-possessing bipolar neurons. We have to distinguish this phenomenon from a "neurite retraction", because immature neurites constantly exhibit extension and retraction. Due to the disappearance of all immature neurites, this acute (and complete) immature neurite retraction has been called "immature neurite pruning" in previous papers. The defect in this acute disappearance of the immature neurites was observed in caveolin-1-knockdown neurons and referred to as a "immature neurite pruning defect" (iScience, Vol.7, 53-67, 2018; J Cell Sci, Vol.133, jcs241562, 2020; FEBS J, Vol.289, 2219-2246, 2022). In this manuscript, we conclude that Rab21 regulates

caveolin-1 and knockdown of Rab21 exhibits the same phenotypes seen in caveolin-1 knockdown. Because different terms for the same phenotype may lead to misunderstanding, we believe that the term, "immature neurite pruning defect", should be applied to the description of the Rab21-knockdown phenotype, in accordance with the previous papers.
The editor has suggested that we *keep the word pruning but to justify it when you first describe it*. Therefore, we modified the description as follows.

"Although immature neurites turn into dendrites *in vitro* (Dotti *et al*, 1988), *in vivo* cortical neurons undergo immature neurite pruning, an acute retraction process of immature neurites that is mediated by caveolin-1 (Shikanai *et al*. 2018), and form a thick leading process, followed by long-distance neuronal migration along the radial fibers." (from Introduction)

"We did not observe an acute retraction of the immature neurites in Rab21-knockdown neurons, which exhibit the phenotype seen in caveolin-1-knockdown neurons (Shikanai et al. 2018), and therefore we referred to this phenotype as an immature neurite pruning defect." (from Results)

Dr. Takeshi Kawauchi
Graduate School of Medicine, Kyoto University
Department of Aging Science and Medicine
Medical Innovation Center, 53 Kawahara-cho, Shogoin
Sakyo-ku
Kyoto 606-8507
Japan

Dear Dr. Kawauchi,

I am very pleased to accept your manuscript for publication in the next available issue of EMBO reports. Thank you for your contribution to our journal.

At the end of this email I include important information about how to proceed. Please ensure that you take the time to read the information and complete and return the necessary forms to allow us to publish your manuscript as quickly as possible.

As part of the EMBO publication's Transparent Editorial Process, EMBO reports publishes online a Review Process File to accompany accepted manuscripts. As you are aware, this File will be published in conjunction with your paper and will include the referee reports, your point-by-point response and all pertinent correspondence relating to the manuscript.

If you do NOT want this File to be published, please inform the editorial office within 2 days, if you have not done so already, otherwise the File will be published by default [contact: emboreports@embo.org]. If you do opt out, the Review Process File link will point to the following statement: "No Review Process File is available with this article, as the authors have chosen not to make the review process public in this case." Please note that the author checklist will still be published even if you opt out of the transparent process.

Thank you again for your contribution to EMBO reports and congratulations on a successful publication. Please consider us again in the future for your most exciting work.

Yours sincerely,

Martina Rembold, PhD
Senior Editor
EMBO reports

****

THINGS TO DO NOW:

Once your article has been received by Wiley for production, the corresponding author will receive an email from Wiley's Author Services system which will ask them to log in and will present them with the appropriate license for completion.

You will receive proofs by e-mail approximately 2-3 weeks after all relevant files have been sent to our Production Office; you should return your corrections within 2 days of receiving the proofs.

Please inform us if there is likely to be any difficulty in reaching you at the above address at that time. Failure to meet our deadlines may result in a delay of publication, or publication without your corrections.

All further communications concerning your paper should quote reference number EMBOR-2022-54701V3 and be addressed to emboreports@wiley.com.

Should you be planning a Press Release on your article, please get in contact with emboreports@wiley.com as early as possible, in order to coordinate publication and release dates.

EMBO Press Author Checklist

Corresponding Author Name: Takeshi Kawauchi	
Journal Submitted to: EMBO Reports	
Manuscript Number: EMBOR-2022-54701V1	

USEFUL LINKS FOR COMPLETING THIS FORM
The EMBO Journal - Author Guidelines
EMBO Reports - Author Guidelines
Molecular Systems Biology - Author Guidelines
EMBO Molecular Medicine - Author Guidelines

Reporting Checklist for Life Science Articles (updated January 2022)
This checklist is adapted from Materials Design Analysis Reporting (MDAR) Checklist for Authors. MDAR establishes a minimum set of requirements in transparent reporting in the life sciences (see Statement of Task: 10.31222/osf.io/9sm4x). Please follow the journal's guidelines in preparing your manuscript.
Please note that a copy of this checklist will be published alongside your article.

Abridged guidelines for figures
1. Data
The data shown in figures should satisfy the following conditions:
- the data were obtained and processed according to the field’s best practice and are presented to reflect the results of the experiments in an accurate and unbiased manner.
- ideally, figure panels should include only measurements that are directly comparable to each other and obtained with the same assay.
- plots include clearly labeled error bars for independent experiments and sample sizes. Unless justified, error bars should not be shown for technical replicates.
- if n<5, the individual data points from each experiment should be plotted. Any statistical test employed should be justified.
- Source Data should be included to report the data underlying figures according to the guidelines set out in the authorship guidelines on Data Presentation.

2. Captions
Each figure caption should contain the following information, for each panel where they are relevant:
- a specification of the experimental system investigated (eg cell line, species name).
- the assay(s) and method(s) used to carry out the reported observations and measurements.
- an explicit mention of the biological and chemical entity(ies) that are being measured.
- an explicit mention of the biological and chemical entity(ies) that are altered/varied/perturbed in a controlled manner.
- the exact sample size (n) for each experimental group/condition, given as a number, not a range;
- a description of the sample collection allowing the reader to understand whether the samples represent technical or biological replicates (including how many animals, litters, cultures, etc.).
- a statement of how many times the experiment shown was independently replicated in the laboratory.
- definitions of statistical methods and measures:
  - common tests, such as t-test (please specify whether paired vs. unpaired), simple χ2 tests, Wilcoxon and Mann-Whitney tests, can be unambiguously identified by name only, but more complex techniques should be described in the methods section;
  - are tests one-sided or two-sided?
  - are there adjustments for multiple comparisons?
  - exact statistical test results, e.g., P values = x but not P values < x;
  - definition of ‘center values’ as median or average;
  - definition of error bars as s.d. or s.e.m.

Please complete ALL of the questions below.
Select "Not Applicable" only when the requested information is not relevant for your study.

Materials

Newly Created Materials	Information included in the manuscript?	In which section is the information available? (Reagents and Tools Table, Materials and Methods, Figures, Data Availability Section)
New materials and reagents need to be available; do any restrictions apply?	Not Applicable	

Antibodies	Information included in the manuscript?	In which section is the information available? (Reagents and Tools Table, Materials and Methods, Figures, Data Availability Section)
For **antibodies** provide the following information:<br>- Commercial antibodies: RRID (if possible) or supplier name, catalogue number and or/clone number<br>- Non-commercial: RRID or citation	Yes	"Antibodies and chemical regents" section in Materials and Methods

DNA and RNA sequences	Information included in the manuscript?	In which section is the information available? (Reagents and Tools Table, Materials and Methods, Figures, Data Availability Section)
**Short novel DNA or RNA including primers, probes**: provide the sequences.	Yes	"Plasmids" section in Materials and Methods

Cell materials	Information included in the manuscript?	In which section is the information available? (Reagents and Tools Table, Materials and Methods, Figures, Data Availability Section)
**Cell lines:** Provide species information, strain. Provide accession number in repository **OR** supplier name, catalog number, clone number, and/**OR** RRID.	Yes	"Cell line culture and transfection" section in Materials and Methods
**Primary cultures:** Provide species, strain, sex of origin, genetic modification status.	Yes	"In utero electroporation" and "Primary cultures, transfection and immunocytochemistry" sections in Materials and Methods. Note that we used embryonic brains and therefore could not detemine the sex of origin.
Report if the cell lines were recently **authenticated** (e.g., by STR profiling) and tested for mycoplasma contamination.	Yes	We used the cell lines within 5 times passages after given by the supplier, which was mentioned in "Cell line culture and transfection" section in Materials and Methods.

Experimental animals	Information included in the manuscript?	In which section is the information available? (Reagents and Tools Table, Materials and Methods, Figures, Data Availability Section)
**Laboratory animals or Model organisms:** Provide species, strain, sex, age, genetic modification status. Provide accession number in repository **OR** supplier name, catalog number, clone number, **OR** RRID.	Yes	"In utero electroporation" section in Materials and Methods.
**Animal observed in or captured from the field:** Provide species, sex, and age where possible.	Not Applicable	
Please detail **housing and husbandry conditions**.	Not Applicable	

Plants and microbes	Information included in the manuscript?	In which section is the information available? (Reagents and Tools Table, Materials and Methods, Figures, Data Availability Section)
**Plants:** provide species and strain, ecotype and cultivar where relevant, unique accession number if available, and source (including location for collected wild specimens).	Not Applicable	
**Microbes:** provide species and strain, unique accession number if available, and source.	Not Applicable	

Human research participants	Information included in the manuscript?	In which section is the information available? (Reagents and Tools Table, Materials and Methods, Figures, Data Availability Section)
If collected and within the bounds of privacy constraints report on age, sex and gender or ethnicity for all study participants.	Not Applicable	

Core facilities	Information included in the manuscript?	In which section is the information available? (Reagents and Tools Table, Materials and Methods, Figures, Data Availability Section)
If your work benefited from core facilities, was their service mentioned in the acknowledgments section?	Yes	We mentioned this in Acknowledgements.

Design

Study protocol	Information included in the manuscript?	In which section is the information available? (Reagents and Tools Table, Materials and Methods, Figures, Data Availability Section)
If study protocol has been **pre-registered, provide DOI in the manuscript**. For clinical trials, provide the trial registration number **OR** cite DOI.	Not Applicable	
Report the **clinical trial registration number** (at ClinicalTrials.gov or equivalent), where applicable.	Not Applicable	

Laboratory protocol	Information included in the manuscript?	In which section is the information available? (Reagents and Tools Table, Materials and Methods, Figures, Data Availability Section)
Provide DOI OR other citation details if **external detailed step-by-step protocols** are available.	Not Applicable	

Experimental study design and statistics	Information included in the manuscript?	In which section is the information available? (Reagents and Tools Table, Materials and Methods, Figures, Data Availability Section)
Include a statement about **sample size** estimate even if no statistical methods were used.	Yes	Sample size was mentioned in Figure legends.
Were any steps taken to minimize the effects of subjective bias when allocating animals/samples to treatment (e.g. **randomization procedure**)? If yes, have they been described?	Not Applicable	
Include a statement about **blinding** even if no blinding was done.	Yes	Blinding was mentioned in Figure legends.
Describe **inclusion/exclusion criteria** if samples or animals were excluded from the analysis. Were the criteria pre-established?<br><br>If sample or data points were omitted from analysis, report if this was due to attrition or intentional exclusion and provide justification.	Not Applicable	
For every figure, are **statistical tests** justified as appropriate? Do the data meet the assumptions of the tests (e.g., normal distribution)? Describe any methods used to assess it. Is there an estimate of variation within each group of data? Is the variance similar between the groups that are being statistically compared?	Yes	It was mentioned in "Statistical analyses" section in Materials and Methods.

Sample definition and in-laboratory replication	Information included in the manuscript?	In which section is the information available? (Reagents and Tools Table, Materials and Methods, Figures, Data Availability Section)
In the figure legends: state number of times the experiment was **replicated** in laboratory.	Yes	It was mentioned in Figure legends.
In the figure legends: define whether data describe **technical or biological replicates**.	Yes	It was mentioned in Figure legends.

Ethics

Ethics	Information included in the manuscript?	In which section is the information available? (Reagents and Tools Table, Materials and Methods, Figures, Data Availability Section)
Studies involving **human participants**: State details of **authority granting ethics approval** (IRB or equivalent committee(s), provide reference number for approval.	Not Applicable	
Studies involving **human participants**: Include a statement confirming that **informed consent** was obtained from all subjects and that the experiments conformed to the principles set out in the WMA Declaration of Helsinki and the Department of Health and Human Services Belmont Report.	Not Applicable	
Studies involving **human participants:** For publication of **patient photos**, include a statement confirming that consent to publish was obtained.	Not Applicable	
Studies involving experimental **animals**: State details of **authority granting ethics approva**l (IRB or equivalent committee(s), provide reference number for approval. Include a statement of compliance with ethical regulations.	Yes	"In utero electroporation" section in Materials and Methods.
Studies involving **specimen and field samples:** State if relevant **permits** obtained, provide details of authority approving study; if none were required, explain why.	Not Applicable	

Dual Use Research of Concern (DURC)	Information included in the manuscript?	In which section is the information available? (Reagents and Tools Table, Materials and Methods, Figures, Data Availability Section)
Could your study fall under dual use research restrictions? Please check biosecurity documents and list of **select agents and toxins** (CDC): https://www.selectagents.gov/sat/list.htm	Not Applicable	In this study, we did not use the "select agents and toxins", listed in the webpage.
If you used a select agent, is the security level of the lab appropriate and reported in the manuscript?	Not Applicable	
If a study is subject to dual use research of concern regulations, is the name of the **authority granting approval and reference number** for the regulatory approval provided in the manuscript?	Not Applicable	

Reporting

The MDAR framework recommends adoption of discipline-specific guidelines, established and endorsed through community initiatives. Journals have their own policy about requiring specific guidelines and recommendations to complement MDAR.

Adherence to community standards	Information included in the manuscript?	In which section is the information available? (Reagents and Tools Table, Materials and Methods, Figures, Data Availability Section)
State if relevant guidelines or checklists (e.g., **ICMJE, MIBBI, ARRIVE, PRISMA**) have been followed or provided.	Not Applicable	
For **tumor marker prognostic studies,** we recommend that you follow the **REMARK** reporting guidelines (see link list at top right). See author guidelines, under 'Reporting Guidelines'. Please confirm you have followed these guidelines.	Not Applicable	
For **phase II and III randomized controlled trials**, please refer to the **CONSORT** flow diagram (see link list at top right) and submit the CONSORT checklist (see link list at top right) with your submission. See author guidelines, under 'Reporting Guidelines'. Please confirm you have submitted this list.	Not Applicable	

Data Availability

Data availability	Information included in the manuscript?	In which section is the information available? (Reagents and Tools Table, Materials and Methods, Figures, Data Availability Section)
Have **primary datasets** been deposited according to the journal's guidelines (see 'Data Deposition' section) and the respective accession numbers provided in the Data Availability Section?	Not Applicable	
Were **human clinical and genomic datasets** deposited in a public access-controlled repository in accordance to ethical obligations to the patients and to the applicable consent agreement?	Not Applicable	
Are **computational models** that are central and integral to a study available without restrictions in a machine-readable form? Were the relevant accession numbers or links provided?	Not Applicable	
If publicly available data were reused, provide the respective **data citations in the reference list**.	Not Applicable	
